# Supplementary material for: Why did some parents not send their children back to school following school closures during the COVID-19 pandemic: a cross-sectional survey
Source: BMJ Paediatr Open. 2021 Sep 29;5(1):e001014. doi: 10.1136/bmjpo-2020-001014 (PMC8482536; doi:10.1136/bmjpo-2020-001014)
Supplement: Author's manuscript [file bmjpo-2020-001014.draft_revisions.pdf]

BMJ Paediatrics Open

BMJ Paediatrics Open is committed to open peer review. As part of this commitment we make the peer review history of every article we publish publicly available.

When an article is published we post the peer reviewers' comments and the authors' responses online. We also post the versions of the paper that were used during peer review. These are the versions that the peer review comments apply to.

The versions of the paper that follow are the versions that were submitted during the peer review process. They are not the versions of record or the final published versions. They should not be cited or distributed as the published version of this manuscript.

BMJ Paediatrics Open is an open access journal and the full, final, typeset and author-corrected version of record of the manuscript is available on our site with no access controls, subscription charges or pay-per-view fees (http://bmjpaedsopen.bmj.com).

If you have any questions on BMJ Paediatrics Open's open peer review process please email info.bmjpo@bmj.com

BMJ Paediatrics Open

Parents' willingness to send children back to school during the COVID-19 pandemic: a cross-sectional survey

Journal:	*BMJ Paediatrics Open*
Manuscript ID	bmjpo-2020-001014
Article Type:	Original research
Date Submitted by the Author:	30-Dec-2020
Complete List of Authors:	Woodland, Lisa; Weston Education Centre<br>Smith, Louise; King's College London<br>Webster, Rebecca K; The University of Sheffield<br>Amlôt, Richard; Public Health England<br>Rubin, Antonia; Weald of Kent Grammar School<br>Wessely, Simon; Institute of Psychiatry Psychology and Neuroscience<br>Rubin, James
Keywords:	COVID-19

SCHOLARONE™
Manuscripts

I, the Submitting Author has the right to grant and does grant on behalf of all authors of the Work (as defined in the below author licence), an exclusive licence and/or a non-exclusive licence for contributions from authors who are: i) UK Crown employees; ii) where BMJ has agreed a CC-BY licence shall apply, and/or iii) in accordance with the terms applicable for US Federal Government officers or employees acting as part of their official duties; on a worldwide, perpetual, irrevocable, royalty-free basis to BMJ Publishing Group Ltd ("BMJ") its licensees and where the relevant Journal is co-owned by BMJ to the co-owners of the Journal, to publish the Work in this journal and any other BMJ products and to exploit all rights, as set out in our licence.

The Submitting Author accepts and understands that any supply made under these terms is made by BMJ to the Submitting Author unless you are acting as an employee on behalf of your employer or a postgraduate student of an affiliated institution which is paying any applicable article publishing charge ("APC") for Open Access articles. Where the Submitting Author wishes to make the Work available on an Open Access basis (and intends to pay the relevant APC), the terms of reuse of such Open Access shall be governed by a Creative Commons licence – details of these licences and which Creative Commons licence will apply to this Work are set out in our licence referred to above.

Other than as permitted in any relevant BMJ Author's Self Archiving Policies, I confirm this Work has not been accepted for publication elsewhere, is not being considered for publication elsewhere and does not duplicate material already published. I confirm all authors consent to publication of this Work and authorise the granting of this licence.

Confidential: For Review Only

Parents' willingness to send children back to school during the COVID-19 pandemic: a cross-sectional survey

Lisa Woodland MSc, King's College London, Weston Education Centre, Cutcombe Road, London SE5 9RJ, UK

Louise E Smith PhD, King's College London, Weston Education Centre, Cutcombe Road, London SE5 9RJ, UK

Rebecca K Webster PhD, University of Sheffield, Cathedral Court, 1 Vicar Lane, Sheffield, S1 2LT, UK

Richard Amlôt PhD, Public Health England, Emergency Response Department, Porton Down, Salisbury, SP4 0JG, UK

Antonia Rubin MA (Cantab), Trustee at Weald of Kent Grammar School, Tudeley Lane, Tonbridge, Kent, TN9 2JP, UK.

Simon Wessely PhD, King's College London, Weston Education Centre, Cutcombe Road, London SE5 9RJ, UK

G James Rubin PhD, King's College London, Weston Education Centre, Cutcombe Road, London SE5 9RJ, UK

Correspondence to: Lisa Woodland, King's College London, Weston Education Centre, Cutcombe Road, London SE5 9RJ, UK, lisa.woodland@kcl.ac.uk

Word Count: 2,623

Confidential: For Review Only

Abstract

BACKGROUND

Between 23rd of March and September 2020, schools closed to most children in England, in response to COVID-19. Parental willingness to send their children to school during this time is unclear.

METHODS

1,994 parents or guardians of school-aged children completed a cross-sectional survey as schools partially re-opened (8th and 11th June 2020). We investigated factors associated with parental willingness to send their children to school.

RESULTS

46% (n=370 / 803) of children in year-groups eligible to attend school and 13% (n=72 / 570) of children of key workers had attended school in the past seven days. The most common reasons for sending children to school were that the child's education would benefit, the child wanted to go to school, and the parent needed to work. A child was significantly more likely to attend if the parent believed the child had already had COVID-19, they had special educational needs, or a person in the household had COVID-19 symptoms. For children not eligible to attend, 38% (n=230 / 610) of participants would want to send the child to school, if it were possible. Participants were more likely to want to send their child back if the child was bored and were less likely to want to do so if they felt there was too much COVID-19 around, and that school would not be enjoyable.

CONCLUSIONS

Following any future school closure, helping parents to feel comfortable returning their child to school will require policy makers and school leaders to communicate about the adequacy of their policies to: a) ensure that the risk to children in school is minimised; b) ensure that the educational potential within schools is maximised; and c) ensure that the benefits of school for the psychological well-being of children are prioritised.

Key Words

Child; COVID-19; parents; school closures; England; cross-sectional studies

Confidential: For Review Only

Article Adds

WHAT IS KNOWN ABOUT THE SUBJECT

The COVID-19 pandemic resulted in the mass closure of schools for an extended period of time. A previous systematic review assessed parental attitudes towards smaller scale closures, finding that many parents (71%-97% across six studies) approved of closures, particularly where they were seen as an effective protective measure against a serious illness. The effect on a child's education and ability of the parent to work were reported as concerns.

WHAT THIS STUDY ADDS

During the partial reopening of schools in England, most parents did not send or want to send their children to school. Parents who were not educated to degree level, not working, who lived in the North of England or who were from Black, Asian and ethnic minority backgrounds were least likely to send their children back. Parents were more likely to want to send their child back to school if they had concerns about the impact of the closures on their child's mental well-being and education.

Confidential: For Review Only

Introduction

On 23rd March 2020, a nationwide closure of schools occurred across England in response to the COVID-19 pandemic. Schools were kept open for vulnerable children (those with a health care plan or social worker) and children of key workers.[1] From 1st June 2020 children in reception (four to five years), year one (five to six years) and year six (10 to 11 years) also became eligible to attend.[2] Until September 2020 school attendance remained voluntary.[3]

The benefits of closing schools to reduce the transmission of COVID-19 and the negative consequences of doing so are difficult to balance.[4,5] Adding to the debate was early evidence of a low transmission rate of COVID-19 among children [6-9] and a recognition that outbreaks may occur nonetheless.[10,11]

Irrespective of this debate, it is clear that many parents felt far from comfortable with their children attending school in the early stages of the pandemic, even where it was encouraged.[12] A systematic review of school closures suggested several factors that may be relevant to how comfortable a parent feels with their child attending school during an infectious disease outbreak.[13] These include perceived risk of infection,[14,15] concern about the impact of a closure on education,[14,16] and parental concerns about their child's mental health.[17] Understanding the key issues that determine a parent's willingness to send their children back to school following a closure, and ensuring that school policies and communications address these concerns, should help inform any future school closure decisions, in this or any future pandemic.

In this study, we investigated factors associated with a parent's willingness to send children to school during the COVID-19 pandemic. We investigated these factors in: parents of children in reception, year one, or year six; families where at least one parent was a key worker; and parents of school aged children who did not fall into these groups.

Methods

DESIGN

We commissioned a cross-sectional survey one week after schools in England re-opened for children in reception, year one, and year six (8th to 11th June 2020). We have previously reported data from this survey relating to parental perceptions of the presence of hygiene procedures within schools.[18]

PARTICIPANTS

Participants (n=2,447) were recruited from BMG's online research panel and were eligible for the study if they were aged eighteen years or over, lived in England, and were a parent or guardian to a school-aged child (4-18 years) who usually lived with them. 183 participants were screened out for non-eligibility, 226 participants dropped out after starting the survey and 28 completed but were removed for quality-control purposes such as completing the survey quickly or for 'straight-lining' (selecting the same option for every question). This left 2,010 participants. Demographic information was monitored by the panel provider to improve the eventual sample's

Confidential: For Review Only

representativeness (region, child age, child gender, parent/guardian age, and parent/guardian gender). The sample fell within five percentage points of the national population by the child's gender, key stage, and type of school attended against the known distribution for school children in England.[19]

Participants earned points from the research panel (equivalent to £0.60) which could be exchanged for products.

SELECTION OF A CHILD TO ANSWER QUESTIONS ABOUT

All participants were asked to select one child pseudorandomly to report on (the child with the most recent birthday). In cases where children shared a birthday, we asked the parent to select one child.

Our survey was split into two sections. Section one was only completed by parents who had a child eligible to attend school (in reception, year one, or year six and parents who did not have a child in these year groups, but they or their spouse were a key worker). Section two was completed by all parents. However, we have only included data from the second section for parents who did not complete section one.

STUDY MATERIALS

The full survey is available in the supplementary materials.

PERSONAL CHARACTERISTICS

We asked participants to report their gender, age, region, household income, employment status, marital status, ethnicity and level of education. We also asked whether anyone within the household was aged over 70 years, or had a health condition that made them vulnerable to COVID-19.

We asked participants to report the child's gender, age, school year, school type (fee-paying or state-funded) and whether the child had special educational needs (SEN).

SCHOOL ATTENDANCE LAST WEEK

Participants responding in section one were asked how many times the child had attended school in the past seven days. Depending on the response we presented parents with: ten reasons for why they were sending the child to school; twelve reasons for why they were only sending the child to school part-time; or sixteen reasons for why their child was not attending school. We asked participants to "tick any [statement] that applies". Participants also had the option to write-in text for "other reason".

WILLINGNESS TO SEND CHILD TO SCHOOL NEXT WEEK

In section two we presented all participants with nine statements about schools re-opening including "if it were possible, I would want to send [name of child] to school next week." We also presented sixteen statements about the national restrictions more generally ('lockdown'). We asked participants to respond to each on a five-point Likert-type scale from "strongly agree" to "strongly disagree".

Confidential: For Review Only

COVID-19 SYMPTOMS

We asked all participants to report if the selected child had experienced any symptoms “in the past seven days” from a list of ten symptoms. We also asked if they or a household member (other than the child) had experienced symptoms “in the past 14 days” from the same symptom list. We asked participants whether they thought their child had had COVID-19.

WELL-BEING

We asked all participants to report the child’s well-being using two subscales from the Revised Child Anxiety Disorder Scale (RCADS):[20] the generalised anxiety disorder (GAD) sub-scale and the major depressive disorder (MDD) sub-scale.

Ethics

The research was approved by the Psychiatry, Nursing and Midwifery Research Ethics Subcommittee at King’s College London (LRS—19/20-18787).

Patient and public involvement.

A school trustee contributed to the development of the survey materials and co-authored this paper.

Analysis

School attendance was defined as a child who attended school for at least one day, in the past seven days. Willingness to send a child to school next week was defined as a participant reporting “strongly agree” or “tend to agree” to this item. Variables were coded as displayed in the results.

We ran a series of binary logistic regressions using SPSS v26.0,[21] investigating univariable associations between each of our predictor variables and sending the child to school or wanting to send the child to school. We ran a second set of binary logistic regressions controlling for personal characteristics.

We analysed frequencies for the reasons given for sending an eligible child to school for a full week (five days), part-time (less than five days), and not sending the child to school.

For ease of interpretation, we used unweighted data in our analysis.

We applied a Bonferroni correction to our results ($p \leq .001$) due to running many analyses (children in school years n=29, children of key workers n=31, children non-eligible for school n=53). Results meeting this criterion are marked by a double asterisk (**) in the tables.

SAMPLE SIZE CALCULATION

During analysis, we identified some logical inconsistencies suggesting that 16 participants had accidentally completed the wrong section of the survey – we removed these participants from the analyses. Our final sample (n=1,994) was powered to give a confidence interval of approximately plus or minus 2% for each

https://mc.manuscriptcentral.com/bmjpo

Confidential: For Review Only

prevalence estimate. The three subsamples (children in eligible school years, n=803; key workers, n=570; children not eligible for school, n=621) reported in this paper all had a margin of error of plus or minus 5% at the 95% confidence level.

Results

WILLINGNESS TO SEND ELIGIBLE CHILDREN TO SCHOOL

803 participants had children in reception, year one, or year six and 570 participants reported they and or their spouse were a key worker, and that they did not have a child in eligible school years. Only n=432, 54% of children in eligible school years and n=72, 13% of children of key workers had attended school in the past seven days. One participant in each group did not know if their child had attended school. The most common reported reasons were thinking it was too risky for the child to attend school and that the school was not open (see table 1). Table 2 shows the reasons for participants to send their child to school. The most common reported reasons were the child's education would benefit and the parent needing to work. Of participants whose child attended school part-time participants commonly reported it was because the school only offered for them to be in part-time (see table 3).

Table 1: Participant reasons for children eligible for school, who had not attended school in the past week.

Reason	Children in School Years (n=432, %)	Children of Key Workers (n=497, %)
I think it is too risky for my child to attend school at the moment	223 (52%)	109 (22%)
The school is not open	140 (32%)	259 (52%)
I have another child who can't go to school	67 (15·5%)	24 (5%)
Being at school will be stressful for my child	59 (14%)	23 (5%)
I think they will get a better education at home than at school at the moment	57 (13%)	25 (5%)
The school has asked my child not to attend	51 (12%)	117 (23·5%)
Someone in my household is clinically vulnerable to coronavirus	45 (10%)	24 (5%)
My child doesn't want to go	44 (10%)	29 (6%)
There's no point, schools will shut again soon anyway	37 (9%)	13 (3%)
My child's friends will not be at school	28 (6·5%)	19 (4%)
Other reason	28 (6·5%)	65 (13%)
I think friends, family or other parents will judge me if I send my child to school	12 (3%)	4 (1%)
I can't arrange transport to get them to school	6 (1%)	10 (2%)
Someone else at the school developed coronavirus symptoms (cough or fever, or change in sense of taste or smell)	6 (1%)	4 (1%)
My child developed symptoms of a different illness	3 (1%)	1 (0%)
They have developed coronavirus symptoms (cough or fever, or change in sense of taste or smell)	2 (0·5%)	2 (0%)
Someone else in the family developed coronavirus symptoms (cough or fever, or change in sense of taste or smell)	0 (0%)	3 (1%)

Table 2: Participant reasons for children eligible for school, who had attended school at least one day in the past week.

Reason	Children in School Years (n=370, %)	Children of Key Workers (n=72, %)

Confidential: For Review Only

My child's education will benefit from being at school	208 (56%)	30 (42%)
My child wants to go to school	200 (54%)	35 (49%)
My child will benefit from seeing their friends	187 (50·5%)	27 (37·5%)
I need to work	120 (32%)	40 (56%)
I don't believe my child will be at risk at school	96 (26%)	17 (24%)
Having my child at home is a strain	57 (15%)	10 (14%)
It is compulsory for my child to attend	50 (13.5%)	9 (12.5%)
It reduces costs at home	48 (13%)	7 (10%)
I think friends, family or other parents will judge me if I don't send my child to school	43 (12%)	11 (15%)
I have non-work commitments I need to meet	21 (6%)	4 (6%)
Other	9 (2%)	3 (4%)

Table 3: Participant reasons for children eligible for school, who attended school part-time.

**Reason**	**Children in School Years (n=204, %)**	**Children of Key Workers (n=39, %)**
The school only offers for them to be in part-time	80 (39%)	16 (41%)
It is less risky for them to be in part-time	40 (20%)	7 (18%)
I am only sending them in on days where the lessons are important	28 (14%)	8 (20·5%)
Other reason	27 (13%)	3 (8%)
I am using it as childcare and I only need them in part time	24 (12%)	6 (15%)
I am only sending them in on days when I can arrange transport	23 (11%)	4 (10%)
Someone else at the school developed coronavirus symptoms (cough or fever, or change in sense of taste or smell)	14 (7%)	1 (3%)
I started sending them in, but have now changed my mind	13 (6%)	2 (5%)
They have developed coronavirus symptoms (cough or fever, or change in sense of taste or smell)	13 (6%)	1 (3%)
Someone else in the family developed coronavirus symptoms (cough or fever, or change in sense of taste or smell)	12 (6%)	0 (0%)
I am only sending them in on days when their friends are in	11 (5%)	6 (15%)
My child developed symptoms of a different illness	9 (4%)	0 (0%)
They only live with me part time	6 (3%)	1 (3%)

Confidential: For Review Only

Participant characteristics for children in eligible school years and for children of key workers are shown in table 4. Parents of children in eligible school years were less likely to send the child to school if they were educated to A-level or below, not working, of Black, Asian and minority ethnicity (BAME), or living in the North East, North West, and Yorkshire and the Humber compared to London. Whereas children of key workers were more likely to attend school for participants aged 45 and under and who were working. Child attendance was more likely for children in eligible school years when in a fee-paying school and if they had a health condition that made them vulnerable to COVID-19. For both groups' attendance was more likely for children who had special educational needs, reported low well-being, thought their child had had COVID-19 and when a person over 70 was living in the household. There was also a significant association in both groups between a child being more likely to attend school and the child having experienced COVID-19 symptoms in the

past 7 days, or another person in the household having experienced COVID-19 symptoms in the past 14 days.

Confidential: For Review Only

Table 4. Participant characteristics for children in eligible school years (n=803) and children of key works (n=570), by school attendance.

		**Children in Eligible school years**				**Children of key workers**			
	Level	Did not attend school, n (%)	Attended School, n (%)	Odds ratio (95% CI)	Adjusted odds ratio (95% CI) †	Did not attend school, n (%)	Attended School, n (%)	Odds ratio (95% CI)	Adjusted odds ratio (95% CI) †
Gender	Male	201 (52)	187 (48)	1·22 (0·92 to 1·61)	1·04 (0·75 to 1·43)	227 (85)	39 (15)	1·53 (0·92 to 2·55)	1·61 (0·91 to 2·85)
	Female	230 (57)	177 (43)	Reference	Reference	268 (90)	30 (10)	Reference	Reference
Age	18-35 years	129 (49)	136 (51)	1·37 (0·92 to 2·03)	1·23 (0·79 to 1·92)	54 (77)	16 (23)	**3·16* (1·56 to 6·37)**	**2·95* (1·23 to 7·08)**
	36-45 years	212 (56)	164 (44)	1·01 (0·70 to 1·46)	0·98 (0·65 to 1·48)	198 (86)	33 (14)	**1·76* (1·01 to 3·12)**	**2·14* (1·12 to 4·09)**
	46 years ≥	91 (56·5)	70 (43·5)	Reference	Reference	245 (91)	23 (9)	Reference	Reference
Region	East Midlands	33 (60)	22 (40)	**0·50* (0·27 to 0·93)**	0·59 (0·31 to 1·14)	45 (85)	8 (15)	0·85 (0·32 to 2·22)	1·23 (0·40 to 3·75)
	East of England	38 (48)	41 (52)	0·81 (0·48 to 1·38)	0·92 (0·52 to 1·64)	54 (82)	12 (18)	1·06 (0·45 to 2·52)	1·38 (0·50 to 3·81)
	North East	33 (67)	16 (33)	**0·36* (0·19 to 0·71)**	**0·38* (0·81 to 0·78)**	32 (89)	4 (11)	0·60 (0·18 to 1·98)	0·93 (0·25 to 3·46)
	North West	68 (65)	36 (35)	**0·40** (0·24 to 0·66)**	**0·39* (0·23 to 0·68)**	80 (93)	6 (7)	**0·36* (0·13 to 0·99)**	0·61 (0·19 to 1·89)
	South East	70 (51)	66 (49)	0·71 (0·45 to 1·11)	0·92 (0·56 to 1·52)	82 (89)	10 (11)	0·58 (0·24 to 1·94)	0·81 (0·29 to 2·29)
	South West	24 (46)	28 (54)	0·88 (0·47 to 1·63)	1·16 (0·58 to 2·31)	42 (87·5)	6 (12·5)	0·68 (0·42 to 1·94)	1·18 (0·37 to 3·82)
	West Midlands	39 (56)	30 (44)	0·58 (0·33 to 1·01)	0·63 (0·34 to 1·18)	48 (89)	6 (11)	0·60 (0·21 to 1·68)	0·84 (0·27 to 2·60)
	Yorkshire & the Humber	52 (62)	31 (37)	**0·45* (0·26 to 0·76)**	**0·52* (0·29 to 0·93)**	52 (88)	7 (12)	0·64 (0·24 to 1·73)	0·80 (0·27 to 2·41)
	London	75 (43)	100 (57)	Reference	Reference	62 (83)	13 (17)	Reference	Reference
Household income	≤ £34,999	164 (54)	142 (46	1·00 (0·75 to 1·33)	1·34 (0·95 to 1·90)	185 (89)	23 (11)	0·75 (0·44 to 1·28)	0·96 (0·51 to 1·81)
	£35,000 ≥	254 (54)	220 (46)	Reference	Reference	285 (86)	47 (14)	Reference	Reference
Employment status[1]	Working	362 (52)	337 (48)	**1·95* (1·25 to 3·05)**	**1·94* (1·16 to 3·24)**	435 (86)	70 (14)	**9·33* (1·27 to 68·47)**	**8·64* (1·13 to 65·96)**
	Not working	67 (68)	32 (32)	Reference	Reference	58 (98)	1 (2)	Reference	Reference
Education level	≤ A-level	179 (63)	105 (37)	**0·56** (0·41 to 0·75)**	**0·59* (0·42 to 0·84)**	227 (88)	31 (12)	0·91 (0·55 to 1·51)	1·27 (0·71 to 2·26)
	Degree ≥	248 (49)	261 (51)	Reference	Reference	267 (87)	40 (13)	Reference	Reference
Marital status	Living alone	49 (48)	54 (52)	1·34 (0·88 to 2·02)	1·30 (0·82 to 2·06)	75 (88)	10 (12)	0·91 (0·45 to 1·85)	0·92 (0·41 to 2·03)
	Married/cohabiting	383 (55)	316 (45)	Reference	Reference	422 (87)	62 (13)	Reference	Reference
Ethnicity	White	355 (53)	316 (47)	1·23 (0·84 to 1·12)	**1·66* (1·07 to 2·58)**	450 (87)	65 (13)	1·04 (0·42 to 2·53)	1·47 (0·53 to 4·13)
	BAME	72 (58)	52 (42)	Reference	Reference	43 (88)	12 (22)	Reference	Reference
Child gender	Boy	230 (51·5)	217 (48·5)	1·25 (0·94 to 1·65)	1·15 (0·85 to 1·56)	244 (87)	36 (13)	1·04 (0·63 to 1·70)	0·96 (0·56 to 1·66)
	Girl	202 (57)	153 (43)	Reference	Reference	253 (87·5)	36 (12·5)	Reference	Reference
School year	Early Years	77 (59)	53 (41)	0·74 (0·50 to 1·10)	0·79 (0·51 to 1·23)	***	***	***	***
	Key Stage 1	157 (54)	132 (46)	0·90 (0·66 to 1·22)	0·88 (0·63 to 1·24)	30 (86)	5 (14)	2·10 (0·57 to 7·81)	1·52 (0·37 to 6·30)
	Key Stage 2	198 (52)	185 (48)	Reference	Reference	120 (86)	20 (15)	2·10 (0·75 to 5·86)	1·45 (0·47 to 4·44)
	Key Stage 3	***	***	***	***	152 (87)	23 (13)	1·91 (0·69 to 5·24)	1·58 (0·55 to 4·55)
	Key Stage 4	***	***	***	***	132 (87)	19 (13)	1·81 (0·65 to 5·08)	1·44 (0·49 to 4·24)
	Years 12 & 13	***	***	***	***	63 (93)	5 (7)	Reference	Reference
School type	Fee-paying	35 (33)	71 (67)	**2·68** (1·74 to 4·13)**	**2·50** (1·56 to 4·00)**	30 (77)	9 (23)	**2·20* (1·00 to 4·85)**	2·04 (0·82 to 5·07)
	State-funded	395 (57)	299 (43)	Reference	Reference	462 (88)	63 (12)	Reference	Reference
Child has SEN	Yes	22 (31)	48 (69)	**2·78** (1·64 to 4·70)**	**2·30* (1·27 to 4·17)**	28 (61)	18 (39)	**5·74** (2·97 to 11·08)**	**7·92** (3·59 to 17·46)**
	No	406 (56)	319 (44)	Reference	Reference	464 (90)	52 (10)	Reference	Reference
Low child well-being	Yes	92 (42)	124 (57)	**1·86** (1·36 to 2·55)**	**1·47* (1·04 to 2·07)**	127 (82)	28 (18)	**1·85* (1·11 to 3·10)**	**1·78* (1·00 to 3·21)**
	No	340 (58)	246 (42)	Reference	Reference	370 (89)	44 (11)	Reference	Reference

Confidential: For Review Only

Child vulnerable COVID-19	Yes	28 (39)	44 (61)	**2·00* (1·21 to 3·28)**	**1·76* (1·00 to 3·10)**	33 (82·5)	7 (17·5)	1·49 (0·63 to 3·50)	0·93 (0·33 to 2·63)
	No	401 (56)	316 (44)	Reference	Reference	455 (87·5)	65 (12·5)	Reference	Reference
Household vulnerable COVID-19	Yes	115 (60)	78 (40)	0·78 (0·56 to 1·08)	0·74 (0·51 to 1·06)	134 (88)	18 (12)	0·90 (0·50 to 1·60)	0·56 (0·44 to 1·67)
	No	295 (53)	258 (47)	Reference	Reference	314 (87)	47 (13)	Reference	Reference
Child COVID-19 symptoms	Yes	15 (24)	47 (76)	**5·05** (2·22 to 7·36)**	**3·02* (1·49 to 6·15)**	11 (58)	8 (42)	**5·52** (2·14 to 14·24)**	**5·25* (1·58 to 17·41)**
	No	417 (56)	323 (44)	Reference	Reference	486 (88)	64 (12)	Reference	Reference
Household COVID-19 symptoms	Yes	9 (17)	43 (83)	**6·18** (2·97 to 12·86)**	**6·04** (2·62 to 13·91)**	13 (65)	7 (35)	**4·00* (1·54 to 10·42)**	**4·11* (1·26 to 13·44)**
	No	423 (56)	327 (43)	Reference	Reference	484 (88)	65 (12)	Reference	Reference
Child has had or currently has COVID-19	Yes	36 (24)	112 (76)	**4·78** (3·18 to 7·17)**	**4·03** (2·57 to 6·30)**	40 (67)	20 (33)	**4·40** (2·40 to 8·08)**	**5·20** (2·48 to 10·93)**
	No	396 (61)	258 (37)	Reference	Reference	457 (90)	52 (10)	Reference	Reference
Someone over 70 years	Yes	11 (26)	32 (74)	**3·62** (1·80 to 7·30)**	**3·19* (1·45 to 7·05)**	8 (57)	6 (43)	**5·56* (1·87 to 16·52)**	**3·49* (0·98 to 12·46)**
	No	421 (55·5)	338 (44·5)	Reference	Reference	489 (88)	66 (12)	Reference	Reference

* *p≤ ·05* and formatted bold

** *p≤ ·001* and formatted bold

*** data not applicable for the sample

† Adjusting for participant gender, age, region, household income, employment status, education level, marital status, participant ethnicity, and the child's gender, school year and school-type.

[1] Working includes students and volunteers

Decimal places have been used for rounding errors

Confidential: For Review Only

Confidential: For Review Only

WILLINGNESS TO SEND NON-ELIGIBLE CHILDREN TO SCHOOL NEXT WEEK

Of the remaining 621 participants, most reported not wanting to send their child to school (n=380, 62%), characteristics are shown in table 5. Participants educated at degree level, had children in Key Stage 2 and who thought their child had already had COVID-19 were more likely to send their child to school. This was also true if the child had been bored, and if the participant had found it hard to keep up with work or other important commitments (see table 6). There was a strong association between feeling comfortable in sending the child to school next week and wanting to send the child to school. Participants were less likely to want to send their child to school if they felt: there was still too much COVID-19 around for schools to be able to re-open safely; child was worried about coronavirus; that when schools fully re-open, some parents will send their children to school even if they have symptoms of coronavirus; children would not wash their hands properly at school; schools will not be able to provide good quality education at the moment; and schools will not be an enjoyable place for the child to be at the moment.

Table 5. Characteristics for participants when they or their spouse are not a key worker and do not have a child in reception, year one and year six (n=621), by want to send to school next week.

	Level	Do not want to send to school, n (%)	Want to send to school, n (%)	Odds ratio (95% CI)	Adjusted odds ratio (95% CI) †
Gender	Male	147 (56)	116 (44)	**1·61* (1·15 to 2·24)**	**1·47* (1·00 to 2·17)**
	Female	232 (67)	114 (33)	Reference	Reference
Age	18-35 years	41 (74·5)	14 (25·5)	0·54 (0·28 to 1·03)	0·73 (0·35 to 1·54)
	36-45 years	151 (61)	97 (39)	1·02 (0·72 to 1·43)	1·10 (0·73 to 1·66)
	46 years ≥	188 (61)	119 (39)	Reference	Reference
Region	East Midlands	27 (63)	16 (37)	0·70 (0·33 to 1·46)	0·77 (0·33 to 1·77)
	East of England	41 (58)	30 (42)	0·86 (0·46 to 1·61)	0·90 (0·45 to 1·82)
	North East	21 (68)	10 (32)	0·56 (0·24 to 1·32)	0·60 (0·23 to 1·57)
	North West	54 (67·5)	26 (32·5)	0·56 (0·30 to 1·06)	0·74 (0·37 to 1·48)
	South East	68 (66)	35 (34)	0·60 (0·34 to 1·08)	0·65 (0·34 to 1·24)
	South West	37 (62)	23 (38)	0·73 (0·37 to 1·42)	0·83 (0·40 to 1·75)
	West Midlands	54 (65)	29 (35)	0·63 (0·34 to 1·16)	0·61 (0·30 to 1·21)
	Yorkshire & the Humber	30 (60)	20 (40)	0·78 (0·39 to 1·58)	1·25 (0·57 to 2·77)
	London	48 (54)	41 (46)	Reference	Reference
Household income	≤ £34,999	188 (68)	90 (32)	**0·61* (0·43 to 0·86)**	0·89 (0·59 to 1·33)
	£35,000 ≥	163 (56)	128 (44)	Reference	Reference
Employment status[1]	Working	271 (60)	183 (40)	**1·57* (1·06 to 2·32)**	1·50 (0·94 to 2·39)
	Not working	107 (70)	46 (30)	Reference	Reference
Education level	≤ A-level	204 (68)	95 (32)	**0·61* (0·44 to 0·85)**	**0·62* (0·42 to 0·91)**
	Degree ≥	175 (57)	133 (43)	Reference	Reference
Marital Status	Living alone	96 (70)	41 (30)	**0·64* (0·43 to 0·97)**	0·69 (0·43 to 1·09)
	Married/cohabiting	284 (60)	189 (40)	Reference	Reference
Ethnicity	White	334 (62)	208 (38)	1·37 (0·79 to 2·39)	1·72 (0·90 to 3·29)
	BAME	44 (69)	20 (31)	Reference	Reference
Child gender	Boy	195 (61)	195 (39)	1·15 (0·83 to 1·60)	1·25 (0·87 to 1·79)
	Girl	185 (64)	104 (36)	Reference	Reference
Child school year	Key Stage 1	36 (69)	16 (31)	0·91 (0·43 to 1·91)	1·14 (0·50 to 2·62)
	Key Stage 2	72 (52)	67 (48)	**1·90* (1·07 to 3·35)**	**2·77* (1·44 to 5·35)**
	Key Stage 3	123 (67)	60 (33)	0·99 (0·57 to 1·73)	1·23 (0·66 to 2·28)
	Key Stage 4	94 (61)	60 (39)	1·30 (0·74 to 2·28)	1·60 (0·86 to 2·96)
	Years 12 & 13	55 (67)	27 (33)	Reference	Reference

Child school type	Fee-paying	27 (64)	15 (36)	0·90 (0·47 to 1·72)	1·07 (0·50 to 2·30)
	State-funded	347 (62)	215 (38)	Reference	Reference
Child has SEN	Yes	28 (72)	11 (28)	0·63 (0·31 to 1·28)	0·55 (0·24 to 1·26)
	No	349 (61)	219 (39)	Reference	Reference
Low child well-being	Yes	94 (64)	53 (36)	0·91 (0·62 to 1·34)	0·83 (0·54 to 1·27)
	No	286 (62)	177 (38)	Reference	Reference
Child vulnerable COVID-19	Yes	29 (71)	12 (29)	0·66 (0·33 to 1·32)	0·56 (0·26 to 1·23)
	No	347 (61·5)	217 (38·5)	Reference	Reference
Household vulnerable COVID-19	Yes	106 (69)	48 (31)	**0·64* (0·43 to 0·95)**	0·75 (0·49 to 1·16)
	No	222 (58)	158 (42)	Reference	Reference
Child has had or currently has COVID-19	Yes	6 (40)	24 (60)	**2·65* (1·38 to 5·10)**	**2·82* (1·35 to 5·91)**
	No	364 (64)	206 (36)	Reference	Reference
Someone over 70 years	Yes	22 (73)	8 (27)	0·57 (0·26 to 1·34)	0·58 (0·23 to 1·46)
	No	358 (62)	358 (38)	Reference	Reference

* *p≤ ·05* and formatted bold

† Adjusting for participant gender, age, region, household income, employment status, education level, marital status, participant ethnicity, and the child's gender, school year and school-type.

[1] Working includes students and volunteers

Decimal places have been used for rounding errors

Table 6: Participant perceptions for children not eligible for school, by want to send to school.

	Level	Do not want to send to school (n, %)	Want to send to school (n, %)	Odds ratio (95%, CI)	Adjusted odds ratio (95%, CI) †
If child goes out, she/he is likely to catch coronavirus	Agree	56 (66%)	29 (34%)	0·82 (0·51 to 1·32)	0·81 (0·47 to 1·42)
	Disagree	316 (61%)	200 (39%)	Reference	Reference
If child goes out, she/he is likely to bring coronavirus back into our home	Agree	58 (60%)	38 (40%)	1·07 (0·69 to 1·67)	1·14 (0·68 to 1·90)
	Disagree	312 (62%)	191 (38%)	Reference	Reference
Child is keeping up with his/her schoolwork	Agree	269 (63%)	161 (37%)	0·98 (0·68 to 1·42)	0·88 (0·58 to 1·32)
	Disagree	103 (62%)	63 (38%)	Reference	Reference
I feel confident helping child with her/his schoolwork	Agree	234 (59%)	164 (41%)	**1·56* (1·09 to 2·2·4)**	1·28 (0·86 to 1·90)
	Disagree	138 (69%)	62 (31%)	Reference	Reference
I feel supported by child's school	Agree	221 (61%)	143 (39%)	1·15 (0·82 to 1·60)	1·22 (0·84 to 1·77)
	Disagree	154 (64%)	87 (36%)	Reference	Reference
I have access to all the resources that child needs to do her/his schoolwork	Agree	260 (60%)	172 (40%)	1·40 (0·96 to 2·04)	1·33 (0·87 to 2·02)
	Disagree	112 (68%)	53 (32%)	Reference	Reference
During lockdown, child has learned about important things she/he wouldn't normally learn at school	Agree	194 (61%)	124 (39%)	1·10 (0·79 to 1·53)	1·05 (0·73 to 1·51)
	Disagree	183 (63%)	106 (37%)	Reference	Reference
In the past 7 days, child has been bored	Agree	214 (56·5%)	165 (43·5%)	**1·97** (1·39 to 2·80)**	**2·05** (1·38 to 3·05)**
	Disagree	166 (72%)	65 (28%)	Reference	Reference
In the past 7 days, my household has had a regular structure to the day	Agree	249 (61%)	229 (39%)	1·16 (0·82 to 1·65)	0·97 (0·66 to 1·43)
	Disagree	130 (65%)	71 (35%)	Reference	Reference
In the past 7 days, child has kept in touch with her/his friends	Agree	275 (61%)	179 (39%)	1·30 (0·89 to 1·91)	1·22 (0·79 to 1·92)
	Disagree	102 (67%)	51 (33%)	Reference	Reference
Child is worried about coronavirus	Agree	179 (67%)	87 (33%)	**0·67* (0·48 to 0·94)**	**0·69* (0·47 to 0·99)**
	Disagree	198 (58%)	143 (42%)	Reference	Reference
In the past 7 days, child has felt upset about not seeing other family members who do not live with us	Agree	138 (59%)	96 (41%)	1·29 (0·92 to 1·81)	1·41 (0·97 to 2·07)
	Disagree	239 (65%)	129 (35%)	Reference	Reference
In the past 7 days, I have found it hard to keep up with work or other important commitments	Agree	76 (51%)	73 (49%)	**1·77* (1·21 to 2·58)**	**1·85* (1·19 to 2·88)**
	Disagree	276 (65%)	150 (35%)	Reference	Reference
In the past 7 days, people in my household have been getting along well	Agree	301 (63·5%)	173 (36·5%)	0·81 (0·54 to 1·19)	0·80 (0·52 to 1·25)

Confidential: For Review Only

	Disagree	58% (77)	55 (42%)	Reference	Reference
I am worried about the financial impact of lockdown measures	Agree	202 (59%)	141 (41%)	1·37 (0·98 to 1·92)	1·28 (0·88 to 1·85)
	Disagree	173 (66%)	88 (34%)	Reference	Reference
Before the school closures, child had extra support at school	Agree	72 (58%)	53 (42%)	1·29 (0·86 to 1·94)	1·26 (0·80 to 1·98)
	Disagree	261 (64%)	149 (36%)	Reference	Reference
If it were possible, I would feel comfortable sending child to school next week	Agree	46 (19%)	194 (89%)	**38·89** (24·29 to 62·28)**	**41·92** (24·52 to 71·67)**
	Disagree	332 (90%)	36 (10%)	Reference	Reference
There is still too much coronavirus around for schools to be able to reopen safely	Agree	290 (80%)	74 (20%)	**0·15** (0·10 to 0·21)**	**0·14** (0·09 to 0·21)**
	Disagree	90 (37%)	156 (63%)	Reference	Reference
When child's school fully reopens, some parents will send their children to school, even if they have symptoms of coronavirus	Agree	191 (67%)	93 (33%)	**0·66* (0·47 to 0·92)**	**0·59* (0·51 to 0·85)**
	Disagree	185 (57·5%)	137 (42·5%)	Reference	Reference
It is impossible for children to maintain 'social distancing' at school	Agree	307 (64%)	175 (36%)	0·75 (0·50 to 1·11)	0·71 (0·46 to 1·10)
	Disagree	72 (57%)	55 (45%)	Reference	Reference
Children will not wash their hands properly at school	Agree	239 (67%)	120 (33%)	**0·63* (0·45 to 0·88)**	**0·58* (0·40 to 0·84)**
	Disagree	138 (56%)	110 (44%)	Reference	Reference
Staff will not wash their hands properly at school	Agree	56 (56%)	44 (44%)	1·35 (0·88 to 2·09)	1·45 (0·88 to 2·38)
	Disagree	320 (63%)	186 (37%)	Reference	Reference
Schools will not be able to provide good quality education at the moment	Agree	169 (66·5%)	85 (33·5%)	0·73 (0·52 to 1·02)	**0·65* (0·45 to 0·95)**
	Disagree	208 (59%)	144 (41%)	Reference	Reference
School will not be an enjoyable place for child to be at the moment	Agree	237 (76·5%)	73 (23·5%)	**0·28** (0·20 to 0·40)**	**0·28** (0·19 to 0·40)**
	Disagree	142 (48%)	155 (52%)	Reference	Reference

* *p≤ ·05* and formatted bold
** *p≤ ·001* and formatted bold
† Adjusting for participant gender, age, region, household income, employment status, education level, marital status, participant ethnicity, and the child's gender, school year and school-type.
Decimal places have been used for rounding errors

Discussion

Most children eligible to attend school did not attend school. Among parents whose children were not eligible to attend, most did not want to send their children to school. Worryingly, we observed patterns that seemed likely to entrench existing educational inequalities, with children from households where parents have lower education achievements, BAME households and households in the North of England being least likely to attend, while children at fee-paying schools were more likely to attend.

Our results suggest that several broad areas determined parental willingness for their child to return to school. First, risk perceptions were crucial. The response "it is too risky" featured highly in parental reasons for not sending children to school, and parents were less likely to want to send the child to school if they felt there "was too much COVID-19 around for schools to open safely." Secondly, parental attitudes reflected perceived risk of transmission of the virus. Parents who worried that children in school would not wash their hands properly and that other parents would send sick children to school were less willing to send their child to school. Children were also more likely to attend school when parents thought their child had had COVID-19 which could indicate the belief their child is immune to further infection.[22] Unexpectedly, children were more likely to attend school when they had a health condition that made them vulnerable to COVID-19 and when a person

Confidential: For Review Only

over 70 years was living in the household. We speculate that this may be because these circumstances affect the parent's ability to look after the child at home.

Poorer perceived child well-being was associated with them being more likely to attend school. This finding may reflect parental desire to improve their child's well-being which may have been impacted by the school closure.[23] This was apparent in reasons parents gave for sending their child to school. It also corresponds with our finding that a child was more likely to attend school if they were bored at home. In contrast, parents were less likely to want to send their child to school when they felt that school would not be enjoyable for the child, and that the child was worried about COVID-19. As expected,[13] concerns about education also featured highly as a reason for attendance, while perceptions that schools could not provide good quality education or that some lessons were not as important were cited as reasons for absence or partial attendance.

Despite most children of key workers not attending school, needing to work was the most reported reason for school attendance and the school not opening or asking the child not to attend were the most commonly reported reasons for the child not being in school. This suggests that work commitments were the main driver for this group.

One troubling finding was that children who had symptoms of COVID-19 in the past seven days, or whose household members had these symptoms in the past 14 days were significantly more likely to have gone to school. We do not know if the child attended school whilst having symptoms or when symptoms were present in the household (against self-isolation guidance)[24] although amongst adults full adherence to self-isolation guidelines is known to be poor.[25] However, given that school closure is specifically designed to reduce the transmission of respiratory infections in general, it is also possible that this reflects the re-emergence of transmission of upper respiratory tract infections, or COVID-19 specifically, within the school environment.[26] It may also reflect increased anxiety or awareness among parents around COVID-19 symptoms, resulting in higher symptom detection and reporting.

LIMITATIONS

Several limitations should be borne in mind for this study. First, we ran many analyses raising the possibility of Type 1 errors. While we have provided Bonferroni corrections in the tables for readers who wish to correct for this, this correction in turn may be overly conservative. Second, we used purposive sampling to broadly represent parents and children in England. Online polls can be unrepresentative, and lead to response, and self-reporting bias.[27] However, in line with the reasoning relating to the use of non-probability samples in the social sciences,[28] we assume that the associations within our data do generalise to the wider population. Fourth, the cross-sectional nature of this study limits our ability to draw casual findings. Fifth, the RCADS sub-scale was used to indicate low well-being but is currently not validated for children under eight years.[29]

CONCLUSION

Confidential: For Review Only

Parents were primarily concerned about the risk of COVID-19. However, they were more likely to want to send their child back to school if they had concerns about the impact of the closures on their child's mental well-being and education. Parents will feel more comfortable in sending their children to school if reassured that school will be safe, educational and enjoyable.

Contributors

All authors contributed to the conceptualisation of the study and approved the final draft. LW designed the survey, analysed the data and drafted the manuscript. LES designed the survey, analysed the data and edited the manuscript. RKW edited the manuscript. RA designed the survey and edited the manuscript. AR designed the survey and edited the manuscript. SW edited the manuscript. GJR designed the survey, analysed the data and edited the manuscript.

Declarations of interests

We declare no competing interests.

Acknowledgments

This study was funded by the Economic and Social Research Council and the National Institute for Health Research Health Protection Research Unit (NIHR HPRU) [grant number NIHR200890] in Emergency Preparedness and Response, a partnership between Public Health England, King's College London and the University of East Anglia. The views expressed are those of the authors and not necessarily those of the NIHR, Public Health England or the Department of Health and Social Care.

Role of the funding source

The funders had no role in study design, data collection, data analysis, data interpretation, or writing of the manuscript. The corresponding author had full access to all the data and had final responsibility for the decision to submit for publication.

Confidential: For Review Only

References

1 Prime Minister B Johnson. PM statement on coronavirus: 18 March 2020. Available at: https://www.gov.uk/government/speeches/pm-statement-on-coronavirus-18-march-2020?utm_source=407969fc-4de1-41c8-9571-f6b6ad35efae&utm_medium=email&utm_campaign=govuk-notifications&utm_content=immediate Accessed 11 June 2020.
2 Prime Minister B Johnson. PM confirms schools, colleges and nurseries on track to begin phased reopening. Available at: https://www.gov.uk/government/news/pm-confirms-schools-colleges-and-nurseries-on-track-to-begin-phased-reopening Accessed 11 June 2020.
3 HM Government. School Attendance. Available at: https://www.gov.uk/government/publications/school-attendance Accessed 8 July 2020.
4 Viner RM, Russell SJ, Croker H, et al. School closure and management practices during coronavirus outbreaks including COVID-19: a rapid systematic review. *The Lancet Child & Adolescent Health*. 2020;4(5):397-404 DOI: 10.1016/s2352-4642(20)30095-x
5 Keogh-Brown MR, Jensen HT, Edmunds WJ, Smith RD. The impact of Covid-19, associated behaviours and policies on the UK economy: A computable general equilibrium model. *SSM - Population Health*. 2020;[in press]:100651 DOI: 10.1016/j.ssmph.2020.100651
6 Boast A, Munro A, Goldstein H. An evidence summary of Paediatric COVID-19 literature. Available at: https://dontforgetthebubbles.com/evidence-summary-paediatric-covid-19-literature/ Accessed 8 July 2020.
7 Zhu Y, Bloxham C, K H, et al. Children are unlikley to have been the primary source of household SARS-CoV-2 infections. 2020; DOI: 10.1101/2020.03.26.20044826v1
8 World Health Organization. Report of the WHO-China joint mission on coronavirus disease 2019 (COVID-19). Available at: https://www.who.int/publications/i/item/report-of-the-who-china-joint-mission-on-coronavirus-disease-2019-(covid-19) Accessed 8 July 2020.
9 Heavey L, Casey G, Kelly C, et al. No evidence of secondary transmission of COVID-19 from children attending school in Ireland, 2020. *Eurosurveillance*. 2020;25(21) DOI: 10.2807/1560-7917.es.2020.25.21.2000903
10 Otte Im Kampe E, Lehfeld A-S, Buda S, et al. Surveillance of COVID-19 school outbreaks, Germany, March to August 2020. *Eurosurveillance*. 2020;25(38) DOI: 10.2807/1560-7917.es.2020.25.38.2001645
11 England. PH. SARS-CoV-2 infection and transmission in educational settings. Available at: https://www.gov.uk/government/publications/sars-cov-2-infection-and-transmission-in-educational-settings Accessed 22 October 2020.
12 Royal College of Paediatrics and Child Health. COVID_19 - talking to children and families about returning to school: guiding principles. Available at: https://www.rcpch.ac.uk/resources/covid-19-talking-children-families-about-returning-school-guiding-principles Accessed 9 July 2020.
13 Brooks SK, Smith LE, Webster RK, et al. The impact of unplanned school closure on children's social contact: rapid evidence review. *Euro Surveill*. 2020;25(13) DOI: 10.2807/1560-7917.ES.2020.25.13.2000188
14 Basurto-Dávila R, Garza R, Meltzer MI, et al. Household economic impact and attitudes toward school closures in two cities in Argentina during the 2009 influenza

Confidential: For Review Only

A (H1N1) pandemic. *Influenza and Other Respiratory Viruses*. 2013;7(6):1308-15 DOI: 10.1111/irv.12054
15 Effler PV, Carcione D, Giele C, et al. Household responses to pandemic (H1N1) 2009-related school closures, Perth, Western Australia. *Emerg Infect Dis*. 2010;16(2):205-11 DOI: 10.3201/eid1602.091372
16 van Gemert C, McBryde ES, Bergeri I, et al. Description of social contacts among student cases of pandemic influenza during the containment phase, Melbourne, Australia, 2009. *Western Pac Surveill Response J*. 2018;9(5 Suppl 1):27-34 DOI: 10.5365/wpsar.2018.9.5.003
17 Lee J. Mental health effects of school closures during COVID-19. *The Lancet Child & Adolescent Health*. 2020;4(6):421 DOI: 10.1016/s2352-4642(20)30109-7
18 Smith LE, Woodland L, Amlôt R, et al. A cross-sectional survey of parental perceptions of COVID-19 related hygiene measures within schools and adherence to social distancing in journeys to and from school. *BMJ Paediatrics Open*. 2020;4(1) DOI: 10.1136/bmjpo-2020-000825
19 HM Government. Schools, pupils and their characteristics: Academic Year 2019/20. Available at: https://explore-education-statistics.service.gov.uk/find-statistics/school-pupils-and-their-characteristics Accessed 25 June 2020.
20 Chorpita BF, Yim L, Moffitt C, et al. Assessment of symptoms of DSM-IV anxiety and depression in children: a revised child anxiety and depression scale. *Behaviour Research and Therapy*. 2000;38(8):835-55 DOI: 10.1016/s0005-7967(99)00130-8
21 IBM Corp. IBM SPSS Statistics for Windows. 26.0 2019
22 Smith LE, Mottershaw AL, Egan M, et al. The impact of believing you have had COVID-19 on self-reported behaviour: Cross-sectional survey. *PLOS ONE*. 2020;15(11):e0240399 DOI: 10.1371/journal.pone.0240399
23 Idoiaga M, Berasategi S, Dosil S, Eiguren M. Struggling to breathe: a qualitative study of children's wellbeing during lockdown in Spain. *Psychology & Health*. 2020:1-16 DOI: 10.1080/08870446.2020.1804570
24 HM Government. Protective measures for out-of-school settings during the coronavirus (COVID-19) outbreak. Available at: https://www.gov.uk/government/publications/protective-measures-for-holiday-or-after-school-clubs-and-other-out-of-school-settings-for-children-during-the-coronavirus-covid-19-outbreak/protective-measures-for-out-of-school-settings-during-the-coronavirus-covid-19-outbreak Accessed 8 July 2020.
25 Smith LE, Amlôt R, Lambert H, et al. Factors associated with adherence to self-isolation and lockdown measures in the UK: a cross-sectional survey. *Public Health*. 2020;187:41-52 DOI: 10.1016/j.puhe.2020.07.024
26 Panovska-Griffiths J, Kerr CC, Stuart RM, et al. Determining the optimal strategy for reopening schools, the impact of test and trace interventions, and the risk of occurrence of a second COVID-19 epidemic wave in the UK: a modelling study. *The Lancet Child & Adolescent Health*. 2020; DOI: 10.1016/s2352-4642(20)30250-9
27 Epstein WM. Response bias in opinion polls and American social welfare. *The Social Science Journal*. 2006;43(1):99-110 DOI: 10.1016/j.soscij.2005.12.010
28 Kohler U. Possible Uses of Nonprobability Sampling for the Social Sciences. *Survey Methods: Insights from the Field*. Available at: https://surveyinsights.org/?p=10981 Accessed 29 July 2020.
29 Ebesutani C, Chorpita BF, Higa-Mcmillan CK, et al. A Psychometric Analysis of the Revised Child Anxiety and Depression Scales—Parent Version in a School Sample. *Journal of Abnormal Child Psychology*. 2011;39(2):173-85 DOI: 10.1007/s10802-010-9460-8

Confidential: For Review Only

30 Chorpita BF. Child FIRST - Focus on innovation and redesign in systems and treatment. Available at: https://www.childfirst.ucla.edu/resources/ Accessed 6 July 2020.

Confidential: For Review Only

SUPPLEMENTARY MATERIAL

Full Survey Material

D1. Please type your age on your last birthday: [number]

Screenout if D1 = <18

Q1. Which country do you live in? [single code]

a. England
b. Scotland
c. Wales
d. Northern Ireland
e. Outside of the UK

Screenout if Q1 = b-e

Q2. Which region do you live in? [single code]

a. East Midlands
b. East of England
c. London
d. North East
e. North West
f. South East
g. South West
h. West Midlands
i. Yorkshire and the Humber

Q3. Are you the parent or guardian of a child who usually attends school (that is, before the coronavirus outbreak occurred) and who lives with you? [single code]

a. Yes
b. No

Screenout if Q3 = b

Q4. Which of the following age groups do your children who live with you fall into? Please tick any that apply. [multi code]

a. 0-3
b. 4-11
c. 12-18
d. None of the above [exclusive]

Screenout if b OR c not selected

Q5. The Government has categorised some people as "critical workers" or "key workers." For example, this includes people working in health and social care, education, key public services, transport, food distribution and other jobs. As far as you know, are you, or a spouse/partner, a "critical" or "key" worker? Please tick any that apply. [multi code]

a. Yes, me
b. Yes, spouse/partner
c. No [exclusive]
d. Don't know [exclusive]

IF Q4 = b

Q6. You stated that you have at least one child aged 4-11 who lives with you. Are any of your children who live with you in the following school years? Please tick any that apply. [multi code]

a. Reception
b. Year 1

Confidential: For Review Only

c. Year 6
d. I have no children in the school years listed above [exclusive]

IF 2 or more options are selected at Q6

Q7. Thinking only about your children in Reception, Year 1 or Year 6, who live with you, which child had the most recent birthday? If the most recent birthday was shared by more than one child (e.g. twins, or children born on the same day in different years), please answer the following questions thinking about one of these children only. [single code]

a. My child in Reception
b. My child in Year 1
c. My child in Year 6

ONLY SHOW OPTIONS SELECTED AT Q6

IF Q6 = a-c

Q8. Can you please tell us how old that child is? [single code]

a. 4
b. 5
c. 6
d. 7
e. 8
f. 9
g. 10
h. 11

IF Q6 = a-c

Q9. Can you please tell us if that child is a: [single code]

a. Boy
b. Girl

IF Q6 = a-c

Q10. Can you please tell us the type of school that child usually attends? [single code]

a. Fee-paying
b. State-funded school
c. Don't know

IF Q6 = a-c

Q11A. Thinking about that child, have they attended school at all in the past 7 days? [single code]

a. Yes, on 5 days
b. Yes, on 1 to 4 days
c. No
d. Don't know

IF Q11A = a/b

Q12A. You said that your child has attended school on at least one day in the past week. For which reasons did you send your child to school? Please tick any that apply [multi code]:

RANDOMISE ORDER OF STATEMENTS, anchor other to bottom

a) It is compulsory for my child to attend
b) I need to work
c) I have non-work commitments I need to meet
d) My child's education will benefit from being at school
e) My child will benefit from seeing their friends
f) Having my child at home is a strain

Confidential: For Review Only

g) I don't believe my child will be at risk at school
h) My child wants to go to school
i) I think friends, family or other parents will judge me if I don't send my child to school
j) It reduces costs at home
k) Other reason [write in]

IF Q11A = b

Q13A. You said that your child has not attended school every day in the past week. For which reasons are they attending only part-time? Please tick any that apply [multi code]:

RANDOMISE ORDER OF STATEMENTS, anchor other to bottom

a) I am using it as childcare and I only need them in part time
b) It is less risky for them to be in part-time
c) The school only offers for them to be in part-time
d) I am only sending them in on days where the lessons are important
e) I am only sending them in on days when their friends are in
f) I am only sending them in on days when I can arrange transport
g) They only live with me part time
h) I started sending them in, but have now changed my mind
i) They have developed coronavirus symptoms (cough or fever, or change in sense of taste or smell)
j) My child developed symptoms of a different illness
k) Someone else in the family developed coronavirus symptoms (cough or fever, or change in sense of taste or smell)
l) Someone else at the school developed coronavirus symptoms (cough or fever, or change in sense of taste or smell)
m) Other reason [write in]

IF Q11A = c

Q14A. You said that your child has not attended school in the past week. For which reasons did you not send your child to school? Please tick any that apply [multi code]:

RANDOMISE ORDER OF STATEMENTS, anchor other to bottom

a) Someone in my household is clinically vulnerable to coronavirus
b) I think it is too risky for my child to attend school at the moment
c) The school is not open
d) The school has asked my child not to attend
e) My child doesn't want to go
f) I think friends, family or other parents will judge me if I send my child to school
g) I think they will get a better education at home than at school at the moment
h) Being at school will be stressful for my child
i) My child's friends will not be at school
j) I have another child who can't go to school
k) I can't arrange transport to get them to school
l) There's no point, schools will shut again soon anyway
m) They have developed coronavirus symptoms (cough or fever, or change in sense of taste or smell)
n) My child developed symptoms of a different illness
o) Someone else in the family developed coronavirus symptoms (cough or fever, or change in sense of taste or smell)
p) Someone else at the school developed coronavirus symptoms (cough or fever, or change in sense of taste or smell)
q) Other reason [write in]

IF Q11A = a/b

Q15A. In this question we are interested in things that happened on the most recent day that your child went to school. Please remember that this survey is anonymous – please be honest in your answers. Please tick any that apply. [multi code]

RANDOMISE ORDER OF STATEMENTS, anchor none of these to bottom

Confidential: For Review Only

a) Either on the way to or from school, or at the school gates, I had physical contact with someone that I don't live with
b) Either on the way to or from school, or at the school gates, I was within 1 metre of someone that I don't live with for 1 minute or longer
c) Either on the way to or from school, or at the school gates, I was between 1 and 2 metres of someone that I don't live with for 15 minutes or longer
d) My child used public transport to get to or from school.
e) My child shared a lift in a car with another family to get to or from school.
f) I gave a lift in a car to a child from another family to get them to or from school.
g) My child washed their hands as soon as they got home from school.
h) My child had a shower or bath as soon as they got home from school.
i) I washed my child's clothes after they got home from school.
j) None of these [exclusive]

IF Q11A = a/b

Q16A. Thinking about the facilities or procedures at your child's school. Which of the following, if any, are actually happening as far as you are aware?

Please tick all that apply [multi code]

RANDOMISE ORDER OF STATEMENTS, anchor none of these to bottom

a) There are hand washing facilities or hand gel dispensers at the entrance to the school that are working
b) There are hand washing facilities or hand gel dispensers at the entrance to the classrooms that are working
c) Children's hand washing or hand gel use is being monitored at school
d) My child's class sizes is now 15 or fewer
e) Children need to take in their own food and snacks
f) Children are having their temperature checked on the way in
g) The school has used markings or barriers to help children keep their distance from each other
h) None of these are happening at my child's school [exclusive]

Q17. For the next series of questions, we would like you to think about one of your children who usually goes to school (that is, before the coronavirus outbreak occurred). If you have more than one child who usually went to school, then please answer about your child with the most recent birthday. If the most recent birthday was shared by more than one child (e.g. twins, or children born on the same day in different years), please answer the following questions thinking about one of these children only.

Firstly, can you tell us the first name of your child. This is just so we can refer to them throughout the survey. You can give a fake name if you would prefer. [open]

____

Q18. How old is [CHILD]? [single code]

a. 4
b. 5
c. 6
d. 7
e. 8
f. 9
g. 10
h. 11
i. 12
j. 13
k. 14
l. 15
m. 16
n. 17
o. 18

Confidential: For Review Only

Q19. What year is [CHILD] in at school [single code]

a. Reception
b. Year 1
c. Year 2
d. Year 3
e. Year 4
f. Year 5
g. Year 6
h. Year 7
i. Year 8
j. Year 9
k. Year 10
l. Year 11
m. Year 12
n. Year 13

Q20. Is [CHILD] a: [single code]

a. Boy
b. Girl

Q21. What type of school does [CHILD] normally attend? [single code]

a. Fee-paying
b. State-funded school
c. Don't know

If Q5 = a/b AND [Q4 ≠ b or Q6 = d)

Q11B. Has [CHILD] attended school at all in the past 7 days? [single code]

a. Yes, on 5 days
b. Yes, on 1 to 4 days
c. No
d. Don't know

IF Q11B = a/b

Q12B. You said that [CHILD] has attended school on at least one day in the past week. For which reasons did you send [CHILD] to school? Please tick any that apply [multi code]:

RANDOMISE ORDER OF STATEMENTS, anchor other to bottom

a) It is compulsory for my child to attend
b) I need to work
c) I have non-work commitments I need to meet
d) My child's education will benefit from being at school
e) My child will benefit from seeing their friends
f) Having my child at home is a strain
g) I don't believe my child will be at risk at school
h) My child wants to go to school
i) I think friends, family or other parents will judge me if I don't send my child to school
j) It reduces costs at home
k) Other reason [write in]

IF Q11B = b

Q13B. You said that [CHILD] has not attended school every day in the past week. For which reasons is [CHILD] attending only part-time? Please tick any that apply [multi code]:

RANDOMISE ORDER OF STATEMENTS, anchor other to bottom

a) I am using it as childcare and I only need them in part time
b) It is less risky for them to be in part-time

Confidential: For Review Only

c) The school only offers for them to be in part-time
d) I am only sending them in on days where the lessons are important
e) I am only sending them in on days when their friends are in
f) I am only sending them in on days when I can arrange transport
g) They only live with me part time
h) I started sending them in, but have now changed my mind
i) They have developed coronavirus symptoms (cough or fever, or change in sense of taste or smell)
j) My child developed symptoms of a different illness
k) Someone else in the family developed coronavirus symptoms (cough or fever, or change in sense of taste or smell)
l) Someone else at the school developed coronavirus symptoms (cough or fever, or change in sense of taste or smell)
m) Other reason [write in]

IF Q11B = c

Q14B. You said that [CHILD] has not attended school in the past week. For which reasons did you not send [CHILD] to school? Please tick any that apply [multi code]:

RANDOMISE ORDER OF STATEMENTS, anchor other to bottom

a) Someone in my household is clinically vulnerable to coronavirus
b) I think it is too risky for my child to attend school at the moment
c) The school is not open
d) The school has asked my child not to attend
e) My child doesn't want to go
f) I think friends, family or other parents will judge me if I send my child to school
g) I think they will get a better education at home than at school at the moment
h) Being at school will be stressful for my child
i) My child's friends will not be at school
j) I have another child who can't go to school
k) I can't arrange transport to get them to school
l) There's no point, schools will shut again soon anyway
m) They have developed coronavirus symptoms (cough or fever, or change in sense of taste or smell)
n) My child developed symptoms of a different illness
o) Someone else in the family developed coronavirus symptoms (cough or fever, or change in sense of taste or smell)
p) Someone else at the school developed coronavirus symptoms (cough or fever, or change in sense of taste or smell)
q) Other reason [write in]

IF Q11B = a/b

Q15B. In this question we are interested in things that happened on the most recent day that [CHILD] went to school. Please remember that this survey is anonymous – please be honest in your answers. Please tick any that apply. [multi code]

RANDOMISE ORDER OF STATEMENTS, anchor none of these to bottom

a) Either on the way to or from school, or at the school gates, I had physical contact with someone that I don't live with
b) Either on the way to or from school, or at the school gates, I was within 1 metre of someone that I don't live with for 1 minute or longer
c) Either on the way to or from school, or at the school gates, I was between 1 and 2 metres of someone that I don't live with for 15 minutes or longer
d) My child used public transport to get to or from school.
e) My child shared a lift in a car with another family to get to or from school.
f) I gave a lift in a car to a child from another family to get them to or from school.
g) My child washed their hands as soon as they got home from school.
h) My child had a shower or bath as soon as they got home from school.
i) I washed my child's clothes after they got home from school.
j) None of these [exclusive]

Confidential: For Review Only

IF Q11B = a/b

Q16B. Thinking about the facilities or procedures at [CHILD]'s school. Which of the following, if any, are actually happening as far as you are aware? Please tick any that apply. [multi code]

RANDOMISE ORDER OF STATEMENTS, anchor none of these to bottom

a) There are hand washing facilities or hand gel dispensers at the entrance to the school that are working
b) There are hand washing facilities or hand gel dispensers at the entrance to the classrooms that are working
c) Children's hand washing or hand gel use is being monitored at school
d) My child's class size is now 15 or fewer
e) Children need to take in their own food and snacks
f) Children are having their temperature checked on the way in
g) The school has used markings or barriers to help children keep their distance from each other
h) None of these are happening at [CHILD]'s school [exclusive]

Q22. Does [CHILD] have special educational needs? [singe code]

- Yes
- No
- Don't know / prefer not to say

Q23. In the past 7 days, how many times, if at all, has [CHILD] left your home for each of the following reasons? [multi code]

a) To go to the shops for groceries, toiletries or medicines
b) To go to the shops for other items
c) For exercise
d) For a medical need (e.g. an outpatient appointment)
e) To go to school
f) To provide help to someone else
g) To meet friends
h) To meet family members who don't live with you
i) For another reason

Q24. In the past 7 days, have you done the following, because of the risk from coronavirus:

SCALE

- Yes
- No

STATEMENTS – RANDOMISE

a. Washed your hands thoroughly and regularly
b. Stayed 2m (3 steps) away from people you do not live with when outside your home
c. Washed your clothes when you have returned home
d. Washed [CHILD]'s clothes when she/ he has returned home
e. Used tissues or clothing to touch door handles or other objects when outside
f. Cleaned or disinfected items you have brought into the home, such as groceries or parcels
g. Left items that you have brought into the home for 24 hours or more before using them, to kill any virus on them
h. Covered your face or used a face mask when out and about
i. Covered [CHILD's] face or given her/him a face mask when out and about
j. Worn protective gloves when out and about
k. Made [CHILD] wear protective gloves when out and about

https://mc.manuscriptcentral.com/bmjpo

Confidential: For Review Only

Q25. We are interested in how many people [CHILD] has had close contact with in the past 24 hours. By close contact we mean closer than 2 meters, for fifteen minutes or more. Please remember, this survey is anonymous so please be honest.

In the past 24 hours, how many people in the following categories has [CHILD] had close contact with: [multi code]

a. Someone [CHILD] lives with
b. Friends or other children who [CHILD] does not live with
c. A family member aged under 70 who [CHILD] does not live with
d. A family member aged over 70 who [CHILD] does not live with
e. A babysitter, nanny or childminder
f. Other children, not already reported above
g. Other adults, not already reported above

Q26. Did [CHILD] attend school in the past 24 hours? [single code]

a. Yes
b. No

RANDOMISE ORDER OF Q27a AND Q27b SHOWN TO EACH RESPONDENT

Q27a. How much, if at all, do you agree or disagree with the following statements:

STATEMENTS (randomise order):

a) If [CHILD] goes out, she/he is likely to catch coronavirus
b) If [CHILD] goes out, she/he is likely to bring coronavirus back into our home
c) [CHILD] is keeping up with her/his schoolwork
d) I feel confident helping [CHILD] with her/his schoolwork
e) I feel supported by [CHILD's] school
f) I have access to all the resources that [CHILD] needs to do her/his schoolwork
g) During lockdown, [CHILD] has learned about important things she/he wouldn't normally learn at school.
h) In the past 7 days, [CHILD] has been bored

SCALE

- Strongly agree
- Tend to agree
- Neither agree nor disagree
- Tend to disagree
- Strongly disagree
- Not applicable

Q27b. How much, if at all, do you agree or disagree with the following statements:

STATEMENTS (randomise order):

a) In the past 7 days, my household has had a regular structure to the day
b) In the past 7 days, [CHILD] has kept in touch with her/his friends
c) [CHILD] is worried about coronavirus
d) In the past 7 days, [CHILD] has felt upset about not seeing other family members who do not live with us
e) In the past 7 days, I have found it hard to keep up with work or other important commitments
f) In the past 7 days, people in my household have been getting along well
g) I am worried about the financial impact of lockdown measures
h) Before the school closures, [CHILD] had extra support at school

SCALE

- Strongly agree
- Tend to agree

https://mc.manuscriptcentral.com/bmjpo

Confidential: For Review Only

- Neither agree nor disagree
- Tend to disagree
- Strongly disagree
- Not applicable

Q28. To what extent, if at all, do you agree or disagree with the following statements?

RANDOMISE ORDER OF STATEMENTS

a) If it were possible, I would feel comfortable sending [CHILD] to school next week.
b) If it were possible, I would want to send [CHILD] to school next week.
c) There is still too much coronavirus around for schools to be able to reopen safely
d) When [CHILD's] school fully reopens, some parents will send their children to school, even if they have symptoms of coronavirus
e) It is impossible for children to maintain 'social distancing' at school
f) Children will not wash their hands properly at school
g) Staff will not wash their hands properly at school
h) Schools will not be able to provide good quality education at the moment
i) School will not be an enjoyable place for [CHILD] to be at the moment

SCALE

- Strongly agree
- Tend to agree
- Neither agree nor disagree
- Tend to disagree
- Strongly disagree
- Not applicable

Q29A. Has [CHILD] had any of the following symptoms in the past 7 days? Please tick any that apply, no matter where you think the symptoms came from, and even if the symptoms were mild. [multi code]

RANDOMISE ORDER OF STATEMENTS, anchor "none of these" and "don't know" to bottom

- New, continuous cough
- High temperature / fever
- Runny nose
- Diarrhoea
- Nausea / feeling sick
- Vomiting
- Sneezing
- Loss of appetite
- Loss of sense of smell (fully or partial)
- Loss of taste
- None of these
- Don't know

Q29B. Have you, or anyone else in your household (excluding [CHILD]) had any of the following symptoms in the past 14 days? Please tick any that apply, no matter where you think the symptoms came from, and even if the symptoms were mild. [multi code]

RANDOMISE ORDER OF STATEMENTS, anchor "none of these" and "don't know" to bottom

- New, continuous cough
- High temperature / fever
- Runny nose
- Diarrhoea
- Nausea / feeling sick
- Vomiting
- Sneezing
- Loss of appetite
- Loss of sense of smell (fully or partial)

Confidential: For Review Only

- Loss of taste
- None of these
- Don't know

Q30. Do you know if [CHILD] has had, or currently has, coronavirus? (Please select the option that BEST applies) [single code]

a) They have definitely had it or definitely have it now
b) They have probably had it or probably have it now
c) They have probably not had it and probably don't have it now
d) They have definitely not had it and definitely don't have it now

RANDOMISE ORDER OF Q31a AND Q31b SHOWN TO EACH RESPONDENT

Q31a. Please tell us how often each of these things happen to [CHILD]. There are no right or wrong answers.
SCALE

- *Never*
- *Sometimes*
- *Often*
- *Always*

STATEMENTS
- My child worries about things
- My child worries that something awful will happen to someone in the family
- My child worries that bad things will happen to him/her
- My child feels sad or empty
- Nothing is much fun for my child anymore
- My child has trouble sleeping
- My child has problems with his/her appetite
- My child has no energy for things

Q31b. Please tell us how often each of these things happen to [CHILD]. There are no right or wrong answers.
SCALE

- *Never*
- *Sometimes*
- *Often*
- *Always*

STATEMENTS

- My child worries that something bad will happen to him/her
- My child worries about what is going to happen
- My child thinks about death
- My child is tired a lot
- My child cannot think clearly
- My child feels worthless
- My child feels like he/she doesn't want to move
- My child feels restless

Q32. Thinking about next flu season (September 2020 – March 2021). During this time, you may be invited to vaccinate [CHILD] against flu. All primary school, but not secondary school children, are eligible for the child flu vaccine. For the following questions, please think about the next flu season.

For each of the following statements, please tell us to what extent, if at all, you agree or disagree:

I want [CHILD] to be vaccinated for flu next year (2020/21)

- Strongly agree
- Agree
- Neither agree nor disagree

Confidential: For Review Only

- Disagree
- Strongly disagree
- Not applicable, [CHILD] will be in secondary school in next school year (2020/21)

IF Q32 = a-e

Q33 I intend [CHILD] to be vaccinated for flu next year (2020/21)

- Strongly agree
- Agree
- Neither agree nor disagree
- Disagree
- Strongly disagree
- Not applicable, [CHILD] will be in secondary school in next school year (2020/21)

Thinking now about yourself…

Q34. Over the last 2 weeks, how often have you been bothered by the following problems?

SCALE

- Not at all
- Several days
- More than half the days
- Nearly every day

STATEMENTS

- Feeling nervous, anxious or on edge
- Not being able to stop or control worrying
- Little interest or pleasure in doing things
- Feeling down, depressed, or hopeless

And finally, the following questions ask for some more information about you and your household.

D2. Which gender do you identify yourself with? [single code]

- Male
- Female
- Prefer to self-describe
- Prefer not to say

D3. What is your employment status? [single code]

a. Full time paid job (31+ hours)
b. Part time paid job (<31 hours)
c. Doing paid work on a self-employed basis or within your own business
d. Employed, but currently furloughed
e. Student / On a government training programme (Nation Traineeship/Modern Apprenticeship)
f. Out of work (6 months or less)
g. Out of work (more than 6 months)
h. Looking after home / Homemaker
i. Retired
j. Disabled OR Long-term sick
k. Unpaid work for a business, community or voluntary organisation
l. Prefer not to say

If answer a/b/c to D3

D4. Are you currently working from home? [single code]

- Yes
- No

Confidential: For Review Only

D5. What is the highest level of educational qualification you have received? [single code]

a. PhD/Doctor
b. Master's
c. Bachelor's Degree or equivalent (Such as a NVQ level 5)
d. Higher education (Such as a HND or a NVQ level 4)
e. A level or equivalent (Such as Scottish Highers or NVQ level 3)
f. GCSE and below (Such as O level or an RSA Diploma)
g. Other qualifications (Such as NVQ level 1)
h. No qualifications
i. Prefer not to say

D6. Which of the following income brackets best represents your household income, before tax deductions for income tax, National Insurance etc? [single code]

a. Less than £5,000
b. £5,000-£9,999
c. £10,000-£14,999
d. £15,000-£19,999
e. £20,000-£24,999
f. £25,000-£29,999
g. £30,000-£34,999
h. £35,000-£39,999
i. £40,000-£44,999
j. £45,000-£49,999
k. £50,000-£59,999
l. £60,000-£69,999
m. £70,000-£84,999
n. £85,000-£99,999
o. More than £100,000
p. Prefer not to say

D7. What is your current marital or civil partnership status? [single code]

a. Single (i.e. never married and never registered as a same sex civil-partnership)
b. Co-habiting with partner (but never married or been in a civil partnership)
c. Civil partnership
d. Married
e. Separated, but still legally married / in a civil partnership
f. Divorced / Civil partnership legally dissolved
g. Widowed / Surviving partner from a same-sex civil partnership

D8. Which of the following categories would best describe your ethnicity? [single code]

a. English/Welsh/Scottish/Northern Irish/British
b. Irish
c. Gypsy or Irish Traveller
d. Other
e. White and Black Caribbean
f. White and Black African
g. White and Asian
h. Other
i. Indian
j. Pakistani
k. Bangladeshi
l. Chinese
m. Other
n. Caribbean
o. African
p. Other
q. Arab
r. Any other (please specify)
s. Prefer not to say

Confidential: For Review Only

D10. How many children in each age group live in your household? [number list]

- 0-3
- 4-10
- 11-15
- 16-18

Allow numerical input for each age group

D11. Do you live with anyone over the age of 70 years? [single code]

a. Yes
b. No

D12. Including yourself, how many people live in your household? [single code]

a. 2
b. 3
c. 4
d. 5
e. 6
f. 7
g. 8
h. 9
i. 10 or more

D9. Do any of the following have a medical condition than might make them particularly vulnerable to coronavirus?

SCALE

- Yes
- No
- Don't know

STATEMENTS

a. Yourself
b. [CHILD]
c. Anyone else you live with [show if D12 = b-i]

D13. [Record number] How many bedrooms does your home have?

D14. [Multi code] Which of the following do you have access to at your home:

a) A garden
b) A patio / terrace
c) A balcony
d) Another form of outdoor space
e) No outdoor space

Thank you for completing this survey. If you have any concerns about your child's mental health, please click here.

Confidential: For Review Only

Recoding of variables

We recoded all statement answers into binary variables by grouping “strongly agree” and “tend to agree”, versus “neither agree nor disagree”, “tend to disagree” and “strongly disagree”.

We created two binary variables to indicate whether the child, and someone in the household (other than the child) had a health condition that might make them particularly vulnerable to COVID-19.

We created two binary variables to indicate presence of COVID-19 symptoms in the child, and someone else in the household. We defined presence of COVID-19 symptoms as experiencing a “new, continuous cough”, “high temperature / fever”, “loss of sense of smell (fully or partial)”, or “loss of taste”. We coded a binary variable for the parent’s perception of whether the child had had COVID-19 by grouping together “they have definitely had it or definitely have it now” and “they have probably had it or probably have it now”.

We created a binary variable to indicate low well-being in the child. We assigned a value against each answer from 0 (“never”) to 3 (“always”) on the GAD and MDD RCADS sub-scales and created a total score for each sub-scale. We then turned each total score into a t-score, a method used to normalise RCADS scores within the population, by child’s age and gender.[20,30] We used the same process for reception to year three as for year four. We used a t-score cut off of 65 or above on either GAD or MDD sub-scales to indicate low well-being.

For all variables, we coded the responses “don’t know”, “not applicable”, “prefer not to say” and “prefer to self-describe” as missing data.

Confidential: For Review Only

BMJ Paediatrics Open

Why did some parents not send their children back to school following school closures during the COVID-19 pandemic: a cross-sectional survey

Journal:	*BMJ Paediatrics Open*
Manuscript ID	bmjpo-2020-001014.R1
Article Type:	Original research
Date Submitted by the Author:	29-Mar-2021
Complete List of Authors:	Woodland, Lisa; Weston Education Centre<br>Smith, Louise; King's College London<br>Webster, Rebecca K; The University of Sheffield<br>Amlôt, Richard; Public Health England<br>Rubin, Antonia; Weald of Kent Grammar School<br>Wessely, Simon; Institute of Psychiatry Psychology and Neuroscience<br>Rubin, James
Keywords:	COVID-19, Psychology

SCHOLARONE™
Manuscripts

BMJ

I, the Submitting Author has the right to grant and does grant on behalf of all authors of the Work (as defined in the below author licence), an exclusive licence and/or a non-exclusive licence for contributions from authors who are: i) UK Crown employees; ii) where BMJ has agreed a CC-BY licence shall apply, and/or iii) in accordance with the terms applicable for US Federal Government officers or employees acting as part of their official duties; on a worldwide, perpetual, irrevocable, royalty-free basis to BMJ Publishing Group Ltd ("BMJ") its licensees and where the relevant Journal is co-owned by BMJ to the co-owners of the Journal, to publish the Work in this journal and any other BMJ products and to exploit all rights, as set out in our licence.

The Submitting Author accepts and understands that any supply made under these terms is made by BMJ to the Submitting Author unless you are acting as an employee on behalf of your employer or a postgraduate student of an affiliated institution which is paying any applicable article publishing charge ("APC") for Open Access articles. Where the Submitting Author wishes to make the Work available on an Open Access basis (and intends to pay the relevant APC), the terms of reuse of such Open Access shall be governed by a Creative Commons licence – details of these licences and which Creative Commons licence will apply to this Work are set out in our licence referred to above.

Other than as permitted in any relevant BMJ Author's Self Archiving Policies, I confirm this Work has not been accepted for publication elsewhere, is not being considered for publication elsewhere and does not duplicate material already published. I confirm all authors consent to publication of this Work and authorise the granting of this licence.

Confidential: For Review Only

Why did some parents not send their children back to school following school closures during the COVID-19 pandemic: a cross-sectional survey

Lisa Woodland MSc, King's College London, Weston Education Centre, Cutcombe Road, London SE5 9RJ, UK

Louise E Smith PhD, King's College London, Weston Education Centre, Cutcombe Road, London SE5 9RJ, UK

Rebecca K Webster PhD, University of Sheffield, Cathedral Court, 1 Vicar Lane, Sheffield, S1 2LT, UK

Richard Amlôt PhD, Public Health England, Emergency Response Department, Porton Down, Salisbury, SP4 0JG, UK

Antonia Rubin MA (Cantab), Trustee at Weald of Kent Grammar School, Tudeley Lane, Tonbridge, Kent, TN9 2JP, UK.

Simon Wessely PhD, King's College London, Weston Education Centre, Cutcombe Road, London SE5 9RJ, UK

G James Rubin PhD, King's College London, Weston Education Centre, Cutcombe Road, London SE5 9RJ, UK

Correspondence to: Lisa Woodland, King's College London, Weston Education Centre, Cutcombe Road, London SE5 9RJ, UK, lisa.woodland@kcl.ac.uk

Word Count: 2,637

Confidential: For Review Only

ABSTRACT

Background

On 23rd March 2020, schools closed to most children in England in response to COVID-19 until September 2020. Schools were kept open to children of key workers and vulnerable children, on a voluntary basis. Starting 1st June 2020 children in reception (four to five years old), year one (five to six years old) and year six (10 to 11 years old) also became eligible to attend school.

Methods

1,373 parents or guardians of children eligible to attend school completed a cross-sectional survey between 8th and 11th June 2020. We investigated factors associated with whether children attended school or not.

Results

46% (n=370 / 803) of children in year-groups eligible to attend school and 13% (n=72 / 570) of children of key workers had attended school in the past seven days. The most common reasons for sending children to school were that the child's education would benefit, the child wanted to go to school, and the parent needed to work. A child was significantly more likely to attend if the parent believed the child had already had COVID-19, they had special educational needs, or a person in the household had COVID-19 symptoms.

Conclusions

Following any future school closure, helping parents to feel comfortable returning their child to school will require policy makers and school leaders to communicate about the adequacy of their policies to: a) ensure that the risk to children in school is minimised; b) ensure that the educational potential within schools is maximised; and c) ensure that the benefits of school for the psychological well-being of children are prioritised.

Key Words

Child; COVID-19; parents; school closures; England; cross-sectional studies

Confidential: For Review Only

ARTICLE ADDS

What is known about the subject

The COVID-19 pandemic resulted in the mass closure of schools for an extended period of time. A previous systematic review assessed parental attitudes towards smaller scale closures, finding that many parents (71%-97% across six studies) approved of closures, particularly where they were seen as an effective protective measure against a serious illness. The effect on a child's education and ability of the parent to work were reported as concerns.

What this study adds

During the partial reopening of schools in England in June 2020, most parents did not send their children to school. Parents who were not educated to degree level, not working, who lived in the North of England or who were from Black, Asian and ethnic minority backgrounds were least likely to send their children back. Education, risk of disease and children's well-being were the main drivers in determining parental decision to send them to school or not.

Confidential: For Review Only

INTRODUCTION

On 23rd March 2020, a nationwide closure of schools occurred across England in response to the COVID-19 pandemic. Only vulnerable children (those with a health care plan or social worker) and children of key workers (critical to the COVID-19 response) were able to attend.[1,2] From 1st June 2020 children in reception (four to five years), year one (five to six years) and year six (10 to 11 years) also became eligible to attend.[3] Until September 2020 school attendance was voluntary to those children eligible to attend.[4]

The benefits of closing schools to reduce the transmission of COVID-19 and the negative consequences of doing so were difficult to balance.[5,6] Adding to the debate was emerging evidence of a low transmission rate of COVID-19 among children [7-10] and a recognition that outbreaks may occur nonetheless.[11,12]

Irrespective of this debate, it was clear that many parents felt far from comfortable with their children attending school in the first months of the pandemic, even where it was encouraged.[13] A worldwide systematic review of school closures suggests several factors that may be relevant to whether a child attended school during an infectious disease outbreak.[14] Nineteen papers were included in the review, samples representing between 67 and 4,171 school-aged children (five to 19 years). Perceived risk of infection,[15,16] concern about the impact of a closure on education,[15,17] and parental concerns about their child's mental health were key issues.[18] Understanding the key issues that determine whether a parent is willing to send their children back to school when it is partially open, and ensuring that school policies and communications address these concerns, should help inform re-opening schools, in this or any future pandemic.

In this study, we investigated factors associated with a parent's willingness to send their child to school when they partially re-opened, following closures due to the COVID-19 pandemic. We investigated these factors for children in reception, year one, or year six and for families where at least one parent was a key worker.

METHODS

Design

We commissioned a market research company to conduct a cross-sectional survey one week after schools in England re-opened for children in reception, year one, and year six (8th to 11th June 2020). We have previously reported data from this survey relating to parental perceptions of the presence of hygiene procedures within schools.[19]

Participants

Participants (n=2,447) were recruited from BMG Research's panel. In order to achieve a sample broadly representative of the population, BMG Research monitored region, child age, child gender, parent/guardian age, and parent/guardian gender. Participants were eligible for the study if they were aged eighteen years or

Confidential: For Review Only

over, lived in England, and were a parent or guardian to a school-aged child (4-18 years) who usually lived with them.[20] 183 participants were screened out for non-eligibility, 226 participants dropped out after starting the survey and 28 completed but were removed for quality control such as completing the survey quickly or for ‘straight-lining’ (selecting the same option for every question) suggesting inattention to the questions. 2,010 participants remained. The sample fell within five percentage points of the national population by the child’s gender, key stage, and type of school attended against the known distribution for school children in England.[21] The sample used in this paper were 803 parents of children in eligible school years and 570 parents from families in which at least one parent was a key worker (9 participants were removed from this group due to logical inconsistencies which suggested they had accidently completed the wrong section).

Participants were paid equivalent to £0.60.

Study Materials

The full survey is available in the supplementary materials.

Selection of a child to answer questions about

All participants were asked to select their child with the most recent birthday to answer questions about. In cases where children shared a birthday, we asked the parent to select one child.

Our survey had two sections. Section one was only completed by parents who had a child in reception, year one, or year six or by parents who did not have a child in these year groups, but they or their spouse were a key worker. It contained questions about whether the child had attended school in the past week. Section two was completed by these parents and also by parents who did not have a child eligible to attend school. It contained general questions on views about risk of COVID-19, family living and school safety measures. In this paper we only report data from section one, relating to actual attendance in the past week.

Personal Characteristics

We asked participants to report their gender, age, region, household income, employment status, marital status, ethnicity, and level of education. We also asked whether anyone within the household was aged over 70 years or had a health condition that made them vulnerable to COVID-19.

We asked participants to report the child’s gender, age, school year, school type (fee-paying or state-funded) and whether the child had special educational needs (SEN).

School Attendance

Participants were asked how many times the child had attended school in the past seven days. Depending on the response, parents were presented with randomised statements: 10 for why they were sending the child to school; 12 for why they were only sending the child to school part-time; or 16 why their child was not attending

Confidential: For Review Only

school. We asked participants to “tick any [statement] that applies”. Participants also had the option to write-in text for “other reason”.

COVID-19 Symptoms

We asked participants to report if the selected child had experienced any symptoms “in the past seven days” from a list of ten symptoms. We also asked if they or a household member (other than the child) had experienced symptoms “in the past 14 days” from the same symptom list. We asked participants whether they thought their child had had COVID-19.

Well-Being

We asked participants to report the child’s well-being using two subscales from the Revised Child Anxiety Disorder Scale (RCADS):[22] the generalised anxiety disorder (GAD) sub-scale and the major depressive disorder (MDD) sub-scale.

ETHICS

The research was approved by the Psychiatry, Nursing and Midwifery Research Ethics Subcommittee at King’s College London (LRS—19/20-18787).

PATIENT AND PUBLIC INVOLVEMENT.

A school trustee contributed to the development of the survey materials and co-authored this paper.

ANALYSIS

Recoding of variables

School attendance was defined as a child who attended school for at least one day, in the past seven days.

We created two binary variables to indicate whether the child, and someone in the household (other than the child) had a health condition that might make them particularly vulnerable to COVID-19.

We created two binary variables to indicate recent presence of COVID-19 symptoms in the child, and someone else in the household. We defined presence of COVID-19 symptoms as experiencing a “new, continuous cough”, “high temperature / fever”, “loss of sense of smell (fully or partial)”, or “loss of taste”. We coded a binary variable for the parent’s perception of whether the child had had COVID-19 by grouping together “they have definitely had it or definitely have it now” and “they have probably had it or probably have it now”.

We created a binary variable to indicate low well-being in the child. We assigned a value against each answer from 0 (“never”) to 3 (“always”) on the GAD and MDD RCADS sub-scales and created a total score for each sub-scale. We then turned each total score into a t-score, a method used to normalise RCADS scores within the

Confidential: For Review Only

population, by child's age and gender.[22,23] We used the same process for reception to year three as for year four. We used a t-score cut off of 65 or above on either GAD or MDD sub-scales to indicate low well-being.

For all variables, we coded the responses "don't know", "not applicable", "prefer not to say" and "prefer to self-describe" as missing data.

Analysis

We ran a series of binary logistic regressions using SPSS v26.0,[24] investigating univariable associations between each of our predictor variables and sending the child to school. We ran a second set of binary logistic regressions controlling for personal characteristics shown in the results.

We analysed frequencies for the statements responding to sending the child to school for a full week (five days), part-time (less than five days), and for not sending the child to school.

For ease of interpretation, we used unweighted data in our analysis.

We applied a Bonferroni correction to our results ($p \leq .001$) due to running many analyses (children in school years n=29 and children of key workers n=31). Results meeting this criterion are marked by a double asterisk (**) in the tables.

Sample size calculation

Post-hoc calculations were run on the two sub-samples which had a margin of error of plus or minus 5% at the 95% confidence level for each prevalence estimate.

RESULTS

School attendance

Nearly half (46% CI, 43% to 50%, n=370/803) of children in eligible school years had attended school and about half had not (54% 95% CI, 50% to 57%, n=432/803). One participant was unsure if the child had attended school. The most common reasons for not sending a child to school were: thinking it was too risky for the child to attend school (n=223, 52%), the school not being open (n=140, 32%) and having another child who could not go to school (n=67, 16%).The most common reasons for sending a child to school were: the child's education would benefit (n=208, 56%), the child wants to go to school (n=200, 54%), and the child will benefit from seeing their friends (n=187, 51%). Of participants whose child did not attend school for a full week (n=204, 25%) the most common reasons for partial attendance were: the school only offers them to be in part-time (n=80, 39%), it is less risky for them to be in part-time (n=40, 20%), and I am only sending them in on days where the lessons are important (n=28, 14%).

Only 13% (95% CI, 10% to 15%, n=72/570) of children of key workers had attended school, most children had not (87% 95% CI, 85% to 90%, n = 497/570). One participant was unsure if the child had attended school. The most common reasons

Confidential: For Review Only

for children not attending school were: the school was not open (n=259, 52%), the school had asked the child not to attend (n=117, 24%), and thinking that it was too risky for the child to attend school at the moment (n=109, 22%). The most common reasons for attending school were: the parent needing to work (n=40, 56%), the child wanting to go to school (n=35, 49%), and thinking that the child's education would benefit from being at school (n=30, 42%). Of participants with children who attended school part-time the most common reasons were: the school only offered them to be in part-time (n=16, 41%), parent only sent them in on days where the lessons are important (n=8, 21%), and believing it was less risky for them to be in part-time (n=7, 18%).

Associations

Participant characteristics for children in eligible school years and for children of key workers are shown in table 1. Parents of children in eligible school years were less likely to send the child to school if they were educated to A-level or below, not working, of Black, Asian and minority ethnicity (BAME), or living in the North East, North West, and Yorkshire and the Humber compared to London. Whereas children of key workers were more likely to attend school for participants aged 45 and under and who were working. Child attendance was more likely for children in eligible school years when in a fee-paying school and if they had a health condition that made them vulnerable to COVID-19. For both groups, attendance was more likely for children who had special educational needs, reported low well-being, thought their child had had COVID-19 and when a person over 70 was living in the household. There was also a significant association in both groups between a child being more likely to attend school and the child having experienced COVID-19 symptoms in the past 7 days, or another person in the household having experienced COVID-19 symptoms in the past 14 days.

Confidential: For Review Only

Table 1. Participant characteristics for children in eligible school years (n=803) and children of key works (n=570), by school attendance.

		Children in Eligible school years				Children of key workers			
	Level	Did not attend school, n (%)	Attended School, n (%)	Odds ratio (95% CI)	Adjusted odds ratio (95% CI) †	Did not attend school, n (%)	Attended School, n (%)	Odds ratio (95% CI)	Adjusted odds ratio (95% CI) †
Gender	Male	201 (52)	187 (48)	1·22 (0·92 to 1·61)	1·04 (0·75 to 1·43)	227 (85)	39 (15)	1·53 (0·92 to 2·55)	1·61 (0·91 to 2·85)
	Female	230 (57)	177 (43)	Reference	Reference	268 (90)	30 (10)	Reference	Reference
Age	18-35 years	129 (49)	136 (51)	1·37 (0·92 to 2·03)	1·23 (0·79 to 1·92)	54 (77)	16 (23)	**3·16* (1·56 to 6·37)**	**2·95* (1·23 to 7·08)**
	36-45 years	212 (56)	164 (44)	1·01 (0·70 to 1·46)	0·98 (0·65 to 1·48)	198 (86)	33 (14)	**1·76* (1·01 to 3·12)**	**2·14* (1·12 to 4·09)**
	46 years ≥	91 (56·5)	70 (43·5)	Reference	Reference	245 (91)	23 (9)	Reference	Reference
Region	East Midlands	33 (60)	22 (40)	**0·50* (0·27 to 0·93)**	0·59 (0·31 to 1·14)	45 (85)	8 (15)	0·85 (0·32 to 2·22)	1·23 (0·40 to 3·75)
	East of England	38 (48)	41 (52)	0·81 (0·48 to 1·38)	0·92 (0·52 to 1·64)	54 (82)	12 (18)	1·06 (0·45 to 2·52)	1·38 (0·50 to 3·81)
	North East	33 (67)	16 (33)	**0·36* (0·19 to 0·71)**	**0·38* (0·81 to 0·78)**	32 (89)	4 (11)	0·60 (0·18 to 1·98)	0·93 (0·25 to 3·46)
	North West	68 (65)	36 (35)	**0·40** (0·24 to 0·66)**	**0·39* (0·23 to 0·68)**	80 (93)	6 (7)	**0·36* (0·13 to 0·99)**	0·61 (0·19 to 1·89)
	South East	70 (51)	66 (49)	0·71 (0·45 to 1·11)	0·92 (0·56 to 1·52)	82 (89)	10 (11)	0·58 (0·24 to 1·94)	0·81 (0·29 to 2·29)
	South West	24 (46)	28 (54)	0·88 (0·47 to 1·63)	1·16 (0·58 to 2·31)	42 (87·5)	6 (12·5)	0·68 (0·42 to 1·94)	1·18 (0·37 to 3·82)
	West Midlands	39 (56)	30 (44)	0·58 (0·33 to 1·01)	0·63 (0·34 to 1·18)	48 (89)	6 (11)	0·60 (0·21 to 1·68)	0·84 (0·27 to 2·60)
	Yorkshire & the Humber	52 (62)	31 (37)	**0·45* (0·26 to 0·76)**	**0·52* (0·29 to 0·93)**	52 (88)	7 (12)	0·64 (0·24 to 1·73)	0·80 (0·27 to 2·41)
	London	75 (43)	100 (57)	Reference	Reference	62 (83)	13 (17)	Reference	Reference
Household income	≤ £34,999	164 (54)	142 (46	1·00 (0·75 to 1·33)	1·34 (0·95 to 1·90)	185 (89)	23 (11)	0·75 (0·44 to 1·28)	0·96 (0·51 to 1·81)
	£35,000 ≥	254 (54)	220 (46)	Reference	Reference	285 (86)	47 (14)	Reference	Reference
Employment status[1]	Working	362 (52)	337 (48)	**1·95* (1·25 to 3·05)**	**1·94* (1·16 to 3·24)**	435 (86)	70 (14)	**9·33* (1·27 to 68·47)**	**8·64* (1·13 to 65·96)**
	Not working	67 (68)	32 (32)	Reference	Reference	58 (98)	1 (2)	Reference	Reference
Education level	≤ A-level	179 (63)	105 (37)	**0·56** (0·41 to 0·75)**	**0·59* (0·42 to 0·84)**	227 (88)	31 (12)	0·91 (0·55 to 1·51)	1·27 (0·71 to 2·26)
	Degree ≥	248 (49)	261 (51)	Reference	Reference	267 (87)	40 (13)	Reference	Reference
Marital status	Living alone	49 (48)	54 (52)	1·34 (0·88 to 2·02)	1·30 (0·82 to 2·06)	75 (88)	10 (12)	0·91 (0·45 to 1·85)	0·92 (0·41 to 2·03)
	Married/cohabiting	383 (55)	316 (45)	Reference	Reference	422 (87)	62 (13)	Reference	Reference
Ethnicity	White	355 (53)	316 (47)	1·23 (0·84 to 1·12)	**1·66* (1·07 to 2·58)**	450 (87)	65 (13)	1·04 (0·42 to 2·53)	1·47 (0·53 to 4·13)
	BAME	72 (58)	52 (42)	Reference	Reference	43 (78)	12 (22)	Reference	Reference
Child gender	Boy	230 (51·5)	217 (48·5)	1·25 (0·94 to 1·65)	1·15 (0·85 to 1·56)	244 (87)	36 (13)	1·04 (0·63 to 1·70)	0·96 (0·56 to 1·66)
	Girl	202 (57)	153 (43)	Reference	Reference	253 (87·5)	36 (12·5)	Reference	Reference
School year	Early Years	77 (59)	53 (41)	0·74 (0·50 to 1·10)	0·79 (0·51 to 1·23)	***	***	***	***
	Key Stage 1	157 (54)	132 (46)	0·90 (0·66 to 1·22)	0·88 (0·63 to 1·24)	30 (86)	5 (14)	2·10 (0·57 to 7·81)	1·52 (0·37 to 6·30)
	Key Stage 2	198 (52)	185 (48)	Reference	Reference	120 (86)	20 (15)	2·10 (0·75 to 5·86)	1·45 (0·47 to 4·44)
	Key Stage 3	***	***	***	***	152 (87)	23 (13)	1·91 (0·69 to 5·24)	1·58 (0·55 to 4·55)
	Key Stage 4	***	***	***	***	132 (87)	19 (13)	1·81 (0·65 to 5·08)	1·44 (0·49 to 4·24)
	Years 12 & 13	***	***	***	***	63 (93)	5 (7)	Reference	Reference
School type	Fee-paying	35 (33)	71 (67)	**2·68** (1·74 to 4·13)**	**2·50** (1·56 to 4·00)**	30 (77)	9 (23)	**2·20* (1·00 to 4·85)**	2·04 (0·82 to 5·07)
	State-funded	395 (57)	299 (43)	Reference	Reference	462 (88)	63 (12)	Reference	Reference
Child has SEN	Yes	22 (31)	48 (69)	**2·78** (1·64 to 4·70)**	**2·30* (1·27 to 4·17)**	28 (61)	18 (39)	**5·74** (2·97 to 11·08)**	**7·92** (3·59 to 17·46)**
	No	406 (56)	319 (44)	Reference	Reference	464 (90)	52 (10)	Reference	Reference
Low child well-being	Yes	92 (42)	124 (57)	**1·86** (1·36 to 2·55)**	**1·47* (1·04 to 2·07)**	127 (82)	28 (18)	**1·85* (1·11 to 3·10)**	**1·78* (1·00 to 3·21)**

Confidential: For Review Only

	No	340 (58)	246 (42)	Reference	Reference	370 (89)	44 (11)	Reference	Reference
Child vulnerable COVID-19	Yes	28 (39)	44 (61)	**2·00* (1·21 to 3·28)**	**1·76* (1·00 to 3·10)**	33 (82·5)	7 (17·5)	1·49 (0·63 to 3·50)	0·93 (0·33 to 2·63)
	No	401 (56)	316 (44)	Reference	Reference	455 (87·5)	65 (12·5)	Reference	Reference
Household vulnerable COVID-19	Yes	115 (60)	78 (40)	0·78 (0·56 to 1·08)	0·74 (0·51 to 1·06)	134 (88)	18 (12)	0·90 (0·50 to 1·60)	0·56 (0·44 to 1·67)
	No	295 (53)	258 (47)	Reference	Reference	314 (87)	47 (13)	Reference	Reference
Child COVID-19 symptoms	Yes	15 (24)	47 (76)	**5·05** (2·22 to 7·36)**	**3·02* (1·49 to 6·15)**	11 (58)	8 (42)	**5·52** (2·14 to 14·24)**	**5·25* (1·58 to 17·41)**
	No	417 (56)	323 (44)	Reference	Reference	486 (88)	64 (12)	Reference	Reference
Household COVID-19 symptoms	Yes	9 (17)	43 (83)	**6·18** (2·97 to 12·86)**	**6·04** (2·62 to 13·91)**	13 (65)	7 (35)	**4·00* (1·54 to 10·42)**	**4·11* (1·26 to 13·44)**
	No	423 (56)	327 (43)	Reference	Reference	484 (88)	65 (12)	Reference	Reference
Child has had or currently has COVID-19	Yes	36 (24)	112 (76)	**4·78** (3·18 to 7·17)**	**4·03** (2·57 to 6·30)**	40 (67)	20 (33)	**4·40** (2·40 to 8·08)**	**5·20** (2·48 to 10·93)**
	No	396 (61)	258 (37)	Reference	Reference	457 (90)	52 (10)	Reference	Reference
Someone over 70 years	Yes	11 (26)	32 (74)	**3·62** (1·80 to 7·30)**	**3·19* (1·45 to 7·05)**	8 (57)	6 (43)	**5·56* (1·87 to 16·52)**	**3·49* (0·98 to 12·46)**
	No	421 (55·5)	338 (44·5)	Reference	Reference	489 (88)	66 (12)	Reference	Reference

* *p*≤ *·05* and formatted bold

** *p*≤ *·001* and formatted bold

*** data not applicable for the sample

† Adjusting for participant gender, age, region, household income, employment status, education level, marital status, participant ethnicity, and the child's gender, school year and school-type.

[1] Working includes students and volunteers

Decimal places have been used for rounding errors

Confidential: For Review Only

DISCUSSION

Most children eligible to attend school did not attend one week after schools in England began to re-open to more children.[25] Worryingly, we observed patterns that seemed likely to entrench existing educational inequalities. Children from households where parents have lower education achievements, BAME households and households in the North of England, and state-funded being least likely to attend school.

Our results suggest that several broad areas determined attendance at school. First, risk perceptions were crucial. The response “it is too risky” featured highly in parental reasons for not sending children to school. Secondly, children were also more likely to attend school when parents thought their child had had COVID-19 which could indicate the belief their child is immune to further infection.[26] Unexpectedly, children were more likely to attend school when they had a health condition that made them vulnerable to COVID-19 and when a person over 70 years was living in the household. We speculate that this may be because these circumstances affect the parent’s ability to look after the child at home.

Poorer perceived child well-being was associated with them being more likely to attend school. This finding may reflect parental desire to improve their child’s well-being which may have been impacted by the school closure.[27] This was also apparent in reasons parents gave for sending their child to school. As expected,[14] concerns about education also featured highly as a reason for attendance, while perceptions that schools could not provide good quality education or that some lessons were not as important were cited as reasons for absence or partial attendance.

Despite most children of key workers not attending school, needing to work was the most reported reason for school attendance and the school not opening or asking the child not to attend were the most commonly reported reasons for the child not being in school. This suggests that work commitments were the main driver for this group.

One notable finding was that children who had symptoms of COVID-19 in the past seven days, or whose household members had these symptoms in the past 14 days were significantly more likely to have gone to school. We do not know if the child attended school whilst having symptoms or when symptoms were present in the household (against self-isolation guidance)[28]. However, given that school closure is specifically designed to reduce the transmission of respiratory infections in general, it is also possible that this reflects the re-emergence of transmission of upper respiratory tract infections, or COVID-19 specifically, within the school environment.[29] It may also reflect increased anxiety or awareness among parents around COVID-19 symptoms, resulting in higher symptom detection and reporting.

LIMITATIONS

Several limitations should be borne in mind for this study. First the cross-sectional nature of this study limits our ability to draw causal findings. Second, the RCADS subscale was used to indicate low well-being but is currently not validated for children under eight years.[30] Third, online polls can be unrepresentative, and lead to response, and self-reporting bias.[31] However, in line with the reasoning relating to the use of non-probability samples in social sciences, [32] we assume that the associations within our data do generalise to the wider population. Fourth, we ran many analyses raising the possibility of Type 1 errors. While we have provided Bonferroni corrections in the tables for readers who wish to correct this, this correction in turn may be overly conservative.

CONCLUSION

Our findings support previous research by suggesting that during an infectious disease outbreak, parents' decision to send their child to school was impacted by the risk of disease (COVID-19), child's education and well-being. Furthermore, without reassuring parents in these three areas and encouraging them to send their children to school health inequalities are likely to be increased.

Contributors

All authors contributed to the conceptualisation of the study and approved the final draft. LW designed the survey, analysed the data and drafted the manuscript. LES designed the survey, analysed the data and edited the manuscript. RKW edited the manuscript. RA designed the survey and edited the manuscript. AR designed the survey and edited the manuscript. SW edited the manuscript. GJR designed the survey, analysed the data and edited the manuscript.

Declarations of interests

We declare no competing interests.

Acknowledgments

This study was funded by the Economic and Social Research Council and the National Institute for Health Research Health Protection Research Unit (NIHR HPRU) [grant number NIHR200890] in Emergency Preparedness and Response, a partnership between Public Health England, King's College London and the University of East Anglia. The views expressed are those of the authors and not necessarily those of the NIHR, Public Health England or the Department of Health and Social Care.

Role of the funding source

Confidential: For Review Only

The funders had no role in study design, data collection, data analysis, data interpretation, or writing of the manuscript. The corresponding author had full access to all the data and had final responsibility for the decision to submit for publication.

Confidential: For Review Only

REFERENCES

1 Prime Minister B Johnson. PM statement on coronavirus: 18 March 2020. Available at: https://www.gov.uk/government/speeches/pm-statement-on-coronavirus-18-march-2020?utm_source=407969fc-4de1-41c8-9571-f6b6ad35efae&utm_medium=email&utm_campaign=govuk-notifications&utm_content=immediate Accessed 11 June 2020.
2 Government. H. Children of critical workers and vulnerable children who can access schools or educational settings. Available at: https://www.gov.uk/government/publications/coronavirus-covid-19-maintaining-educational-provision/guidance-for-schools-colleges-and-local-authorities-on-maintaining-educational-provision Accessed 16 March 2021.
3 Prime Minister B Johnson. PM confirms schools, colleges and nurseries on track to begin phased reopening. Available at: https://www.gov.uk/government/news/pm-confirms-schools-colleges-and-nurseries-on-track-to-begin-phased-reopening Accessed 11 June 2020.
4 HM Government. School Attendance. Available at: https://www.gov.uk/government/publications/school-attendance Accessed 8 July 2020.
5 Viner RM, Russell SJ, Croker H, et al. School closure and management practices during coronavirus outbreaks including COVID-19: a rapid systematic review. *The Lancet Child & Adolescent Health*. 2020;4(5):397-404 DOI: 10.1016/s2352-4642(20)30095-x
6 Keogh-Brown MR, Jensen HT, Edmunds WJ, Smith RD. The impact of Covid-19, associated behaviours and policies on the UK economy: A computable general equilibrium model. *SSM - Population Health*. 2020;[in press]:100651 DOI: 10.1016/j.ssmph.2020.100651
7 Boast A, Munro A, Goldstein H. An evidence summary of Paediatric COVID-19 literature. Available at: https://dontforgetthebubbles.com/evidence-summary-paediatric-covid-19-literature/ Accessed 8 July 2020.
8 Zhu Y, Bloxham C, K H, et al. Children are unlikley to have been the primary source of household SARS-CoV-2 infections. 2020; DOI: 10.1101/2020.03.26.20044826v1
9 World Health Organization. Report of the WHO-China joint mission on coronavirus disease 2019 (COVID-19). Available at: https://www.who.int/publications/i/item/report-of-the-who-china-joint-mission-on-coronavirus-disease-2019-(covid-19) Accessed 8 July 2020.
10 Heavey L, Casey G, Kelly C, et al. No evidence of secondary transmission of COVID-19 from children attending school in Ireland, 2020. *Eurosurveillance*. 2020;25(21) DOI: 10.2807/1560-7917.es.2020.25.21.2000903
11 Otte Im Kampe E, Lehfeld A-S, Buda S, et al. Surveillance of COVID-19 school outbreaks, Germany, March to August 2020. *Eurosurveillance*. 2020;25(38) DOI: 10.2807/1560-7917.es.2020.25.38.2001645
12 England. PH. SARS-CoV-2 infection and transmission in educational settings. Available at: https://www.gov.uk/government/publications/sars-cov-2-infection-and-transmission-in-educational-settings Accessed 22 October 2020.
13 Royal College of Paediatrics and Child Health. COVID_19 - talking to children and families about returning to school: guiding principles. Available at: https://www.rcpch.ac.uk/resources/covid-19-talking-children-families-about-returning-school-guiding-principles Accessed 9 July 2020.

Confidential: For Review Only

14 Brooks SK, Smith LE, Webster RK, et al. The impact of unplanned school closure on children's social contact: rapid evidence review. *Euro Surveill*. 2020;25(13) DOI: 10.2807/1560-7917.ES.2020.25.13.2000188
15 Basurto-Dávila R, Garza R, Meltzer MI, et al. Household economic impact and attitudes toward school closures in two cities in Argentina during the 2009 influenza A (H1N1) pandemic. *Influenza and Other Respiratory Viruses*. 2013;7(6):1308-15 DOI: 10.1111/irv.12054
16 Effler PV, Carcione D, Giele C, et al. Household responses to pandemic (H1N1) 2009-related school closures, Perth, Western Australia. *Emerg Infect Dis*. 2010;16(2):205-11 DOI: 10.3201/eid1602.091372
17 van Gemert C, McBryde ES, Bergeri I, et al. Description of social contacts among student cases of pandemic influenza during the containment phase, Melbourne, Australia, 2009. *Western Pac Surveill Response J*. 2018;9(5 Suppl 1):27-34 DOI: 10.5365/wpsar.2018.9.5.003
18 Lee J. Mental health effects of school closures during COVID-19. *The Lancet Child & Adolescent Health*. 2020;4(6):421 DOI: 10.1016/s2352-4642(20)30109-7
19 Smith LE, Woodland L, Amlôt R, et al. A cross-sectional survey of parental perceptions of COVID-19 related hygiene measures within schools and adherence to social distancing in journeys to and from school. *BMJ Paediatrics Open*. 2020;4(1) DOI: 10.1136/bmjpo-2020-000825
20 Research. B. BMG About Us. Available at: https://www.bmgresearch.co.uk/bmg/about-us/ Accessed 16 March 2021.
21 HM Government. Schools, pupils and their characteristics: Academic Year 2019/20. Available at: https://explore-education-statistics.service.gov.uk/find-statistics/school-pupils-and-their-characteristics Accessed 25 June 2020.
22 Chorpita BF, Yim L, Moffitt C, et al. Assessment of symptoms of DSM-IV anxiety and depression in children: a revised child anxiety and depression scale. *Behaviour Research and Therapy*. 2000;38(8):835-55 DOI: 10.1016/s0005-7967(99)00130-8
23 Chorpita BF. Child FIRST - Focus on innovation and redesign in systems and treatment. Available at: https://www.childfirst.ucla.edu/resources/ Accessed 6 July 2020.
24 IBM Corp.2019. IBM SPSS Statistics for Windows, 26.0 Armonk, NY.
25 HM Government. attendance in education and early years settings during the coronavirus (COVID-19) outbreak. Available at: https://www.gov.uk/government/publications/coronavirus-covid-19-attendance-in-education-and-early-years-settings?utm_source=efcd291f-83ee-4d73-860a-7e823b3c0017&utm_medium=email&utm_campaign=govuk-notifications&utm_content=immediate Accessed 9 June 2020.
26 Smith LE, Mottershaw AL, Egan M, et al. The impact of believing you have had COVID-19 on self-reported behaviour: Cross-sectional survey. *PLOS ONE*. 2020;15(11):e0240399 DOI: 10.1371/journal.pone.0240399
27 Idoiaga M, Berasategi S, Dosil S, Eiguren M. Struggling to breathe: a qualitative study of children's wellbeing during lockdown in Spain. *Psychology & Health*. 2020:1-16 DOI: 10.1080/08870446.2020.1804570
28 HM Government. Protective measures for out-of-school settings during the coronavirus (COVID-19) outbreak. Available at: https://www.gov.uk/government/publications/protective-measures-for-holiday-or-after-school-clubs-and-other-out-of-school-settings-for-children-during-the-coronavirus-covid-19-outbreak/protective-measures-for-out-of-school-settings-during-the-coronavirus-covid-19-outbreak Accessed 8 July 2020.

Confidential: For Review Only

29 Panovska-Griffiths J, Kerr CC, Stuart RM, et al. Determining the optimal strategy for reopening schools, the impact of test and trace interventions, and the risk of occurrence of a second COVID-19 epidemic wave in the UK: a modelling study. *The Lancet Child & Adolescent Health*. 2020; DOI: 10.1016/s2352-4642(20)30250-9
30 Ebesutani C, Chorpita BF, Higa-Mcmillan CK, et al. A Psychometric Analysis of the Revised Child Anxiety and Depression Scales—Parent Version in a School Sample. *Journal of Abnormal Child Psychology*. 2011;39(2):173-85 DOI: 10.1007/s10802-010-9460-8
31 Epstein WM. Response bias in opinion polls and American social welfare. *The Social Science Journal*. 2006;43(1):99-110 DOI: 10.1016/j.soscij.2005.12.010
32 Kohler U. Possible Uses of Nonprobability Sampling for the Social Sciences. *Survey Methods: Insights from the Field*. Available at: https://surveyinsights.org/?p=10981 Accessed 29 July 2020.

Confidential: For Review Only

SUPPLEMENTARY MATERIAL

Table 1: Table of frequencies to show reasons children did not attend school.

Reason	Children in School Years (n=432, %)	Children of Key Workers (n=497, %)
I think it is too risky for my child to attend school at the moment	223 (52%)	109 (22%)
The school is not open	140 (32%)	259 (52%)
I have another child who can't go to school	67 (15·5%)	24 (5%)
Being at school will be stressful for my child	59 (14%)	23 (5%)
I think they will get a better education at home than at school at the moment	57 (13%)	25 (5%)
The school has asked my child not to attend	51 (12%)	117 (23·5%)
Someone in my household is clinically vulnerable to coronavirus	45 (10%)	24 (5%)
My child doesn't want to go	44 (10%)	29 (6%)
There's no point, schools will shut again soon anyway	37 (9%)	13 (3%)
My child's friends will not be at school	28 (6·5%)	19 (4%)
Other reason	28 (6·5%)	65 (13%)
I think friends, family or other parents will judge me if I send my child to school	12 (3%)	4 (1%)
I can't arrange transport to get them to school	6 (1%)	10 (2%)
Someone else at the school developed coronavirus symptoms (cough or fever, or change in sense of taste or smell)	6 (1%)	4 (1%)
My child developed symptoms of a different illness	3 (1%)	1 (0%)
They have developed coronavirus symptoms (cough or fever, or change in sense of taste or smell)	2 (0·5%)	2 (0%)
Someone else in the family developed coronavirus symptoms (cough or fever, or change in sense of taste or smell)	0 (0%)	3 (1%)

Table 2: Table of frequencies to show reasons children attended school.

Reason	Children in School Years (n=370, %)	Children of Key Workers (n=72, %)
My child's education will benefit from being at school	208 (56%)	30 (42%)
My child wants to go to school	200 (54%)	35 (49%)
My child will benefit from seeing their friends	187 (50·5%)	27 (37·5%)
I need to work	120 (32%)	40 (56%)
I don't believe my child will be at risk at school	96 (26%)	17 (24%)
Having my child at home is a strain	57 (15%)	10 (14%)
It is compulsory for my child to attend	50 (13.5%)	9 (12.5%)
It reduces costs at home	48 (13%)	7 (10%)
I think friends, family or other parents will judge me if I don't send my child to school	43 (12%)	11 (15%)
I have non-work commitments I need to meet	21 (6%)	4 (6%)
Other reason	9 (2%)	3 (4%)

Table 3: Table of frequencies to show reasons children attended school part-time (less than five days).

Reason	Children in School Years (n=204, %)	Children of Key Workers (n=39, %)
The school only offers for them to be in part-time	80 (39%)	16 (41%)
It is less risky for them to be in part-time	40 (20%)	7 (18%)

Confidential: For Review Only

I am only sending them in on days where the lessons are important	28 (14%)	8 (20·5%)
Other reason	27 (13%)	3 (8%)
I am using it as childcare and I only need them in part time	24 (12%)	6 (15%)
I am only sending them in on days when I can arrange transport	23 (11%)	4 (10%)
Someone else at the school developed coronavirus symptoms (cough or fever, or change in sense of taste or smell)	14 (7%)	1 (3%)
I started sending them in, but have now changed my mind	13 (6%)	2 (5%)
They have developed coronavirus symptoms (cough or fever, or change in sense of taste or smell)	13 (6%)	1 (3%)
Someone else in the family developed coronavirus symptoms (cough or fever, or change in sense of taste or smell)	12 (6%)	0 (0%)
I am only sending them in on days when their friends are in	11 (5%)	6 (15%)
My child developed symptoms of a different illness	9 (4%)	0 (0%)
They only live with me part time	6 (3%)	1 (3%)

Confidential: For Review Only

Full Survey Material

D1. Please type your age on your last birthday: [number]

Screenout if D1 = <18

Q1. Which country do you live in? [single code]

a. England
b. Scotland
c. Wales
d. Northern Ireland
e. Outside of the UK

Screenout if Q1 = b-e

Q2. Which region do you live in? [single code]

a. East Midlands
b. East of England
c. London
d. North East
e. North West
f. South East
g. South West
h. West Midlands
i. Yorkshire and the Humber

Q3. Are you the parent or guardian of a child who usually attends school (that is, before the coronavirus outbreak occurred) and who lives with you? [single code]

a. Yes
b. No

Screenout if Q3 = b

Q4. Which of the following age groups do your children who live with you fall into? Please tick any that apply. [multi code]

a. 0-3
b. 4-11
c. 12-18
d. None of the above [exclusive]

Screenout if b OR c not selected

Q5. The Government has categorised some people as "critical workers" or "key workers." For example, this includes people working in health and social care, education, key public services, transport, food distribution and other jobs. As far as you know, are you, or a spouse/partner, a "critical" or "key" worker? Please tick any that apply. [multi code]

a. Yes, me
b. Yes, spouse/partner
c. No [exclusive]
d. Don't know [exclusive]

IF Q4 = b

Q6. You stated that you have at least one child aged 4-11 who lives with you. Are any of your children who live with you in the following school years? Please tick any that apply. [multi code]

a. Reception
b. Year 1
c. Year 6

Confidential: For Review Only

d. I have no children in the school years listed above [exclusive]

IF 2 or more options are selected at Q6

Q7. Thinking only about your children in Reception, Year 1 or Year 6, who live with you, which child had the most recent birthday? If the most recent birthday was shared by more than one child (e.g. twins, or children born on the same day in different years), please answer the following questions thinking about one of these children only. [single code]

a. My child in Reception
b. My child in Year 1
c. My child in Year 6

ONLY SHOW OPTIONS SELECTED AT Q6

IF Q6 = a-c

Q8. Can you please tell us how old that child is? [single code]

a. 4
b. 5
c. 6
d. 7
e. 8
f. 9
g. 10
h. 11

IF Q6 = a-c

Q9. Can you please tell us if that child is a: [single code]

a. Boy
b. Girl

IF Q6 = a-c

Q10. Can you please tell us the type of school that child usually attends? [single code]

a. Fee-paying
b. State-funded school
c. Don't know

IF Q6 = a-c

Q11A. Thinking about that child, have they attended school at all in the past 7 days? [single code]

a. Yes, on 5 days
b. Yes, on 1 to 4 days
c. No
d. Don't know

IF Q11A = a/b

Q12A. You said that your child has attended school on at least one day in the past week. For which reasons did you send your child to school? Please tick any that apply [multi code]:

RANDOMISE ORDER OF STATEMENTS, anchor other to bottom

a) It is compulsory for my child to attend
b) I need to work
c) I have non-work commitments I need to meet
d) My child's education will benefit from being at school
e) My child will benefit from seeing their friends
f) Having my child at home is a strain
g) I don't believe my child will be at risk at school

Confidential: For Review Only

h) My child wants to go to school
i) I think friends, family or other parents will judge me if I don't send my child to school
j) It reduces costs at home
k) Other reason [write in]

IF Q11A = b

Q13A. You said that your child has not attended school every day in the past week. For which reasons are they attending only part-time? Please tick any that apply [multi code]:

RANDOMISE ORDER OF STATEMENTS, anchor other to bottom

a) I am using it as childcare and I only need them in part time
b) It is less risky for them to be in part-time
c) The school only offers for them to be in part-time
d) I am only sending them in on days where the lessons are important
e) I am only sending them in on days when their friends are in
f) I am only sending them in on days when I can arrange transport
g) They only live with me part time
h) I started sending them in, but have now changed my mind
i) They have developed coronavirus symptoms (cough or fever, or change in sense of taste or smell)
j) My child developed symptoms of a different illness
k) Someone else in the family developed coronavirus symptoms (cough or fever, or change in sense of taste or smell)
l) Someone else at the school developed coronavirus symptoms (cough or fever, or change in sense of taste or smell)
m) Other reason [write in]

IF Q11A = c

Q14A. You said that your child has not attended school in the past week. For which reasons did you not send your child to school? Please tick any that apply [multi code]:

RANDOMISE ORDER OF STATEMENTS, anchor other to bottom

a) Someone in my household is clinically vulnerable to coronavirus
b) I think it is too risky for my child to attend school at the moment
c) The school is not open
d) The school has asked my child not to attend
e) My child doesn't want to go
f) I think friends, family or other parents will judge me if I send my child to school
g) I think they will get a better education at home than at school at the moment
h) Being at school will be stressful for my child
i) My child's friends will not be at school
j) I have another child who can't go to school
k) I can't arrange transport to get them to school
l) There's no point, schools will shut again soon anyway
m) They have developed coronavirus symptoms (cough or fever, or change in sense of taste or smell)
n) My child developed symptoms of a different illness
o) Someone else in the family developed coronavirus symptoms (cough or fever, or change in sense of taste or smell)
p) Someone else at the school developed coronavirus symptoms (cough or fever, or change in sense of taste or smell)
q) Other reason [write in]

IF Q11A = a/b

Q15A. In this question we are interested in things that happened on the most recent day that your child went to school. Please remember that this survey is anonymous – please be honest in your answers. Please tick any that apply. [multi code]

RANDOMISE ORDER OF STATEMENTS, anchor none of these to bottom

Confidential: For Review Only

a) Either on the way to or from school, or at the school gates, I had physical contact with someone that I don’t live with
b) Either on the way to or from school, or at the school gates, I was within 1 metre of someone that I don’t live with for 1 minute or longer
c) Either on the way to or from school, or at the school gates, I was between 1 and 2 metres of someone that I don’t live with for 15 minutes or longer
d) My child used public transport to get to or from school.
e) My child shared a lift in a car with another family to get to or from school.
f) I gave a lift in a car to a child from another family to get them to or from school.
g) My child washed their hands as soon as they got home from school.
h) My child had a shower or bath as soon as they got home from school.
i) I washed my child’s clothes after they got home from school.
j) None of these [exclusive]

IF Q11A = a/b

Q16A. Thinking about the facilities or procedures at your child’s school. Which of the following, if any, are actually happening as far as you are aware?

Please tick all that apply [multi code]

RANDOMISE ORDER OF STATEMENTS, anchor none of these to bottom

a) There are hand washing facilities or hand gel dispensers at the entrance to the school that are working
b) There are hand washing facilities or hand gel dispensers at the entrance to the classrooms that are working
c) Children’s hand washing or hand gel use is being monitored at school
d) My child’s class sizes is now 15 or fewer
e) Children need to take in their own food and snacks
f) Children are having their temperature checked on the way in
g) The school has used markings or barriers to help children keep their distance from each other
h) None of these are happening at my child’s school [exclusive]

Q17. For the next series of questions, we would like you to think about one of your children who usually goes to school (that is, before the coronavirus outbreak occurred). If you have more than one child who usually went to school, then please answer about your child with the most recent birthday. If the most recent birthday was shared by more than one child (e.g. twins, or children born on the same day in different years), please answer the following questions thinking about one of these children only.

Firstly, can you tell us the first name of your child. This is just so we can refer to them throughout the survey. You can give a fake name if you would prefer. [open]

____

Q18. How old is [CHILD]? [single code]

a. 4
b. 5
c. 6
d. 7
e. 8
f. 9
g. 10
h. 11
i. 12
j. 13
k. 14
l. 15
m. 16
n. 17
o. 18

Confidential: For Review Only

Q19. What year is [CHILD] in at school [single code]

a. Reception
b. Year 1
c. Year 2
d. Year 3
e. Year 4
f. Year 5
g. Year 6
h. Year 7
i. Year 8
j. Year 9
k. Year 10
l. Year 11
m. Year 12
n. Year 13

Q20. Is [CHILD] a: [single code]

a. Boy
b. Girl

Q21. What type of school does [CHILD] normally attend? [single code]

a. Fee-paying
b. State-funded school
c. Don't know

If Q5 = a/b AND [Q4 ≠ b or Q6 = d)

Q11B. Has [CHILD] attended school at all in the past 7 days? [single code]

a. Yes, on 5 days
b. Yes, on 1 to 4 days
c. No
d. Don't know

IF Q11B = a/b

Q12B. You said that [CHILD] has attended school on at least one day in the past week. For which reasons did you send [CHILD] to school? Please tick any that apply [multi code]:

RANDOMISE ORDER OF STATEMENTS, anchor other to bottom

a) It is compulsory for my child to attend
b) I need to work
c) I have non-work commitments I need to meet
d) My child's education will benefit from being at school
e) My child will benefit from seeing their friends
f) Having my child at home is a strain
g) I don't believe my child will be at risk at school
h) My child wants to go to school
i) I think friends, family or other parents will judge me if I don't send my child to school
j) It reduces costs at home
k) Other reason [write in]

IF Q11B = b

Q13B. You said that [CHILD] has not attended school every day in the past week. For which reasons is [CHILD] attending only part-time? Please tick any that apply [multi code]:

RANDOMISE ORDER OF STATEMENTS, anchor other to bottom

a) I am using it as childcare and I only need them in part time
b) It is less risky for them to be in part-time

Confidential: For Review Only

c) The school only offers for them to be in part-time
d) I am only sending them in on days where the lessons are important
e) I am only sending them in on days when their friends are in
f) I am only sending them in on days when I can arrange transport
g) They only live with me part time
h) I started sending them in, but have now changed my mind
i) They have developed coronavirus symptoms (cough or fever, or change in sense of taste or smell)
j) My child developed symptoms of a different illness
k) Someone else in the family developed coronavirus symptoms (cough or fever, or change in sense of taste or smell)
l) Someone else at the school developed coronavirus symptoms (cough or fever, or change in sense of taste or smell)
m) Other reason [write in]

IF Q11B = c

Q14B. You said that [CHILD] has not attended school in the past week. For which reasons did you not send [CHILD] to school? Please tick any that apply [multi code]:

RANDOMISE ORDER OF STATEMENTS, anchor other to bottom

a) Someone in my household is clinically vulnerable to coronavirus
b) I think it is too risky for my child to attend school at the moment
c) The school is not open
d) The school has asked my child not to attend
e) My child doesn't want to go
f) I think friends, family or other parents will judge me if I send my child to school
g) I think they will get a better education at home than at school at the moment
h) Being at school will be stressful for my child
i) My child's friends will not be at school
j) I have another child who can't go to school
k) I can't arrange transport to get them to school
l) There's no point, schools will shut again soon anyway
m) They have developed coronavirus symptoms (cough or fever, or change in sense of taste or smell)
n) My child developed symptoms of a different illness
o) Someone else in the family developed coronavirus symptoms (cough or fever, or change in sense of taste or smell)
p) Someone else at the school developed coronavirus symptoms (cough or fever, or change in sense of taste or smell)
q) Other reason [write in]

IF Q11B = a/b

Q15B. In this question we are interested in things that happened on the most recent day that [CHILD] went to school. Please remember that this survey is anonymous – please be honest in your answers. Please tick any that apply. [multi code]

RANDOMISE ORDER OF STATEMENTS, anchor none of these to bottom

a) Either on the way to or from school, or at the school gates, I had physical contact with someone that I don't live with
b) Either on the way to or from school, or at the school gates, I was within 1 metre of someone that I don't live with for 1 minute or longer
c) Either on the way to or from school, or at the school gates, I was between 1 and 2 metres of someone that I don't live with for 15 minutes or longer
d) My child used public transport to get to or from school.
e) My child shared a lift in a car with another family to get to or from school.
f) I gave a lift in a car to a child from another family to get them to or from school.
g) My child washed their hands as soon as they got home from school.
h) My child had a shower or bath as soon as they got home from school.
i) I washed my child's clothes after they got home from school.
j) None of these [exclusive]

Confidential: For Review Only

IF Q11B = a/b

Q16B. Thinking about the facilities or procedures at [CHILD]'s school. Which of the following, if any, are actually happening as far as you are aware? Please tick any that apply. [multi code]

RANDOMISE ORDER OF STATEMENTS, anchor none of these to bottom

a) There are hand washing facilities or hand gel dispensers at the entrance to the school that are working
b) There are hand washing facilities or hand gel dispensers at the entrance to the classrooms that are working
c) Children's hand washing or hand gel use is being monitored at school
d) My child's class size is now 15 or fewer
e) Children need to take in their own food and snacks
f) Children are having their temperature checked on the way in
g) The school has used markings or barriers to help children keep their distance from each other
h) None of these are happening at [CHILD]'s school [exclusive]

Q22. Does [CHILD] have special educational needs? [singe code]

- Yes
- No
- Don't know / prefer not to say

Q23. In the past 7 days, how many times, if at all, has [CHILD] left your home for each of the following reasons? [multi code]

a) To go to the shops for groceries, toiletries or medicines
b) To go to the shops for other items
c) For exercise
d) For a medical need (e.g. an outpatient appointment)
e) To go to school
f) To provide help to someone else
g) To meet friends
h) To meet family members who don't live with you
i) For another reason

Q24. In the past 7 days, have you done the following, because of the risk from coronavirus:

SCALE

- Yes
- No

STATEMENTS – RANDOMISE

a. Washed your hands thoroughly and regularly
b. Stayed 2m (3 steps) away from people you do not live with when outside your home
c. Washed your clothes when you have returned home
d. Washed [CHILD]'s clothes when she/ he has returned home
e. Used tissues or clothing to touch door handles or other objects when outside
f. Cleaned or disinfected items you have brought into the home, such as groceries or parcels
g. Left items that you have brought into the home for 24 hours or more before using them, to kill any virus on them
h. Covered your face or used a face mask when out and about
i. Covered [CHILD's] face or given her/him a face mask when out and about
j. Worn protective gloves when out and about
k. Made [CHILD] wear protective gloves when out and about

Confidential: For Review Only

Q25. We are interested in how many people [CHILD] has had close contact with in the past 24 hours. By close contact we mean closer than 2 meters, for fifteen minutes or more. Please remember, this survey is anonymous so please be honest.

In the past 24 hours, how many people in the following categories has [CHILD] had close contact with: [multi code]

a. Someone [CHILD] lives with
b. Friends or other children who [CHILD] does not live with
c. A family member aged under 70 who [CHILD] does not live with
d. A family member aged over 70 who [CHILD] does not live with
e. A babysitter, nanny or childminder
f. Other children, not already reported above
g. Other adults, not already reported above

Q26. Did [CHILD] attend school in the past 24 hours? [single code]

a. Yes
b. No

RANDOMISE ORDER OF Q27a AND Q27b SHOWN TO EACH RESPONDENT

Q27a. How much, if at all, do you agree or disagree with the following statements:

STATEMENTS (randomise order):

a) If [CHILD] goes out, she/he is likely to catch coronavirus
b) If [CHILD] goes out, she/he is likely to bring coronavirus back into our home
c) [CHILD] is keeping up with her/his schoolwork
d) I feel confident helping [CHILD] with her/his schoolwork
e) I feel supported by [CHILD's] school
f) I have access to all the resources that [CHILD] needs to do her/his schoolwork
g) During lockdown, [CHILD] has learned about important things she/he wouldn't normally learn at school.
h) In the past 7 days, [CHILD] has been bored

SCALE

- Strongly agree
- Tend to agree
- Neither agree nor disagree
- Tend to disagree
- Strongly disagree
- Not applicable

Q27b. How much, if at all, do you agree or disagree with the following statements:

STATEMENTS (randomise order):

a) In the past 7 days, my household has had a regular structure to the day
b) In the past 7 days, [CHILD] has kept in touch with her/his friends
c) [CHILD] is worried about coronavirus
d) In the past 7 days, [CHILD] has felt upset about not seeing other family members who do not live with us
e) In the past 7 days, I have found it hard to keep up with work or other important commitments
f) In the past 7 days, people in my household have been getting along well
g) I am worried about the financial impact of lockdown measures
h) Before the school closures, [CHILD] had extra support at school

SCALE

- Strongly agree
- Tend to agree

Confidential: For Review Only

- Neither agree nor disagree
- Tend to disagree
- Strongly disagree
- Not applicable

Q28. To what extent, if at all, do you agree or disagree with the following statements?

RANDOMISE ORDER OF STATEMENTS

a) If it were possible, I would feel comfortable sending [CHILD] to school next week.
b) If it were possible, I would want to send [CHILD] to school next week.
c) There is still too much coronavirus around for schools to be able to reopen safely
d) When [CHILD's] school fully reopens, some parents will send their children to school, even if they have symptoms of coronavirus
e) It is impossible for children to maintain 'social distancing' at school
f) Children will not wash their hands properly at school
g) Staff will not wash their hands properly at school
h) Schools will not be able to provide good quality education at the moment
i) School will not be an enjoyable place for [CHILD] to be at the moment

SCALE

- Strongly agree
- Tend to agree
- Neither agree nor disagree
- Tend to disagree
- Strongly disagree
- Not applicable

Q29A. Has [CHILD] had any of the following symptoms in the past 7 days? Please tick any that apply, no matter where you think the symptoms came from, and even if the symptoms were mild. [multi code]

RANDOMISE ORDER OF STATEMENTS, anchor "none of these" and "don't know" to bottom

- New, continuous cough
- High temperature / fever
- Runny nose
- Diarrhoea
- Nausea / feeling sick
- Vomiting
- Sneezing
- Loss of appetite
- Loss of sense of smell (fully or partial)
- Loss of taste
- None of these
- Don't know

Q29B. Have you, or anyone else in your household (excluding [CHILD]) had any of the following symptoms in the past 14 days? Please tick any that apply, no matter where you think the symptoms came from, and even if the symptoms were mild. [multi code]

RANDOMISE ORDER OF STATEMENTS, anchor "none of these" and "don't know" to bottom

- New, continuous cough
- High temperature / fever
- Runny nose
- Diarrhoea
- Nausea / feeling sick
- Vomiting
- Sneezing
- Loss of appetite
- Loss of sense of smell (fully or partial)

Confidential: For Review Only

- Loss of taste
- None of these
- Don't know

Q30. Do you know if [CHILD] has had, or currently has, coronavirus? (Please select the option that BEST applies) [single code]

a) They have definitely had it or definitely have it now
b) They have probably had it or probably have it now
c) They have probably not had it and probably don't have it now
d) They have definitely not had it and definitely don't have it now

RANDOMISE ORDER OF Q31a AND Q31b SHOWN TO EACH RESPONDENT

Q31a. Please tell us how often each of these things happen to [CHILD]. There are no right or wrong answers.
SCALE

- *Never*
- *Sometimes*
- *Often*
- *Always*

STATEMENTS
- My child worries about things
- My child worries that something awful will happen to someone in the family
- My child worries that bad things will happen to him/her
- My child feels sad or empty
- Nothing is much fun for my child anymore
- My child has trouble sleeping
- My child has problems with his/her appetite
- My child has no energy for things

Q31b. Please tell us how often each of these things happen to [CHILD]. There are no right or wrong answers.
SCALE

- *Never*
- *Sometimes*
- *Often*
- *Always*

STATEMENTS

- My child worries that something bad will happen to him/her
- My child worries about what is going to happen
- My child thinks about death
- My child is tired a lot
- My child cannot think clearly
- My child feels worthless
- My child feels like he/she doesn't want to move
- My child feels restless

Q32. Thinking about next flu season (September 2020 – March 2021). During this time, you may be invited to vaccinate [CHILD] against flu. All primary school, but not secondary school children, are eligible for the child flu vaccine. For the following questions, please think about the next flu season.

For each of the following statements, please tell us to what extent, if at all, you agree or disagree:

I want [CHILD] to be vaccinated for flu next year (2020/21)

- Strongly agree
- Agree
- Neither agree nor disagree

Confidential: For Review Only

- Disagree
- Strongly disagree
- Not applicable, [CHILD] will be in secondary school in next school year (2020/21)

IF Q32 = a-e

Q33 I intend [CHILD] to be vaccinated for flu next year (2020/21)

- Strongly agree
- Agree
- Neither agree nor disagree
- Disagree
- Strongly disagree
- Not applicable, [CHILD] will be in secondary school in next school year (2020/21)

Thinking now about yourself…

Q34. Over the last 2 weeks, how often have you been bothered by the following problems?

SCALE

- Not at all
- Several days
- More than half the days
- Nearly every day

STATEMENTS

- Feeling nervous, anxious or on edge
- Not being able to stop or control worrying
- Little interest or pleasure in doing things
- Feeling down, depressed, or hopeless

And finally, the following questions ask for some more information about you and your household.

D2. Which gender do you identify yourself with? [single code]

- Male
- Female
- Prefer to self-describe
- Prefer not to say

D3. What is your employment status? [single code]

a. Full time paid job (31+ hours)
b. Part time paid job (<31 hours)
c. Doing paid work on a self-employed basis or within your own business
d. Employed, but currently furloughed
e. Student / On a government training programme (Nation Traineeship/Modern Apprenticeship)
f. Out of work (6 months or less)
g. Out of work (more than 6 months)
h. Looking after home / Homemaker
i. Retired
j. Disabled OR Long-term sick
k. Unpaid work for a business, community or voluntary organisation
l. Prefer not to say

If answer a/b/c to D3

D4. Are you currently working from home? [single code]

- Yes
- No

https://mc.manuscriptcentral.com/bmjpo

Confidential: For Review Only

D5. What is the highest level of educational qualification you have received? [single code]

a. PhD/Doctor
b. Master's
c. Bachelor's Degree or equivalent (Such as a NVQ level 5)
d. Higher education (Such as a HND or a NVQ level 4)
e. A level or equivalent (Such as Scottish Highers or NVQ level 3)
f. GCSE and below (Such as O level or an RSA Diploma)
g. Other qualifications (Such as NVQ level 1)
h. No qualifications
i. Prefer not to say

D6. Which of the following income brackets best represents your household income, before tax deductions for income tax, National Insurance etc? [single code]

a. Less than £5,000
b. £5,000-£9,999
c. £10,000-£14,999
d. £15,000-£19,999
e. £20,000-£24,999
f. £25,000-£29,999
g. £30,000-£34,999
h. £35,000-£39,999
i. £40,000-£44,999
j. £45,000-£49,999
k. £50,000-£59,999
l. £60,000-£69,999
m. £70,000-£84,999
n. £85,000-£99,999
o. More than £100,000
p. Prefer not to say

D7. What is your current marital or civil partnership status? [single code]

a. Single (i.e. never married and never registered as a same sex civil-partnership)
b. Co-habiting with partner (but never married or been in a civil partnership)
c. Civil partnership
d. Married
e. Separated, but still legally married / in a civil partnership
f. Divorced / Civil partnership legally dissolved
g. Widowed / Surviving partner from a same-sex civil partnership

D8. Which of the following categories would best describe your ethnicity? [single code]

a. English/Welsh/Scottish/Northern Irish/British
b. Irish
c. Gypsy or Irish Traveller
d. Other
e. White and Black Caribbean
f. White and Black African
g. White and Asian
h. Other
i. Indian
j. Pakistani
k. Bangladeshi
l. Chinese
m. Other
n. Caribbean
o. African
p. Other
q. Arab
r. Any other (please specify)
s. Prefer not to say

Confidential: For Review Only

D10. How many children in each age group live in your household? [number list]

- 0-3
- 4-10
- 11-15
- 16-18

Allow numerical input for each age group

D11. Do you live with anyone over the age of 70 years? [single code]

a. Yes
b. No

D12. Including yourself, how many people live in your household? [single code]

a. 2
b. 3
c. 4
d. 5
e. 6
f. 7
g. 8
h. 9
i. 10 or more

D9. Do any of the following have a medical condition than might make them particularly vulnerable to coronavirus?

SCALE

- Yes
- No
- Don't know

STATEMENTS

a. Yourself
b. [CHILD]
c. Anyone else you live with [show if D12 = b-i]

D13. [Record number] How many bedrooms does your home have?

D14. [Multi code] Which of the following do you have access to at your home:

a) A garden
b) A patio / terrace
c) A balcony
d) Another form of outdoor space
e) No outdoor space

Thank you for completing this survey. If you have any concerns about your child's mental health, please click here.

Confidential: For Review Only

Confidential: For Review Only

BMJ Paediatrics Open

Why did some parents not send their children back to school following school closures during the COVID-19 pandemic: a cross-sectional survey

Journal:	*BMJ Paediatrics Open*
Manuscript ID	bmjpo-2020-001014.R2
Article Type:	Original research
Date Submitted by the Author:	30-Apr-2021
Complete List of Authors:	Woodland, Lisa; Weston Education Centre<br>Smith, Louise; King's College London<br>Webster, Rebecca K; The University of Sheffield<br>Amlôt, Richard; Public Health England<br>Rubin, Antonia; Weald of Kent Grammar School<br>Wessely, Simon; Institute of Psychiatry Psychology and Neuroscience<br>Rubin, James
Keywords:	COVID-19, Psychology

SCHOLARONE™
Manuscripts

I, the Submitting Author has the right to grant and does grant on behalf of all authors of the Work (as defined in the below author licence), an exclusive licence and/or a non-exclusive licence for contributions from authors who are: i) UK Crown employees; ii) where BMJ has agreed a CC-BY licence shall apply, and/or iii) in accordance with the terms applicable for US Federal Government officers or employees acting as part of their official duties; on a worldwide, perpetual, irrevocable, royalty-free basis to BMJ Publishing Group Ltd ("BMJ") its licensees and where the relevant Journal is co-owned by BMJ to the co-owners of the Journal, to publish the Work in this journal and any other BMJ products and to exploit all rights, as set out in our licence.

The Submitting Author accepts and understands that any supply made under these terms is made by BMJ to the Submitting Author unless you are acting as an employee on behalf of your employer or a postgraduate student of an affiliated institution which is paying any applicable article publishing charge ("APC") for Open Access articles. Where the Submitting Author wishes to make the Work available on an Open Access basis (and intends to pay the relevant APC), the terms of reuse of such Open Access shall be governed by a Creative Commons licence – details of these licences and which Creative Commons licence will apply to this Work are set out in our licence referred to above.

Other than as permitted in any relevant BMJ Author's Self Archiving Policies, I confirm this Work has not been accepted for publication elsewhere, is not being considered for publication elsewhere and does not duplicate material already published. I confirm all authors consent to publication of this Work and authorise the granting of this licence.

Confidential: For Review Only

Why did some parents not send their children back to school following school closures during the COVID-19 pandemic: a cross-sectional survey

Lisa Woodland MSc, King's College London, Weston Education Centre, Cutcombe Road, London SE5 9RJ, UK

Louise E Smith PhD, King's College London, Weston Education Centre, Cutcombe Road, London SE5 9RJ, UK

Rebecca K Webster PhD, University of Sheffield, Cathedral Court, 1 Vicar Lane, Sheffield, S1 2LT, UK

Richard Amlôt PhD, Public Health England, Emergency Response Department, Porton Down, Salisbury, SP4 0JG, UK

Antonia Rubin MA (Cantab), Trustee at Weald of Kent Grammar School, Tudeley Lane, Tonbridge, Kent, TN9 2JP, UK.

Simon Wessely PhD, King's College London, Weston Education Centre, Cutcombe Road, London SE5 9RJ, UK

G James Rubin PhD, King's College London, Weston Education Centre, Cutcombe Road, London SE5 9RJ, UK

Correspondence to: Lisa Woodland, King's College London, Weston Education Centre, Cutcombe Road, London SE5 9RJ, UK, lisa.woodland@kcl.ac.uk

Word Count: 2,635

Confidential: For Review Only

ABSTRACT

Background

On 23rd March 2020, schools closed to most children in England in response to COVID-19 until September 2020. Schools were kept open to children of key workers and vulnerable children, on a voluntary basis. Starting 1st June 2020 children in reception (four to five years old), year one (five to six years old) and year six (10 to 11 years old) also became eligible to attend school.

Methods

1,373 parents or guardians of children eligible to attend school completed a cross-sectional survey between 8th and 11th June 2020. We investigated factors associated with whether children attended school or not.

Results

46% (n=370 / 803) of children in year-groups eligible to attend school and 13% (n=72 / 570) of children of key workers had attended school in the past seven days. The most common reasons for sending children to school were that the child's education would benefit, the child wanted to go to school, and the parent needed to work. A child was significantly more likely to attend if the parent believed the child had already had COVID-19, they had special educational needs, or a person in the household had COVID-19 symptoms.

Conclusions

Following any future school closure, helping parents to feel comfortable returning their child to school will require policy makers and school leaders to communicate about the adequacy of their policies to: a) ensure that the risk to children in school is minimised; b) ensure that the educational potential within schools is maximised; and c) ensure that the benefits of school for the psychological well-being of children are prioritised.

Key Words

Child; COVID-19; parents; school closures; England; cross-sectional studies

Confidential: For Review Only

ARTICLE ADDS

What is known about the subject

The COVID-19 pandemic resulted in the mass closure of schools for an extended period of time. A previous systematic review assessed parental attitudes towards smaller scale closures, finding that many parents (71%-97% across six studies) approved of closures, particularly where they were seen as an effective protective measure against a serious illness. The effect on a child's education and ability of the parent to work were reported as concerns.

What this study adds

During the partial reopening of schools in England in June 2020, most parents did not send their children to school. Parents who were not educated to degree level, not working, who lived in the North of England or who were from Black, Asian and ethnic minority backgrounds were least likely to send their children back. Perceived benefits of education, risk of disease and children's well-being were the main drivers in determining parental decision to send them to school or not.

Confidential: For Review Only

INTRODUCTION

On 23rd March 2020, a nationwide closure of schools occurred across England in response to the COVID-19 pandemic. Only vulnerable children (those with a health care plan or social worker) and children of key workers (critical to the COVID-19 response) were able to attend.[1,2] From 1st June 2020 children in reception (four to five years), year one (five to six years) and year six (10 to 11 years) also became eligible to attend.[3] Until September 2020 school attendance was voluntary to those children eligible to attend.[4]

The benefits of closing schools to reduce the transmission of COVID-19 and the negative consequences of doing so were difficult to balance.[5,6] Adding to the debate was emerging evidence of a low transmission rate of COVID-19 among children [7-10] and a recognition that outbreaks may occur nonetheless.[11,12]

Irrespective of this debate, it was clear that many parents felt far from comfortable with their children attending school in the first months of the pandemic, even where it was encouraged.[13] A worldwide systematic review of school closures suggests several factors that may be relevant to whether a child attended school during an infectious disease outbreak.[14] Nineteen papers were included in the review, samples representing between 67 and 4,171 school-aged children (five to 19 years). Perceived risk of infection,[15,16] concern about the impact of a closure on education,[15,17] and parental concerns about their child's mental health were key issues.[18] Understanding the key issues that determine whether a parent is willing to send their children back to school when it is partially open, and ensuring that school policies and communications address these concerns, should help inform re-opening schools, in this or any future pandemic.

In this study, we investigated factors associated with a parent's willingness to send their child to school when they partially re-opened, following closures due to the COVID-19 pandemic. We investigated these factors for children in reception, year one, or year six and for families where at least one parent was a key worker.

METHODS

Design

We commissioned a market research company, BMG Research to administer a cross-sectional survey one week after schools in England re-opened for children in reception, year one, and year six (8th to 11th June 2020).[19] We have previously reported data from this survey relating to parental perceptions of the presence of hygiene procedures within schools.[20]

Participants

Participants (n=2,447) were recruited from BMG Research's panel and to achieve a sample broadly representative of the population, BMG Research monitored region, child age, child gender, parent/guardian age, and parent/guardian gender. Participants were eligible for the study if they were aged eighteen years or over, lived

Confidential: For Review Only

in England, and were a parent or guardian to a school-aged child (4-18 years) who usually lived with them. 183 participants were screened out for non-eligibility, 226 participants dropped out after starting the survey and 28 completed but were removed for quality control such as completing the survey quickly or for 'straight-lining' (selecting the same option for every question) suggesting inattention to the questions. 2,010 participants remained. The sample fell within five percentage points of the national population by the child's gender, key stage, and type of school attended against the known distribution for school children in England.[21] The sample used in this paper were 803 parents of children in eligible school years and 570 parents from families in which at least one parent was a key worker (9 participants were removed from this group due to logical inconsistencies which suggested they had accidently completed the wrong section).

Participants were paid equivalent to £0.60.

Study Materials

The full survey is available in the supplementary materials.

Selection of a child to answer questions about

All participants answered questions referring to their child who had the most recent birthday. In cases where children shared a birthday, we asked the parent to select one child.

Our survey had two sections. Section one was only completed by parents who had a child in reception, year one, or year six or by parents who did not have a child in these year groups, but they or their spouse were a key worker. It contained questions about whether the child had attended school in the past week. Section two was completed by these parents and also by parents who did not have a child eligible to attend school. It contained general questions on views about risk of COVID-19, family living and school safety measures. In this paper we only report data from section one, relating to actual attendance in the past week.

Personal Characteristics

We asked participants to report their gender, age, region, household income, employment status, marital status, ethnicity, and level of education. We also asked whether anyone within the household was aged over 70 years or had a health condition that made them vulnerable to COVID-19.

We asked participants to report the child's gender, age, school year, school type (fee-paying or state-funded) and whether the child had special educational needs (SEN).

School Attendance

Participants were asked how many times the child had attended school in the past seven days. Depending on the response, parents were presented with randomised statements: 10 for why they were sending the child to school; 12 for why they were only sending the child to school part-time; or 16 why their child was not attending

Confidential: For Review Only

school. We asked participants to “tick any [statement] that applies”. Participants also had the option to write-in text for “other reason”.

COVID-19 Symptoms

We asked participants to report if the selected child had experienced any symptoms “in the past seven days” from a list of ten symptoms. We also asked if they or a household member (other than the child) had experienced symptoms “in the past 14 days” from the same symptom list. We asked participants whether they thought their child had had COVID-19.

Well-Being

We asked participants to report the child’s well-being using two subscales from the Revised Child Anxiety Disorder Scale (RCADS):[22] the generalised anxiety disorder (GAD) sub-scale and the major depressive disorder (MDD) sub-scale.

ETHICS

The research was approved by the Psychiatry, Nursing and Midwifery Research Ethics Subcommittee at King’s College London (LRS—19/20-18787).

PATIENT AND PUBLIC INVOLVEMENT.

A school trustee contributed to the development of the survey materials and co-authored this paper.

ANALYSIS

Recoding of variables

School attendance was defined as a child who attended school for at least one day, in the past seven days.

We created two binary variables to indicate whether the child, and someone in the household (other than the child) had a health condition that might make them particularly vulnerable to COVID-19.

We created two binary variables to indicate recent presence of COVID-19 symptoms in the child, and someone else in the household. We defined presence of COVID-19 symptoms as experiencing a “new, continuous cough”, “high temperature / fever”, “loss of sense of smell (fully or partial)”, or “loss of taste”. We coded a binary variable for the parent’s perception of whether the child had had COVID-19 by grouping together “they have definitely had it or definitely have it now” and “they have probably had it or probably have it now”.

We created a binary variable to indicate low well-being in the child. We assigned a value against each answer from 0 (“never”) to 3 (“always”) on the GAD and MDD RCADS sub-scales and created a total score for each sub-scale. We then turned each total score into a t-score, a method used to normalise RCADS scores within the population, by child’s age and gender.[22,23] We used the same process for

Confidential: For Review Only

reception to year three as for year four. We used a t-score cut off of 65 or above on either GAD or MDD sub-scales to indicate low well-being.

For all variables, we coded the responses “don’t know”, “not applicable”, “prefer not to say” and “prefer to self-describe” as missing data.

Analysis

We ran a series of binary logistic regressions using SPSS v26.0,[24] investigating univariable associations between each of our predictor variables and sending the child to school. We ran a second set of binary logistic regressions controlling for personal characteristics shown in the results.

We analysed frequencies for the statements responding to sending the child to school for a full week (five days), part-time (less than five days), and for not sending the child to school.

For ease of interpretation, we used unweighted data in our analysis.

We applied a Bonferroni correction to our results ($p \leq .001$) due to running many analyses (children in school years n=29 and children of key workers n=31). Results meeting this criterion are marked by a double asterisk (**) in the tables.

Sample size calculation

Post-hoc calculations were run on the two sub-samples which had a margin of error of plus or minus 5% at the 95% confidence level for each prevalence estimate.

RESULTS

School attendance

Nearly half (46% CI, 43% to 50%, n=370/803) of children in eligible school years had attended school and about half had not (54% 95% CI, 50% to 57%, n=432/803). One participant was unsure if the child had attended school. The most common reasons for not sending a child to school were: thinking it was too risky for the child to attend school (n=223, 52%), the school not being open (n=140, 32%) and having another child who could not go to school (n=67, 16%).The most common reasons for sending a child to school were: the child’s education would benefit (n=208, 56%), the child wants to go to school (n=200, 54%), and the child will benefit from seeing their friends (n=187, 51%). Of participants whose child did not attend school for a full week (n=204, 25%) the most common reasons for partial attendance were: the school only offers them to be in part-time (n=80, 39%), it is less risky for them to be in part-time (n=40, 20%), and I am only sending them in on days where the lessons are important (n=28, 14%).

Only 13% (95% CI, 10% to 15%, n=72/570) of children of key workers had attended school, most children had not (87% 95% CI, 85% to 90%, n = 497/570). One participant was unsure if the child had attended school. The most common reasons for children not attending school were: the school was not open (n=259, 52%), the school had asked the child not to attend (n=117, 24%), and thinking that it was too

Confidential: For Review Only

risky for the child to attend school at the moment (n=109, 22%). The most common reasons for attending school were: the parent needing to work (n=40, 56%), the child wanting to go to school (n=35, 49%), and thinking that the child's education would benefit from being at school (n=30, 42%). Of participants with children who attended school part-time the most common reasons were: the school only offered them to be in part-time (n=16, 41%), parent only sent them in on days where the lessons are important (n=8, 21%), and believing it was less risky for them to be in part-time (n=7, 18%).

Associations

Participant characteristics for children in eligible school years and for children of key workers are shown in table 1. Parents of children in eligible school years were less likely to send the child to school if they were educated to A-level or below, not working, of Black, Asian and minority ethnicity (BAME), or living in the North East, North West, and Yorkshire and the Humber compared to London. Whereas children of key workers were more likely to attend school for participants aged 45 and under and who were working. Child attendance was more likely for children in eligible school years when in a fee-paying school and if they had a health condition that made them vulnerable to COVID-19. For both groups, attendance was more likely for children who had special educational needs, reported low well-being, thought their child had had COVID-19 and when a person over 70 was living in the household. There was also a significant association in both groups between a child being more likely to attend school and the child having experienced COVID-19 symptoms in the past 7 days, or another person in the household having experienced COVID-19 symptoms in the past 14 days.

Confidential: For Review Only

Table 1. Participant characteristics for children in eligible school years (n=803) and children of key workers (n=570), by school attendance.

		Children in Eligible school years				Children of key workers			
	Level	Did not attend school, n (%)	Attended School, n (%)	Odds ratio (95% CI)	Adjusted odds ratio (95% CI) †	Did not attend school, n (%)	Attended School, n (%)	Odds ratio (95% CI)	Adjusted odds ratio (95% CI) †
Gender	Male	201 (52)	187 (48)	1·22 (0·92 to 1·61)	1·04 (0·75 to 1·43)	227 (85)	39 (15)	1·53 (0·92 to 2·55)	1·61 (0·91 to 2·85)
	Female	230 (57)	177 (43)	Reference	Reference	268 (90)	30 (10)	Reference	Reference
Age	18-35 years	129 (49)	136 (51)	1·37 (0·92 to 2·03)	1·23 (0·79 to 1·92)	54 (77)	16 (23)	**3·16* (1·56 to 6·37)**	**2·95* (1·23 to 7·08)**
	36-45 years	212 (56)	164 (44)	1·01 (0·70 to 1·46)	0·98 (0·65 to 1·48)	198 (86)	33 (14)	**1·76* (1·01 to 3·12)**	**2·14* (1·12 to 4·09)**
	46 years ≥	91 (56·5)	70 (43·5)	Reference	Reference	245 (91)	23 (9)	Reference	Reference
Region	East Midlands	33 (60)	22 (40)	**0·50* (0·27 to 0·93)**	0·59 (0·31 to 1·14)	45 (85)	8 (15)	0·85 (0·32 to 2·22)	1·23 (0·40 to 3·75)
	East of England	38 (48)	41 (52)	0·81 (0·48 to 1·38)	0·92 (0·52 to 1·64)	54 (82)	12 (18)	1·06 (0·45 to 2·52)	1·38 (0·50 to 3·81)
	North East	33 (67)	16 (33)	**0·36* (0·19 to 0·71)**	**0·38* (0·81 to 0·78)**	32 (89)	4 (11)	0·60 (0·18 to 1·98)	0·93 (0·25 to 3·46)
	North West	68 (65)	36 (35)	**0·40** (0·24 to 0·66)**	**0·39* (0·23 to 0·68)**	80 (93)	6 (7)	**0·36* (0·13 to 0·99)**	0·61 (0·19 to 1·89)
	South East	70 (51)	66 (49)	0·71 (0·45 to 1·11)	0·92 (0·56 to 1·52)	82 (89)	10 (11)	0·58 (0·24 to 1·94)	0·81 (0·29 to 2·29)
	South West	24 (46)	28 (54)	0·88 (0·47 to 1·63)	1·16 (0·58 to 2·31)	42 (87·5)	6 (12·5)	0·68 (0·42 to 1·94)	1·18 (0·37 to 3·82)
	West Midlands	39 (56)	30 (44)	0·58 (0·33 to 1·01)	0·63 (0·34 to 1·18)	48 (89)	6 (11)	0·60 (0·21 to 1·68)	0·84 (0·27 to 2·60)
	Yorkshire & the Humber	52 (62)	31 (37)	**0·45* (0·26 to 0·76)**	**0·52* (0·29 to 0·93)**	52 (88)	7 (12)	0·64 (0·24 to 1·73)	0·80 (0·27 to 2·41)
	London	75 (43)	100 (57)	Reference	Reference	62 (83)	13 (17)	Reference	Reference
Household income	≤ £34,999	164 (54)	142 (46	1·00 (0·75 to 1·33)	1·34 (0·95 to 1·90)	185 (89)	23 (11)	0·75 (0·44 to 1·28)	0·96 (0·51 to 1·81)
	£35,000 ≥	254 (54)	220 (46)	Reference	Reference	285 (86)	47 (14)	Reference	Reference
Employment status[1]	Working	362 (52)	337 (48)	**1·95* (1·25 to 3·05)**	**1·94* (1·16 to 3·24)**	435 (86)	70 (14)	**9·33* (1·27 to 68·47)**	**8·64* (1·13 to 65·96)**
	Not working	67 (68)	32 (32)	Reference	Reference	58 (98)	1 (2)	Reference	Reference
Education level	≤ A-level	179 (63)	105 (37)	**0·56** (0·41 to 0·75)**	**0·59* (0·42 to 0·84)**	227 (88)	31 (12)	0·91 (0·55 to 1·51)	1·27 (0·71 to 2·26)
	Degree ≥	248 (49)	261 (51)	Reference	Reference	267 (87)	40 (13)	Reference	Reference
Marital status	Living alone	49 (48)	54 (52)	1·34 (0·88 to 2·02)	1·30 (0·82 to 2·06)	75 (88)	10 (12)	0·91 (0·45 to 1·85)	0·92 (0·41 to 2·03)
	Married/cohabiting	383 (55)	316 (45)	Reference	Reference	422 (87)	62 (13)	Reference	Reference
Ethnicity	White	355 (53)	316 (47)	1·23 (0·84 to 1·12)	**1·66* (1·07 to 2·58)**	450 (87)	65 (13)	1·04 (0·42 to 2·53)	1·47 (0·53 to 4·13)
	BAME	72 (58)	52 (42)	Reference	Reference	43 (78)	12 (22)	Reference	Reference
Child gender	Boy	230 (51·5)	217 (48·5)	1·25 (0·94 to 1·65)	1·15 (0·85 to 1·56)	244 (87)	36 (13)	1·04 (0·63 to 1·70)	0·96 (0·56 to 1·66)
	Girl	202 (57)	153 (43)	Reference	Reference	253 (87·5)	36 (12·5)	Reference	Reference
School year	Early Years	77 (59)	53 (41)	0·74 (0·50 to 1·10)	0·79 (0·51 to 1·23)	***	***	***	***
	Key Stage 1	157 (54)	132 (46)	0·90 (0·66 to 1·22)	0·88 (0·63 to 1·24)	30 (86)	5 (14)	2·10 (0·57 to 7·81)	1·52 (0·37 to 6·30)
	Key Stage 2	198 (52)	185 (48)	Reference	Reference	120 (86)	20 (15)	2·10 (0·75 to 5·86)	1·45 (0·47 to 4·44)
	Key Stage 3	***	***	***	***	152 (87)	23 (13)	1·91 (0·69 to 5·24)	1·58 (0·55 to 4·55)
	Key Stage 4	***	***	***	***	132 (87)	19 (13)	1·81 (0·65 to 5·08)	1·44 (0·49 to 4·24)
	Years 12 & 13	***	***	***	***	63 (93)	5 (7)	Reference	Reference
School type	Fee-paying	35 (33)	71 (67)	**2·68** (1·74 to 4·13)**	**2·50** (1·56 to 4·00)**	30 (77)	9 (23)	**2·20* (1·00 to 4·85)**	2·04 (0·82 to 5·07)
	State-funded	395 (57)	299 (43)	Reference	Reference	462 (88)	63 (12)	Reference	Reference
Child has SEN	Yes	22 (31)	48 (69)	**2·78** (1·64 to 4·70)**	**2·30* (1·27 to 4·17)**	28 (61)	18 (39)	**5·74** (2·97 to 11·08)**	**7·92** (3·59 to 17·46)**
	No	406 (56)	319 (44)	Reference	Reference	464 (90)	52 (10)	Reference	Reference
Low child well-being	Yes	92 (42)	124 (57)	**1·86** (1·36 to 2·55)**	**1·47* (1·04 to 2·07)**	127 (82)	28 (18)	**1·85* (1·11 to 3·10)**	**1·78* (1·00 to 3·21)**

Confidential: For Review Only

9

	No	340 (58)	246 (42)	Reference	Reference	370 (89)	44 (11)	Reference	Reference
Child vulnerable COVID-19	Yes	28 (39)	44 (61)	**2·00* (1·21 to 3·28)**	**1·76* (1·00 to 3·10)**	33 (82·5)	7 (17·5)	1·49 (0·63 to 3·50)	0·93 (0·33 to 2·63)
	No	401 (56)	316 (44)	Reference	Reference	455 (87·5)	65 (12·5)	Reference	Reference
Household vulnerable COVID-19	Yes	115 (60)	78 (40)	0·78 (0·56 to 1·08)	0·74 (0·51 to 1·06)	134 (88)	18 (12)	0·90 (0·50 to 1·60)	0·56 (0·44 to 1·67)
	No	295 (53)	258 (47)	Reference	Reference	314 (87)	47 (13)	Reference	Reference
Child COVID-19 symptoms	Yes	15 (24)	47 (76)	**5·05** (2·22 to 7·36)**	**3·02* (1·49 to 6·15)**	11 (58)	8 (42)	**5·52** (2·14 to 14·24)**	**5·25* (1·58 to 17·41)**
	No	417 (56)	323 (44)	Reference	Reference	486 (88)	64 (12)	Reference	Reference
Household COVID-19 symptoms	Yes	9 (17)	43 (83)	**6·18** (2·97 to 12·86)**	**6·04** (2·62 to 13·91)**	13 (65)	7 (35)	**4·00* (1·54 to 10·42)**	**4·11* (1·26 to 13·44)**
	No	423 (56)	327 (43)	Reference	Reference	484 (88)	65 (12)	Reference	Reference
Child has had or currently has COVID-19	Yes	36 (24)	112 (76)	**4·78** (3·18 to 7·17)**	**4·03** (2·57 to 6·30)**	40 (67)	20 (33)	**4·40** (2·40 to 8·08)**	**5·20** (2·48 to 10·93)**
	No	396 (61)	258 (37)	Reference	Reference	457 (90)	52 (10)	Reference	Reference
Someone over 70 years	Yes	11 (26)	32 (74)	**3·62** (1·80 to 7·30)**	**3·19* (1·45 to 7·05)**	8 (57)	6 (43)	**5·56* (1·87 to 16·52)**	**3·49* (0·98 to 12·46)**
	No	421 (55·5)	338 (44·5)	Reference	Reference	489 (88)	66 (12)	Reference	Reference

* *p*≤ *·05* and formatted bold

** *p*≤ *·001* and formatted bold

*** data not applicable for the sample

† Adjusting for participant gender, age, region, household income, employment status, education level, marital status, participant ethnicity, and the child's gender, school year and school-type.

[1] Working includes students and volunteers

Decimal places have been used for rounding errors

Confidential: For Review Only

DISCUSSION

Most children eligible to attend school did not attend one week after schools in England began to re-open to more children.[25] Worryingly, we observed patterns that seemed likely to entrench existing educational inequalities. Children from households where parents have lower education achievements, BAME households and households in the North of England, and state-funded being least likely to attend school.

Our results suggest that several broad areas determined attendance at school. First, risk perceptions were crucial. The response “it is too risky” featured highly in parental reasons for not sending children to school. Secondly, children were also more likely to attend school when parents thought their child had had COVID-19 which could indicate the belief their child is immune to further infection.[26] Unexpectedly, children were more likely to attend school when they had a health condition that made them vulnerable to COVID-19 and when a person over 70 years was living in the household. We speculate that this may be because these circumstances affect the parent’s ability to look after the child at home.

Poorer perceived child well-being was associated with them being more likely to attend school. This finding may reflect parental desire to improve their child’s well-being which may have been impacted by the school closure.[27] This was also apparent in reasons parents gave for sending their child to school. As expected,[14] concerns about education also featured highly as a reason for attendance, while perceptions that schools could not provide good quality education or that some lessons were not as important were cited as reasons for absence or partial attendance.

Despite most children of key workers not attending school, needing to work was the most reported reason for school attendance and the school not opening or asking the child not to attend were the most commonly reported reasons for the child not being in school. This suggests that work commitments were the main driver for this group.

One notable finding was that children who had symptoms of COVID-19 in the past seven days, or whose household members had these symptoms in the past 14 days were significantly more likely to have gone to school. We do not know if the child attended school whilst having symptoms or when symptoms were present in the household (against self-isolation guidance)[28]. However, given that school closure is specifically designed to reduce the transmission of respiratory infections in general, it is also possible that this reflects the re-emergence of transmission of upper respiratory tract infections, or COVID-19 specifically, within the school environment.[29] It may also reflect increased anxiety or awareness among parents around COVID-19 symptoms, resulting in higher symptom detection and reporting.

LIMITATIONS

Several limitations should be borne in mind for this study. First the cross-sectional nature of this study limits our ability to draw causal findings. Second, the RCADS subscale was used to indicate low well-being but is currently not validated for children under eight years.[30] Third, online polls can be unrepresentative, and lead to response, and self-reporting bias.[31] However, in line with the reasoning relating to the use of non-probability samples in social sciences, [32] we assume that the associations within our data do generalise to the wider population. Fourth, we ran many analyses raising the possibility of Type 1 errors. While we have provided Bonferroni corrections in the tables for readers who wish to correct this, this correction in turn may be overly conservative.

CONCLUSION

Our findings support previous research by suggesting that during an infectious disease outbreak, parents' decision to send their child to school was impacted by the risk of disease (COVID-19), child's education and well-being. Furthermore, without reassuring parents in these three areas and encouraging them to send their children to school health inequalities are likely to be increased.

Contributors

All authors contributed to the conceptualisation of the study and approved the final draft. LW designed the survey, analysed the data and drafted the manuscript. LES designed the survey, analysed the data and edited the manuscript. RKW edited the manuscript. RA designed the survey and edited the manuscript. AR designed the survey and edited the manuscript. SW edited the manuscript. GJR designed the survey, analysed the data and edited the manuscript.

Declarations of interests

We declare no competing interests.

Acknowledgments

This study was funded by the Economic and Social Research Council and the National Institute for Health Research Health Protection Research Unit (NIHR HPRU) [grant number NIHR200890] in Emergency Preparedness and Response, a partnership between Public Health England, King's College London and the University of East Anglia. The views expressed are those of the authors and not necessarily those of the NIHR, Public Health England or the Department of Health and Social Care.

Role of the funding source

Confidential: For Review Only

The funders had no role in study design, data collection, data analysis, data interpretation, or writing of the manuscript. The corresponding author had full access to all the data and had final responsibility for the decision to submit for publication.

Confidential: For Review Only

REFERENCES

1 Prime Minister B Johnson. PM statement on coronavirus: 18 March 2020. Available at: https://www.gov.uk/government/speeches/pm-statement-on-coronavirus-18-march-2020?utm_source=407969fc-4de1-41c8-9571-f6b6ad35efae&utm_medium=email&utm_campaign=govuk-notifications&utm_content=immediate Accessed 11 June 2020.
2 Government. H. Children of critical workers and vulnerable children who can access schools or educational settings. Available at: https://www.gov.uk/government/publications/coronavirus-covid-19-maintaining-educational-provision/guidance-for-schools-colleges-and-local-authorities-on-maintaining-educational-provision Accessed 16 March 2021.
3 Prime Minister B Johnson. PM confirms schools, colleges and nurseries on track to begin phased reopening. Available at: https://www.gov.uk/government/news/pm-confirms-schools-colleges-and-nurseries-on-track-to-begin-phased-reopening Accessed 11 June 2020.
4 HM Government. School Attendance. Available at: https://www.gov.uk/government/publications/school-attendance Accessed 8 July 2020.
5 Viner RM, Russell SJ, Croker H, et al. School closure and management practices during coronavirus outbreaks including COVID-19: a rapid systematic review. *The Lancet Child & Adolescent Health*. 2020;4(5):397-404 DOI: 10.1016/s2352-4642(20)30095-x
6 Keogh-Brown MR, Jensen HT, Edmunds WJ, Smith RD. The impact of Covid-19, associated behaviours and policies on the UK economy: A computable general equilibrium model. *SSM - Population Health*. 2020;[in press]:100651 DOI: 10.1016/j.ssmph.2020.100651
7 Boast A, Munro A, Goldstein H. An evidence summary of Paediatric COVID-19 literature. Available at: https://dontforgetthebubbles.com/evidence-summary-paediatric-covid-19-literature/ Accessed 8 July 2020.
8 Zhu Y, Bloxham C, K H, et al. Children are unlikley to have been the primary source of household SARS-CoV-2 infections. 2020; DOI: 10.1101/2020.03.26.20044826v1
9 World Health Organization. Report of the WHO-China joint mission on coronavirus disease 2019 (COVID-19). Available at: https://www.who.int/publications/i/item/report-of-the-who-china-joint-mission-on-coronavirus-disease-2019-(covid-19) Accessed 8 July 2020.
10 Heavey L, Casey G, Kelly C, et al. No evidence of secondary transmission of COVID-19 from children attending school in Ireland, 2020. *Eurosurveillance*. 2020;25(21) DOI: 10.2807/1560-7917.es.2020.25.21.2000903
11 Otte Im Kampe E, Lehfeld A-S, Buda S, et al. Surveillance of COVID-19 school outbreaks, Germany, March to August 2020. *Eurosurveillance*. 2020;25(38) DOI: 10.2807/1560-7917.es.2020.25.38.2001645
12 England. PH. SARS-CoV-2 infection and transmission in educational settings. Available at: https://www.gov.uk/government/publications/sars-cov-2-infection-and-transmission-in-educational-settings Accessed 22 October 2020.
13 Royal College of Paediatrics and Child Health. COVID_19 - talking to children and families about returning to school: guiding principles. Available at: https://www.rcpch.ac.uk/resources/covid-19-talking-children-families-about-returning-school-guiding-principles Accessed 9 July 2020.

Confidential: For Review Only

14 Brooks SK, Smith LE, Webster RK, et al. The impact of unplanned school closure on children's social contact: rapid evidence review. *Euro Surveill*. 2020;25(13) DOI: 10.2807/1560-7917.ES.2020.25.13.2000188
15 Basurto-Dávila R, Garza R, Meltzer MI, et al. Household economic impact and attitudes toward school closures in two cities in Argentina during the 2009 influenza A (H1N1) pandemic. *Influenza and Other Respiratory Viruses*. 2013;7(6):1308-15 DOI: 10.1111/irv.12054
16 Effler PV, Carcione D, Giele C, et al. Household responses to pandemic (H1N1) 2009-related school closures, Perth, Western Australia. *Emerg Infect Dis*. 2010;16(2):205-11 DOI: 10.3201/eid1602.091372
17 van Gemert C, McBryde ES, Bergeri I, et al. Description of social contacts among student cases of pandemic influenza during the containment phase, Melbourne, Australia, 2009. *Western Pac Surveill Response J*. 2018;9(5 Suppl 1):27-34 DOI: 10.5365/wpsar.2018.9.5.003
18 Lee J. Mental health effects of school closures during COVID-19. *The Lancet Child & Adolescent Health*. 2020;4(6):421 DOI: 10.1016/s2352-4642(20)30109-7
19 Research. B. BMG About Us. Available at: https://www.bmgresearch.co.uk/bmg/about-us/ Accessed 16 March 2021.
20 Smith LE, Woodland L, Amlôt R, et al. A cross-sectional survey of parental perceptions of COVID-19 related hygiene measures within schools and adherence to social distancing in journeys to and from school. *BMJ Paediatrics Open*. 2020;4(1) DOI: 10.1136/bmjpo-2020-000825
21 HM Government. Schools, pupils and their characteristics: Academic Year 2019/20. Available at: https://explore-education-statistics.service.gov.uk/find-statistics/school-pupils-and-their-characteristics Accessed 25 June 2020.
22 Chorpita BF, Yim L, Moffitt C, et al. Assessment of symptoms of DSM-IV anxiety and depression in children: a revised child anxiety and depression scale. *Behaviour Research and Therapy*. 2000;38(8):835-55 DOI: 10.1016/s0005-7967(99)00130-8
23 Chorpita BF. Child FIRST - Focus on innovation and redesign in systems and treatment. Available at: https://www.childfirst.ucla.edu/resources/ Accessed 6 July 2020.
24 IBM Corp.2019. IBM SPSS Statistics for Windows, 26.0 Armonk, NY.
25 HM Government. attendance in education and early years settings during the coronavirus (COVID-19) outbreak. Available at: https://www.gov.uk/government/publications/coronavirus-covid-19-attendance-in-education-and-early-years-settings?utm_source=efcd291f-83ee-4d73-860a-7e823b3c0017&utm_medium=email&utm_campaign=govuk-notifications&utm_content=immediate Accessed 9 June 2020.
26 Smith LE, Mottershaw AL, Egan M, et al. The impact of believing you have had COVID-19 on self-reported behaviour: Cross-sectional survey. *PLOS ONE*. 2020;15(11):e0240399 DOI: 10.1371/journal.pone.0240399
27 Idoiaga M, Berasategi S, Dosil S, Eiguren M. Struggling to breathe: a qualitative study of children's wellbeing during lockdown in Spain. *Psychology & Health*. 2020:1-16 DOI: 10.1080/08870446.2020.1804570
28 HM Government. Protective measures for out-of-school settings during the coronavirus (COVID-19) outbreak. Available at: https://www.gov.uk/government/publications/protective-measures-for-holiday-or-after-school-clubs-and-other-out-of-school-settings-for-children-during-the-coronavirus-covid-19-outbreak/protective-measures-for-out-of-school-settings-during-the-coronavirus-covid-19-outbreak Accessed 8 July 2020.

Confidential: For Review Only

29 Panovska-Griffiths J, Kerr CC, Stuart RM, et al. Determining the optimal strategy for reopening schools, the impact of test and trace interventions, and the risk of occurrence of a second COVID-19 epidemic wave in the UK: a modelling study. *The Lancet Child & Adolescent Health*. 2020; DOI: 10.1016/s2352-4642(20)30250-9
30 Ebesutani C, Chorpita BF, Higa-Mcmillan CK, et al. A Psychometric Analysis of the Revised Child Anxiety and Depression Scales—Parent Version in a School Sample. *Journal of Abnormal Child Psychology*. 2011;39(2):173-85 DOI: 10.1007/s10802-010-9460-8
31 Epstein WM. Response bias in opinion polls and American social welfare. *The Social Science Journal*. 2006;43(1):99-110 DOI: 10.1016/j.soscij.2005.12.010
32 Kohler U. Possible Uses of Nonprobability Sampling for the Social Sciences. *Survey Methods: Insights from the Field*. Available at: https://surveyinsights.org/?p=10981 Accessed 29 July 2020.

Confidential: For Review Only

SUPPLEMENTARY MATERIAL

Table 1: Table of frequencies to show reasons children did not attend school.

Reason	Children in School Years (n=432, %)	Children of Key Workers (n=497, %)
I think it is too risky for my child to attend school at the moment	223 (52%)	109 (22%)
The school is not open	140 (32%)	259 (52%)
I have another child who can't go to school	67 (15·5%)	24 (5%)
Being at school will be stressful for my child	59 (14%)	23 (5%)
I think they will get a better education at home than at school at the moment	57 (13%)	25 (5%)
The school has asked my child not to attend	51 (12%)	117 (23·5%)
Someone in my household is clinically vulnerable to coronavirus	45 (10%)	24 (5%)
My child doesn't want to go	44 (10%)	29 (6%)
There's no point, schools will shut again soon anyway	37 (9%)	13 (3%)
My child's friends will not be at school	28 (6·5%)	19 (4%)
Other reason	28 (6·5%)	65 (13%)
I think friends, family or other parents will judge me if I send my child to school	12 (3%)	4 (1%)
I can't arrange transport to get them to school	6 (1%)	10 (2%)
Someone else at the school developed coronavirus symptoms (cough or fever, or change in sense of taste or smell)	6 (1%)	4 (1%)
My child developed symptoms of a different illness	3 (1%)	1 (0%)
They have developed coronavirus symptoms (cough or fever, or change in sense of taste or smell)	2 (0·5%)	2 (0%)
Someone else in the family developed coronavirus symptoms (cough or fever, or change in sense of taste or smell)	0 (0%)	3 (1%)

Table 2: Table of frequencies to show reasons children attended school.

Reason	Children in School Years (n=370, %)	Children of Key Workers (n=72, %)
My child's education will benefit from being at school	208 (56%)	30 (42%)
My child wants to go to school	200 (54%)	35 (49%)
My child will benefit from seeing their friends	187 (50·5%)	27 (37·5%)
I need to work	120 (32%)	40 (56%)
I don't believe my child will be at risk at school	96 (26%)	17 (24%)
Having my child at home is a strain	57 (15%)	10 (14%)
It is compulsory for my child to attend	50 (13.5%)	9 (12.5%)
It reduces costs at home	48 (13%)	7 (10%)
I think friends, family or other parents will judge me if I don't send my child to school	43 (12%)	11 (15%)
I have non-work commitments I need to meet	21 (6%)	4 (6%)
Other reason	9 (2%)	3 (4%)

Table 3: Table of frequencies to show reasons children attended school part-time (less than five days).

Reason	Children in School Years (n=204, %)	Children of Key Workers (n=39, %)
The school only offers for them to be in part-time	80 (39%)	16 (41%)
It is less risky for them to be in part-time	40 (20%)	7 (18%)

Confidential: For Review Only

I am only sending them in on days where the lessons are important	28 (14%)	8 (20·5%)
Other reason	27 (13%)	3 (8%)
I am using it as childcare and I only need them in part time	24 (12%)	6 (15%)
I am only sending them in on days when I can arrange transport	23 (11%)	4 (10%)
Someone else at the school developed coronavirus symptoms (cough or fever, or change in sense of taste or smell)	14 (7%)	1 (3%)
I started sending them in, but have now changed my mind	13 (6%)	2 (5%)
They have developed coronavirus symptoms (cough or fever, or change in sense of taste or smell)	13 (6%)	1 (3%)
Someone else in the family developed coronavirus symptoms (cough or fever, or change in sense of taste or smell)	12 (6%)	0 (0%)
I am only sending them in on days when their friends are in	11 (5%)	6 (15%)
My child developed symptoms of a different illness	9 (4%)	0 (0%)
They only live with me part time	6 (3%)	1 (3%)

Confidential: For Review Only

Full Survey Material

D1. Please type your age on your last birthday: [number]

Screenout if D1 = <18

Q1. Which country do you live in? [single code]

a. England
b. Scotland
c. Wales
d. Northern Ireland
e. Outside of the UK

Screenout if Q1 = b-e

Q2. Which region do you live in? [single code]

a. East Midlands
b. East of England
c. London
d. North East
e. North West
f. South East
g. South West
h. West Midlands
i. Yorkshire and the Humber

Q3. Are you the parent or guardian of a child who usually attends school (that is, before the coronavirus outbreak occurred) and who lives with you? [single code]

a. Yes
b. No

Screenout if Q3 = b

Q4. Which of the following age groups do your children who live with you fall into? Please tick any that apply. [multi code]

a. 0-3
b. 4-11
c. 12-18
d. None of the above [exclusive]

Screenout if b OR c not selected

Q5. The Government has categorised some people as "critical workers" or "key workers." For example, this includes people working in health and social care, education, key public services, transport, food distribution and other jobs. As far as you know, are you, or a spouse/partner, a "critical" or "key" worker? Please tick any that apply. [multi code]

a. Yes, me
b. Yes, spouse/partner
c. No [exclusive]
d. Don't know [exclusive]

IF Q4 = b

Q6. You stated that you have at least one child aged 4-11 who lives with you. Are any of your children who live with you in the following school years? Please tick any that apply. [multi code]

a. Reception
b. Year 1
c. Year 6

Confidential: For Review Only

d. I have no children in the school years listed above [exclusive]

IF 2 or more options are selected at Q6

Q7. Thinking only about your children in Reception, Year 1 or Year 6, who live with you, which child had the most recent birthday? If the most recent birthday was shared by more than one child (e.g. twins, or children born on the same day in different years), please answer the following questions thinking about one of these children only. [single code]

a. My child in Reception
b. My child in Year 1
c. My child in Year 6

ONLY SHOW OPTIONS SELECTED AT Q6

IF Q6 = a-c

Q8. Can you please tell us how old that child is? [single code]

a. 4
b. 5
c. 6
d. 7
e. 8
f. 9
g. 10
h. 11

IF Q6 = a-c

Q9. Can you please tell us if that child is a: [single code]

a. Boy
b. Girl

IF Q6 = a-c

Q10. Can you please tell us the type of school that child usually attends? [single code]

a. Fee-paying
b. State-funded school
c. Don't know

IF Q6 = a-c

Q11A. Thinking about that child, have they attended school at all in the past 7 days? [single code]

a. Yes, on 5 days
b. Yes, on 1 to 4 days
c. No
d. Don't know

IF Q11A = a/b

Q12A. You said that your child has attended school on at least one day in the past week. For which reasons did you send your child to school? Please tick any that apply [multi code]:

RANDOMISE ORDER OF STATEMENTS, anchor other to bottom

a) It is compulsory for my child to attend
b) I need to work
c) I have non-work commitments I need to meet
d) My child's education will benefit from being at school
e) My child will benefit from seeing their friends
f) Having my child at home is a strain
g) I don't believe my child will be at risk at school

Confidential: For Review Only

h) My child wants to go to school
i) I think friends, family or other parents will judge me if I don't send my child to school
j) It reduces costs at home
k) Other reason [write in]

IF Q11A = b

Q13A. You said that your child has not attended school every day in the past week. For which reasons are they attending only part-time? Please tick any that apply [multi code]:

RANDOMISE ORDER OF STATEMENTS, anchor other to bottom

a) I am using it as childcare and I only need them in part time
b) It is less risky for them to be in part-time
c) The school only offers for them to be in part-time
d) I am only sending them in on days where the lessons are important
e) I am only sending them in on days when their friends are in
f) I am only sending them in on days when I can arrange transport
g) They only live with me part time
h) I started sending them in, but have now changed my mind
i) They have developed coronavirus symptoms (cough or fever, or change in sense of taste or smell)
j) My child developed symptoms of a different illness
k) Someone else in the family developed coronavirus symptoms (cough or fever, or change in sense of taste or smell)
l) Someone else at the school developed coronavirus symptoms (cough or fever, or change in sense of taste or smell)
m) Other reason [write in]

IF Q11A = c

Q14A. You said that your child has not attended school in the past week. For which reasons did you not send your child to school? Please tick any that apply [multi code]:

RANDOMISE ORDER OF STATEMENTS, anchor other to bottom

a) Someone in my household is clinically vulnerable to coronavirus
b) I think it is too risky for my child to attend school at the moment
c) The school is not open
d) The school has asked my child not to attend
e) My child doesn't want to go
f) I think friends, family or other parents will judge me if I send my child to school
g) I think they will get a better education at home than at school at the moment
h) Being at school will be stressful for my child
i) My child's friends will not be at school
j) I have another child who can't go to school
k) I can't arrange transport to get them to school
l) There's no point, schools will shut again soon anyway
m) They have developed coronavirus symptoms (cough or fever, or change in sense of taste or smell)
n) My child developed symptoms of a different illness
o) Someone else in the family developed coronavirus symptoms (cough or fever, or change in sense of taste or smell)
p) Someone else at the school developed coronavirus symptoms (cough or fever, or change in sense of taste or smell)
q) Other reason [write in]

IF Q11A = a/b

Q15A. In this question we are interested in things that happened on the most recent day that your child went to school. Please remember that this survey is anonymous – please be honest in your answers. Please tick any that apply. [multi code]

RANDOMISE ORDER OF STATEMENTS, anchor none of these to bottom

Confidential: For Review Only

a) Either on the way to or from school, or at the school gates, I had physical contact with someone that I don't live with
b) Either on the way to or from school, or at the school gates, I was within 1 metre of someone that I don't live with for 1 minute or longer
c) Either on the way to or from school, or at the school gates, I was between 1 and 2 metres of someone that I don't live with for 15 minutes or longer
d) My child used public transport to get to or from school.
e) My child shared a lift in a car with another family to get to or from school.
f) I gave a lift in a car to a child from another family to get them to or from school.
g) My child washed their hands as soon as they got home from school.
h) My child had a shower or bath as soon as they got home from school.
i) I washed my child's clothes after they got home from school.
j) None of these [exclusive]

IF Q11A = a/b

Q16A. Thinking about the facilities or procedures at your child's school. Which of the following, if any, are actually happening as far as you are aware?

Please tick all that apply [multi code]

RANDOMISE ORDER OF STATEMENTS, anchor none of these to bottom

a) There are hand washing facilities or hand gel dispensers at the entrance to the school that are working
b) There are hand washing facilities or hand gel dispensers at the entrance to the classrooms that are working
c) Children's hand washing or hand gel use is being monitored at school
d) My child's class sizes is now 15 or fewer
e) Children need to take in their own food and snacks
f) Children are having their temperature checked on the way in
g) The school has used markings or barriers to help children keep their distance from each other
h) None of these are happening at my child's school [exclusive]

Q17. For the next series of questions, we would like you to think about one of your children who usually goes to school (that is, before the coronavirus outbreak occurred). If you have more than one child who usually went to school, then please answer about your child with the most recent birthday. If the most recent birthday was shared by more than one child (e.g. twins, or children born on the same day in different years), please answer the following questions thinking about one of these children only.

Firstly, can you tell us the first name of your child. This is just so we can refer to them throughout the survey. You can give a fake name if you would prefer. [open]

____

Q18. How old is [CHILD]? [single code]

a. 4
b. 5
c. 6
d. 7
e. 8
f. 9
g. 10
h. 11
i. 12
j. 13
k. 14
l. 15
m. 16
n. 17
o. 18

Confidential: For Review Only

Q19. What year is [CHILD] in at school [single code]

a. Reception
b. Year 1
c. Year 2
d. Year 3
e. Year 4
f. Year 5
g. Year 6
h. Year 7
i. Year 8
j. Year 9
k. Year 10
l. Year 11
m. Year 12
n. Year 13

Q20. Is [CHILD] a: [single code]

a. Boy
b. Girl

Q21. What type of school does [CHILD] normally attend? [single code]

a. Fee-paying
b. State-funded school
c. Don't know

If Q5 = a/b AND [Q4 ≠ b or Q6 = d)

Q11B. Has [CHILD] attended school at all in the past 7 days? [single code]

a. Yes, on 5 days
b. Yes, on 1 to 4 days
c. No
d. Don't know

IF Q11B = a/b

Q12B. You said that [CHILD] has attended school on at least one day in the past week. For which reasons did you send [CHILD] to school? Please tick any that apply [multi code]:

RANDOMISE ORDER OF STATEMENTS, anchor other to bottom

a) It is compulsory for my child to attend
b) I need to work
c) I have non-work commitments I need to meet
d) My child's education will benefit from being at school
e) My child will benefit from seeing their friends
f) Having my child at home is a strain
g) I don't believe my child will be at risk at school
h) My child wants to go to school
i) I think friends, family or other parents will judge me if I don't send my child to school
j) It reduces costs at home
k) Other reason [write in]

IF Q11B = b

Q13B. You said that [CHILD] has not attended school every day in the past week. For which reasons is [CHILD] attending only part-time? Please tick any that apply [multi code]:

RANDOMISE ORDER OF STATEMENTS, anchor other to bottom

a) I am using it as childcare and I only need them in part time
b) It is less risky for them to be in part-time

Confidential: For Review Only

c) The school only offers for them to be in part-time
d) I am only sending them in on days where the lessons are important
e) I am only sending them in on days when their friends are in
f) I am only sending them in on days when I can arrange transport
g) They only live with me part time
h) I started sending them in, but have now changed my mind
i) They have developed coronavirus symptoms (cough or fever, or change in sense of taste or smell)
j) My child developed symptoms of a different illness
k) Someone else in the family developed coronavirus symptoms (cough or fever, or change in sense of taste or smell)
l) Someone else at the school developed coronavirus symptoms (cough or fever, or change in sense of taste or smell)
m) Other reason [write in]

IF Q11B = c

Q14B. You said that [CHILD] has not attended school in the past week. For which reasons did you not send [CHILD] to school? Please tick any that apply [multi code]:

RANDOMISE ORDER OF STATEMENTS, anchor other to bottom

a) Someone in my household is clinically vulnerable to coronavirus
b) I think it is too risky for my child to attend school at the moment
c) The school is not open
d) The school has asked my child not to attend
e) My child doesn't want to go
f) I think friends, family or other parents will judge me if I send my child to school
g) I think they will get a better education at home than at school at the moment
h) Being at school will be stressful for my child
i) My child's friends will not be at school
j) I have another child who can't go to school
k) I can't arrange transport to get them to school
l) There's no point, schools will shut again soon anyway
m) They have developed coronavirus symptoms (cough or fever, or change in sense of taste or smell)
n) My child developed symptoms of a different illness
o) Someone else in the family developed coronavirus symptoms (cough or fever, or change in sense of taste or smell)
p) Someone else at the school developed coronavirus symptoms (cough or fever, or change in sense of taste or smell)
q) Other reason [write in]

IF Q11B = a/b

Q15B. In this question we are interested in things that happened on the most recent day that [CHILD] went to school. Please remember that this survey is anonymous – please be honest in your answers. Please tick any that apply. [multi code]

RANDOMISE ORDER OF STATEMENTS, anchor none of these to bottom

a) Either on the way to or from school, or at the school gates, I had physical contact with someone that I don't live with
b) Either on the way to or from school, or at the school gates, I was within 1 metre of someone that I don't live with for 1 minute or longer
c) Either on the way to or from school, or at the school gates, I was between 1 and 2 metres of someone that I don't live with for 15 minutes or longer
d) My child used public transport to get to or from school.
e) My child shared a lift in a car with another family to get to or from school.
f) I gave a lift in a car to a child from another family to get them to or from school.
g) My child washed their hands as soon as they got home from school.
h) My child had a shower or bath as soon as they got home from school.
i) I washed my child's clothes after they got home from school.
j) None of these [exclusive]

https://mc.manuscriptcentral.com/bmjpo

Confidential: For Review Only

IF Q11B = a/b

Q16B. Thinking about the facilities or procedures at [CHILD]'s school. Which of the following, if any, are actually happening as far as you are aware? Please tick any that apply. [multi code]

RANDOMISE ORDER OF STATEMENTS, anchor none of these to bottom

a) There are hand washing facilities or hand gel dispensers at the entrance to the school that are working
b) There are hand washing facilities or hand gel dispensers at the entrance to the classrooms that are working
c) Children's hand washing or hand gel use is being monitored at school
d) My child's class size is now 15 or fewer
e) Children need to take in their own food and snacks
f) Children are having their temperature checked on the way in
g) The school has used markings or barriers to help children keep their distance from each other
h) None of these are happening at [CHILD]'s school [exclusive]

Q22. Does [CHILD] have special educational needs? [singe code]

- Yes
- No
- Don't know / prefer not to say

Q23. In the past 7 days, how many times, if at all, has [CHILD] left your home for each of the following reasons? [multi code]

a) To go to the shops for groceries, toiletries or medicines
b) To go to the shops for other items
c) For exercise
d) For a medical need (e.g. an outpatient appointment)
e) To go to school
f) To provide help to someone else
g) To meet friends
h) To meet family members who don't live with you
i) For another reason

Q24. In the past 7 days, have you done the following, because of the risk from coronavirus:

SCALE

- Yes
- No

STATEMENTS – RANDOMISE

a. Washed your hands thoroughly and regularly
b. Stayed 2m (3 steps) away from people you do not live with when outside your home
c. Washed your clothes when you have returned home
d. Washed [CHILD]'s clothes when she/ he has returned home
e. Used tissues or clothing to touch door handles or other objects when outside
f. Cleaned or disinfected items you have brought into the home, such as groceries or parcels
g. Left items that you have brought into the home for 24 hours or more before using them, to kill any virus on them
h. Covered your face or used a face mask when out and about
i. Covered [CHILD's] face or given her/him a face mask when out and about
j. Worn protective gloves when out and about
k. Made [CHILD] wear protective gloves when out and about

Confidential: For Review Only

Q25. We are interested in how many people [CHILD] has had close contact with in the past 24 hours. By close contact we mean closer than 2 meters, for fifteen minutes or more. Please remember, this survey is anonymous so please be honest.

In the past 24 hours, how many people in the following categories has [CHILD] had close contact with: [multi code]

a. Someone [CHILD] lives with
b. Friends or other children who [CHILD] does not live with
c. A family member aged under 70 who [CHILD] does not live with
d. A family member aged over 70 who [CHILD] does not live with
e. A babysitter, nanny or childminder
f. Other children, not already reported above
g. Other adults, not already reported above

Q26. Did [CHILD] attend school in the past 24 hours? [single code]

a. Yes
b. No

RANDOMISE ORDER OF Q27a AND Q27b SHOWN TO EACH RESPONDENT

Q27a. How much, if at all, do you agree or disagree with the following statements:

STATEMENTS (randomise order):

a) If [CHILD] goes out, she/he is likely to catch coronavirus
b) If [CHILD] goes out, she/he is likely to bring coronavirus back into our home
c) [CHILD] is keeping up with her/his schoolwork
d) I feel confident helping [CHILD] with her/his schoolwork
e) I feel supported by [CHILD's] school
f) I have access to all the resources that [CHILD] needs to do her/his schoolwork
g) During lockdown, [CHILD] has learned about important things she/he wouldn't normally learn at school.
h) In the past 7 days, [CHILD] has been bored

SCALE

- Strongly agree
- Tend to agree
- Neither agree nor disagree
- Tend to disagree
- Strongly disagree
- Not applicable

Q27b. How much, if at all, do you agree or disagree with the following statements:

STATEMENTS (randomise order):

a) In the past 7 days, my household has had a regular structure to the day
b) In the past 7 days, [CHILD] has kept in touch with her/his friends
c) [CHILD] is worried about coronavirus
d) In the past 7 days, [CHILD] has felt upset about not seeing other family members who do not live with us
e) In the past 7 days, I have found it hard to keep up with work or other important commitments
f) In the past 7 days, people in my household have been getting along well
g) I am worried about the financial impact of lockdown measures
h) Before the school closures, [CHILD] had extra support at school

SCALE

- Strongly agree
- Tend to agree

Confidential: For Review Only

- Neither agree nor disagree
- Tend to disagree
- Strongly disagree
- Not applicable

Q28. To what extent, if at all, do you agree or disagree with the following statements?

RANDOMISE ORDER OF STATEMENTS

a) If it were possible, I would feel comfortable sending [CHILD] to school next week.
b) If it were possible, I would want to send [CHILD] to school next week.
c) There is still too much coronavirus around for schools to be able to reopen safely
d) When [CHILD's] school fully reopens, some parents will send their children to school, even if they have symptoms of coronavirus
e) It is impossible for children to maintain 'social distancing' at school
f) Children will not wash their hands properly at school
g) Staff will not wash their hands properly at school
h) Schools will not be able to provide good quality education at the moment
i) School will not be an enjoyable place for [CHILD] to be at the moment

SCALE

- Strongly agree
- Tend to agree
- Neither agree nor disagree
- Tend to disagree
- Strongly disagree
- Not applicable

Q29A. Has [CHILD] had any of the following symptoms in the past 7 days? Please tick any that apply, no matter where you think the symptoms came from, and even if the symptoms were mild. [multi code]

RANDOMISE ORDER OF STATEMENTS, anchor "none of these" and "don't know" to bottom

- New, continuous cough
- High temperature / fever
- Runny nose
- Diarrhoea
- Nausea / feeling sick
- Vomiting
- Sneezing
- Loss of appetite
- Loss of sense of smell (fully or partial)
- Loss of taste
- None of these
- Don't know

Q29B. Have you, or anyone else in your household (excluding [CHILD]) had any of the following symptoms in the past 14 days? Please tick any that apply, no matter where you think the symptoms came from, and even if the symptoms were mild. [multi code]

RANDOMISE ORDER OF STATEMENTS, anchor "none of these" and "don't know" to bottom

- New, continuous cough
- High temperature / fever
- Runny nose
- Diarrhoea
- Nausea / feeling sick
- Vomiting
- Sneezing
- Loss of appetite
- Loss of sense of smell (fully or partial)

Confidential: For Review Only

- Loss of taste
- None of these
- Don’t know

Q30. Do you know if [CHILD] has had, or currently has, coronavirus? (Please select the option that BEST applies) [single code]

a) They have definitely had it or definitely have it now
b) They have probably had it or probably have it now
c) They have probably not had it and probably don’t have it now
d) They have definitely not had it and definitely don’t have it now

RANDOMISE ORDER OF Q31a AND Q31b SHOWN TO EACH RESPONDENT

Q31a. Please tell us how often each of these things happen to [CHILD]. There are no right or wrong answers.
SCALE

- *Never*
- *Sometimes*
- *Often*
- *Always*

STATEMENTS
- My child worries about things
- My child worries that something awful will happen to someone in the family
- My child worries that bad things will happen to him/her
- My child feels sad or empty
- Nothing is much fun for my child anymore
- My child has trouble sleeping
- My child has problems with his/her appetite
- My child has no energy for things

Q31b. Please tell us how often each of these things happen to [CHILD]. There are no right or wrong answers.
SCALE

- *Never*
- *Sometimes*
- *Often*
- *Always*

STATEMENTS

- My child worries that something bad will happen to him/her
- My child worries about what is going to happen
- My child thinks about death
- My child is tired a lot
- My child cannot think clearly
- My child feels worthless
- My child feels like he/she doesn’t want to move
- My child feels restless

Q32. Thinking about next flu season (September 2020 – March 2021). During this time, you may be invited to vaccinate [CHILD] against flu. All primary school, but not secondary school children, are eligible for the child flu vaccine. For the following questions, please think about the next flu season.

For each of the following statements, please tell us to what extent, if at all, you agree or disagree:

I want [CHILD] to be vaccinated for flu next year (2020/21)

- Strongly agree
- Agree
- Neither agree nor disagree

Confidential: For Review Only

- Disagree
- Strongly disagree
- Not applicable, [CHILD] will be in secondary school in next school year (2020/21)

IF Q32 = a-e

Q33 I intend [CHILD] to be vaccinated for flu next year (2020/21)

- Strongly agree
- Agree
- Neither agree nor disagree
- Disagree
- Strongly disagree
- Not applicable, [CHILD] will be in secondary school in next school year (2020/21)

Thinking now about yourself…

Q34. Over the last 2 weeks, how often have you been bothered by the following problems?

SCALE

- Not at all
- Several days
- More than half the days
- Nearly every day

STATEMENTS

- Feeling nervous, anxious or on edge
- Not being able to stop or control worrying
- Little interest or pleasure in doing things
- Feeling down, depressed, or hopeless

And finally, the following questions ask for some more information about you and your household.

D2. Which gender do you identify yourself with? [single code]

- Male
- Female
- Prefer to self-describe
- Prefer not to say

D3. What is your employment status? [single code]

a. Full time paid job (31+ hours)
b. Part time paid job (<31 hours)
c. Doing paid work on a self-employed basis or within your own business
d. Employed, but currently furloughed
e. Student / On a government training programme (Nation Traineeship/Modern Apprenticeship)
f. Out of work (6 months or less)
g. Out of work (more than 6 months)
h. Looking after home / Homemaker
i. Retired
j. Disabled OR Long-term sick
k. Unpaid work for a business, community or voluntary organisation
l. Prefer not to say

If answer a/b/c to D3

D4. Are you currently working from home? [single code]

- Yes
- No

https://mc.manuscriptcentral.com/bmjpo

Confidential: For Review Only

D5. What is the highest level of educational qualification you have received? [single code]

a. PhD/Doctor
b. Master's
c. Bachelor's Degree or equivalent (Such as a NVQ level 5)
d. Higher education (Such as a HND or a NVQ level 4)
e. A level or equivalent (Such as Scottish Highers or NVQ level 3)
f. GCSE and below (Such as O level or an RSA Diploma)
g. Other qualifications (Such as NVQ level 1)
h. No qualifications
i. Prefer not to say

D6. Which of the following income brackets best represents your household income, before tax deductions for income tax, National Insurance etc? [single code]

a. Less than £5,000
b. £5,000-£9,999
c. £10,000-£14,999
d. £15,000-£19,999
e. £20,000-£24,999
f. £25,000-£29,999
g. £30,000-£34,999
h. £35,000-£39,999
i. £40,000-£44,999
j. £45,000-£49,999
k. £50,000-£59,999
l. £60,000-£69,999
m. £70,000-£84,999
n. £85,000-£99,999
o. More than £100,000
p. Prefer not to say

D7. What is your current marital or civil partnership status? [single code]

a. Single (i.e. never married and never registered as a same sex civil-partnership)
b. Co-habiting with partner (but never married or been in a civil partnership)
c. Civil partnership
d. Married
e. Separated, but still legally married / in a civil partnership
f. Divorced / Civil partnership legally dissolved
g. Widowed / Surviving partner from a same-sex civil partnership

D8. Which of the following categories would best describe your ethnicity? [single code]

a. English/Welsh/Scottish/Northern Irish/British
b. Irish
c. Gypsy or Irish Traveller
d. Other
e. White and Black Caribbean
f. White and Black African
g. White and Asian
h. Other
i. Indian
j. Pakistani
k. Bangladeshi
l. Chinese
m. Other
n. Caribbean
o. African
p. Other
q. Arab
r. Any other (please specify)
s. Prefer not to say

Confidential: For Review Only

D10. How many children in each age group live in your household? [number list]

- 0-3
- 4-10
- 11-15
- 16-18

Allow numerical input for each age group

D11. Do you live with anyone over the age of 70 years? [single code]

a. Yes
b. No

D12. Including yourself, how many people live in your household? [single code]

a. 2
b. 3
c. 4
d. 5
e. 6
f. 7
g. 8
h. 9
i. 10 or more

D9. Do any of the following have a medical condition than might make them particularly vulnerable to coronavirus?

SCALE

- Yes
- No
- Don't know

STATEMENTS

a. Yourself
b. [CHILD]
c. Anyone else you live with [show if D12 = b-i]

D13. [Record number] How many bedrooms does your home have?

D14. [Multi code] Which of the following do you have access to at your home:

a) A garden
b) A patio / terrace
c) A balcony
d) Another form of outdoor space
e) No outdoor space

Thank you for completing this survey. If you have any concerns about your child's mental health, please click here.

Confidential: For Review Only
